# Comparison of exclusion, imputation and modelling of missing binary outcome data in frequentist network meta-analysis

**DOI:** 10.1186/s12874-020-00929-9

**Published:** 2020-02-28

**Authors:** Loukia M. Spineli, Chrysostomos Kalyvas

**Affiliations:** 1 Midwifery Research and Education Unit (OE 6410), Hannover Medical School, Carl-Neuberg-Straße 1, 30625 Hannover, Germany; 2 Department of Biostatistics and Research Decision Sciences, MSD Europe Inc, Clos du Lynx 5, 1200 Brussels, Belgium

**Keywords:** Missing outcome data, Network meta-analysis, Pattern-mixture model, Imputation, Systematic review

## Abstract

**Background:**

Missing participant outcome data (MOD) are ubiquitous in systematic reviews with network meta-analysis (NMA) as they invade from the inclusion of clinical trials with reported participant losses. There are available strategies to address aggregate MOD, and in particular binary MOD, while considering the missing at random (MAR) assumption as a starting point. Little is known about their performance though regarding the meta-analytic parameters of a random-effects model for aggregate binary outcome data as obtained from trial-reports (i.e. the number of events and number of MOD out of the total randomised per arm).

**Methods:**

We used four strategies to handle binary MOD under MAR and we classified these strategies to those modelling versus excluding/imputing MOD and to those accounting for versus ignoring uncertainty about MAR. We investigated the performance of these strategies in terms of core NMA estimates by performing both an empirical and simulation study using random-effects NMA based on electrical network theory. We used Bland-Altman plots to illustrate the agreement between the compared strategies, and we considered the mean bias, coverage probability and width of the confidence interval to be the frequentist measures of performance.

**Results:**

Modelling MOD under MAR agreed with exclusion and imputation under MAR in terms of estimated log odds ratios and inconsistency factor, whereas accountability or not of the uncertainty regarding MOD affected intervention hierarchy and precision around the NMA estimates: strategies that ignore uncertainty about MOD led to more precise NMA estimates, and increased between-trial variance. All strategies showed good performance for low MOD (<5%), consistent evidence and low between-trial variance, whereas performance was compromised for large informative MOD (> 20%), inconsistent evidence and substantial between-trial variance, especially for strategies that ignore uncertainty due to MOD.

**Conclusions:**

The analysts should avoid applying strategies that manipulate MOD before analysis (i.e. exclusion and imputation) as they implicate the inferences negatively. Modelling MOD, on the other hand, via a pattern-mixture model to propagate the uncertainty about MAR assumption constitutes both conceptually and statistically proper strategy to address MOD in a systematic review.

## Background

Recent empirical studies on systematic reviews of randomised controlled trials with at least two interventions have revealed the ubiquity of missing participant outcome data (MOD) in at least one included trial [1–4]. Modelling and data-manipulation strategies have been both proposed and applied to address MOD in a meta-analysis [5]. Modelling revolves around the joint likelihood of observed and missing outcomes and the indicator of observing an outcome [6]; by conditioning on the indicator of observing an outcome or on the underlying outcome, we obtain the pattern-mixture model and the selection model, respectively [7–11]. In contrast, data-manipulation strategies are based exclusively either on a degenerate probability distribution [6] – when they aim to impute a single value under a specific scenario to compensate for the missing outcomes in each arm of every trial – or on the exclusion of MOD in order to approximate the missing at random (MAR) assumption which implies the distribution of the outcome to be the same in completers and missing participants conditional on the observed variables [10, 12, 13]. In the present study, modelling and data-manipulation strategies refer to aggregate binary outcome data, that is, summary data from each arm of every trial (the number of events and number of MOD out of the total randomised per arm) as obtained from published trial-reports.

Data-manipulation strategies have thrived in systematic reviews with meta-analyses or network meta-analyses (NMA) for being intuitive and straightforward to apply as they require no sophisticated statistical software [1–4, 13]. Nevertheless, their simplicity comes with the price of challenging the credibility of conclusions. Specifically, imputation of MOD mostly lacks plausibility due to the use of a degenerate probability distribution (i.e. the imputed values would have occurred with certainty [6]) which raises the risk of providing biased results with spurious precision as it naturally ignores uncertainty around the assumptions made [6]. Moreover, if MOD are substantial and the mechanism behind missingness is non-ignorable, then exclusion of MOD also risks providing biased results [8, 10].

Imputation scenarios may be arm-specific or common for all arms in a trial, but they are customarily applied the same across all trials included in a meta-analysis [1, 2, 4, 12, 13]. In practice, imputation hardly ever includes clinically plausible scenarios that comply with the condition and interventions investigated. Instead, extreme scenarios constitute the general rule which, in the case of binary outcomes, replace all MOD either with or without the occurrence of the outcome before analysis [1, 2, 4, 13]. It is, therefore, recommended that reviewers choose scenarios tailored to the investigated condition and interventions with increasing stringency to evaluate the robustness of meta-analysis results to departures from MAR assumption in the primary analysis [10, 14–16].

Contrary to data-manipulation, modelling MOD is conceptually and statistically advantageous, as it quantifies the plausible relationship between missing and observed outcomes – rather than adjusting the dataset before analysis – and it incorporates the uncertainty about that relationship [7, 8, 10]. Consequently, in each trial, treatment effects and standard errors are adjusted for MOD, and this adjustment carries over to meta-analysis estimates. Depending also on the extent of MOD, accountability of uncertainty due to MOD results in relatively larger standard errors of treatment effects but lower between-trial variance [7, 17]; this is the tradeoff of modelling MOD in random-effects meta-analysis.

The research agenda of NMA, an extension of pairwise meta-analysis for multiple interventions [18], has been refined considerably the last decade with plenty methodological articles, hands-on and software tutorials, empirical and simulation studies using Bayesian and frequentist methods [19–21]. While Bayesian methods constitute the norm in published systematic reviews with NMA, frequentist approaches have also drawn the attention of many methodologists recently [20]. In the present study, we extended the data-manipulation and modelling strategies, as used in the meta-analysis, to operate in a network of interventions within a frequentist framework [22, 23]. We focused only on aggregate binary outcomes for being the most frequently investigated outcome in systematic reviews [19, 24], and we considered the MAR assumption for being recommended as a ‘starting point’ in the primary analysis [8, 10, 25]. Ultimate objectives of this study were to direct the attention of reviewers to the implications on the NMA estimates of various data-manipulation strategies for binary MOD under MAR as compared to modelling MOD and to provide recommendations for good practice.

The present article has been structured as follows. In Section “Methods”, we first review the data-manipulation strategies and modelling that we considered under MAR and then, we describe the dataset we used to perform the empirical comparisons and the tools we applied to illustrate the results. In Section “Results of the empirical study”, we present the results of the empirical analyses. In Section “Simulation study”, we describe the set-up of the simulation study to supplement the results from the empirical analyses and in Section “Results of the simulation study”, we present the simulation results. In Section “Discussion”, we discuss our findings and highlight important limitations and in Section “Conclusions”, we conclude with recommendations for practice.

## Methods

### Addressing binary MOD under MAR

Suppose a network of *N* trials comparing different sets of *T* interventions for a patient-important binary outcome [26]. We observe the number of events in arm *k* of trial *i*, *r*_*i*, *k*_, and the number of MOD, *m*_*i*, *k*_, out of the number randomised, *n*_*i*, *k*_. Four strategies have been described to address MOD under MAR [8, 10, 13]. These strategies differ not only on how MOD are handled (i.e. imputed, excluded or modelled) but also on whether and how uncertainty due to MOD is addressed. We delineate these strategies at trial-level to obtain log odds ratios (OR) and standard errors that will be fed into the frequentist random-effects NMA model as described by Rücker [23] and Rücker and Schwarzer [22] in the context of electrical network theory.

#### Exclusion of MOD and ignorance of uncertainty due to MOD

Exclusion of MOD before analysis is a common data-manipulation strategy in systematic reviews either as sensitivity or primary analysis [1–4, 13]. We call this strategy ‘complete case analysis’ (CCA). CCA implies MAR, and therefore, excludes missing participants from the randomised sample – an approach that contradicts the desired intention-to-treat principle in clinical trials [15, 27] (i.e. those randomised should be analysed regardless of withdrawal or intervention received) and may lead to biased results if not valid [10]. Under CCA, the log OR of an event between arm *k* and the baseline arm of trial *i* is estimated after restricting the analysed sample to those completing the trial, *n*_*i*, *k*_ − *m*_*i*, *k*_:

1 $$ {y}_{i,k1}= logit\left({r}_{i,k}/\left({n}_{i,k}-{m}_{i,k}\right)\right)- logit\left({r}_{i,1}/\left({n}_{i,1}-{m}_{i,1}\right)\right) $$

with variance approximated by

$$ {v}_{i,k1}=\left(1/{r}_{i,k}\right)+\left(1/{r}_{i,1}\right)+\left(1/\left({n}_{i,k}-{r}_{i,k}-{m}_{i,k}\right)\right)+\left(1/\left({n}_{i,1}-{r}_{i,1}-{m}_{i,1}\right)\right) $$

In the case of zero events in trial *i*, a continuity correction of 0.5 is commonly applied to all cells of the *a*_*i*_ × 2 table where *a*_*i*_ is the number of arms in trial *i* [28].

#### Exclusion of MOD but accountability of uncertainty due to MOD

Gamble and Hollis introduced the ‘uncertainty interval’, a hybrid of the confidence interval for the within-trial log ORs as estimated after excluding missing participants (Eq. (1)) to reflect the uncertainty stemming from having missing participants in addition to sampling error [13]. ‘Uncertainty interval’ is calculated for each trial and it results from the lowest and uppermost bound of 95% confidence interval for the within-trial log OR under the best- and worst-case scenarios (i.e. all missing participants experienced and did not experience the beneficial outcome in the active arm, respectively, as opposed to the control arm). Being a product of the most extreme scenarios, ‘uncertainty interval’ is wider than the 95% confidence interval and thus, the former provides smaller weights than the latter in the presence of MOD [13].

#### Modelling MOD using a two-stage pattern-mixture model

Instead of excluding MOD before analysis, we can model MOD using the pattern-mixture model which is an elegant and statistically appropriate approach as it adjusts the within-trial treatment effects for potential bias due to MOD and it accounts for the uncertainty due to MOD. The within-trial adjustments constitute the first stage [8]. In the case of zero events, a continuity correction of 0.5 is used before adjustment, as described in Section “Exclusion of MOD and ignorance of uncertainty due to MOD”. Then, at the second stage, the adjusted within-trial results (i.e. log OR and standard error) constitute the dataset to apply random-effects NMA (see, Section “Model specification”) [8].

Under this model, the underlying probability of an event in arm *k* of trial *i*, *p*_*i*, *k*_, is equated with the sum of marginal probability of observing an event (*Z*_*i*, *k*, *l*_ = 1, *R*_*i*, *k*, *l*_ = 1) and the marginal probability of missing an event (*Z*_*i*, *k*, *l*_ = 1, *R*_*i*, *k*, *l*_ = 0):

2 $$ {\displaystyle \begin{array}{c}{p}_{i,k}=P\left({Z}_{i,k,l}=1,{R}_{i,k,l}=1\right)+P\left({Z}_{i,k,l}=1,{R}_{i,k,l}=0\right)\\ {}=P\left({Z}_{i,k,l}=1|{R}_{i,k,l}=1\right)\cdot P\left({R}_{i,k,l}=1\right)+P\left({Z}_{i,k,l}=1|{R}_{i,k,l}=0\right)\cdot P\left({R}_{i,k,l}=0\right)\\ {}={p}_{i,k}^c\cdot \left(1-{q}_{i,k}\right)+{p}_{i,k}^m\cdot {q}_{i,k}\end{array}} $$

where *Z*_*i*, *k*, *l*_ indicates the occurrence of an event for participant *l* (*l* = 1, 2, …, *n*_*i*, *k*_) in arm *k* of trial *i*, *R*_*i*, *k*, *l*_ indicates whether participant *l* completed arm *k* of trial *i*, ${p}_{i,k}^c$ is the probability of event conditional on the completers, ${p}_{i,k}^m$ is the probability of event conditional on missing participants (the missingness parameter) and *q*_*i*, *k*_ is the probability of MOD in arm *k* of trial *i*.

If we have some prior belief regarding the association between outcome and status of a participant being missing or observed, then a relative missingness parameter, such as the informative missingness odds ratio (IMOR), may be preferred to the absolute ${p}_{i,k}^m$[7]. IMOR is the ratio of the odds of an event among MOD to the odds of an event among completers [7, 8, 10]. After replacing ${p}_{i,k}^m$ with the IMOR parameter, ${e}^{\delta_{i,k}}$, in Eq. (2) we obtain:

3 $$ {p}_{i,k}={p}_{i,k}^c\bullet \left(1-{q}_{i,k}\right)+\frac{p_{i,k}^c\bullet {e}^{\delta_{i,k}}}{p_{i,k}^c\bullet {e}^{\delta_{i,k}}+1-{p}_{i,k}^c}\bullet {q}_{i,k} $$

Then, our prior belief about the missingness process can be quantified via a normal distribution for log IMOR (i.e. *δ*_*i*, *k*_) with mean *Δ*_*i*, *k*_ reflecting our belief on average and variance *V*_*i*, *k*_ indicating our uncertainty about this belief [7, 8]:

4 $$ {\delta}_{i,k}\sim N\left({\varDelta}_{i,k},{V}_{i,k}\right)\ i=1,2,\dots, N\ \mathrm{and}\ k=1,2,\dots, {a}_i $$

Under MAR, *Δ*_*i*, *k*_ = 0 and we call this strategy ‘on average MAR’. In practice, *V*_*i*, *k*_ can be considered constant and equal to any positive value up to four; otherwise, *v*_*i*, *k*1_ becomes inaccurate using the Taylor series approximation (Fig. 2 in White et al. [8]). In the present study, we used *V*_*i*, *k*_ = 1.

Under ‘on average MAR’, *p*_*i*, *k*_ in Eq. (3) corresponds to *r*_*i*, *k*_/(*n*_*i*, *k*_ − *m*_*i*, *k*_) in Eq. (1). Now, *v*_*i*, *k*1_ needs to accommodate two sources of variance: one due to sampling error and one arising from *δ*_*i*, *k*_. Following White et al. [8] the variance due to sampling error can be approximated using Taylor series (Eq. (13) in White et al. [8]), whereas the variance due to *δ*_*i*, *k*_ can be approximated using Eq. (16) in White et al. [8] and assuming zero correlation between log IMORs of the compared arms.

Note that in a strict sense, the selection model directly reflects the taxonomy of missingness mechanisms (i.e. missing completely at random (MCAR), MAR, and missing not at random) according to Little and Rubin [29]. For the definition of MCAR and MAR in a series of trials for two interventions via the selection model, we direct the readership to White et al. [9] (Eqs. 2 and 3, respectively, there).

#### Imputing the same risk as observed and ignoring uncertainty due to MOD

Using the pattern-mixture model and assuming that both missing participants and completers have the same risk to experience the event (MAR assumption), we can replace ${p}_{i,k}^m$ with ${p}_{i,k}^c$ in Eq. (2), and obtain ${p}_{i,k}={p}_{i,k}^c$. We call this data-manipulation strategy ‘imputed case analysis of observed event risks’ (ICAp, as in Higgins et al. [10]). Then, the log OR of trial *i* is obtained using Eq. (1), and the variance is calculated based on the randomised sample as follows:

$$ {v}_{i,k1}=\frac{1}{n_{i,k}\bullet {p}_{i,k}\bullet \left(1-{p}_{i,k}\right)}+\frac{1}{n_{i,1}\bullet {p}_{i,1}\bullet \left(1-{p}_{i,1}\right)} $$

Contrary to CCA, this strategy maintains the randomised sample in each arm of every trial and therefore, it reduces the standard error because the imputed risks are mistreated as observed. Based on empirical studies, the prevalence of this strategy in systematic reviews with two interventions ranges from 1 to 6% [1, 2, 4].

While *y*_*i*, *k*1_ s will be the same in all four strategies, the corresponding *v*_*i*, *k*1_ s will differ to some degree, and consequently, they will affect the estimation of NMA log ORs and their standard errors.

### An empirical investigation of the strategies

We considered ‘on average MAR’ to be the reference strategy for being conceptually and statistically appropriate. We compared ‘on average MAR’ with the other three strategies in terms of (i) NMA log ORs of the comparisons with the selected reference intervention of the network and their standard error, (ii) (common within the network) between-trial variance, *τ*^2^, (iii) inconsistency factors (IF) and their standard error obtained via the back-calculation approach [30], and (iv) P-score [31] which is the frequentist equivalent of the surface under the cumulative ranking curve (SUCRA) value (it reflects the percentage of potency (e.g. effectiveness or safety) of each intervention when compared to an imaginary intervention that always ranks first with certainty on the investigated outcome) [32].

#### Analysed dataset of systematic reviews with NMA

To perform this empirical study, we used our collection of 29 systematic reviews with NMA on patient-important binary outcomes from 12 different health-related fields [33]. Initially, for each network, we compared the median of the total percentage of MOD (%MOD) across the included trials with the ‘five-and-twenty rule’ as proposed by Sackett et al. [34] and we considered MOD to be low for median less then 5%, moderate for median at least 5% and up to 20% and large for median above 20% [33]. Subsequently, we divided each network to trials with balanced and trials without balanced MOD in the compared arms according to the two-sided Pearson’s chi-squared test statistic (we tested the null hypothesis that the difference in %MOD between the compared arms in each trial is zero) and we used a density plot to visualise the distribution of the differences in %MOD for each group of trials: the two densities intersected at 6.5% [33]. Then, for each network, we compared this threshold with the median of the difference in %MOD between the compared arms across the included trials: networks with median larger than 6.5% were considered to have an imbalance in MOD. According to this decision rule to characterise the amount of MOD in a network, we distinguished the networks to those with ‘low MOD’ (41%), ‘moderate and balanced MOD’ (48%), ‘moderate and unbalanced MOD’ (7%), ‘large and balanced MOD’ (0%), and ‘large and unbalanced MOD’ (4%) [33]. We re-structured the dataset of each network by recoding the outcome so that OR more than 1 indicated a beneficial effect for the first intervention in each comparison [33].

#### Bland-Altman plots to investigate the agreement

To illustrate the level of agreement between ‘on average MAR’ and the other strategies in terms of the NMA estimates, we used Bland-Altman plots [35, 36]. For each NMA estimate, we plotted the differences between ‘on average MAR’ and the other strategies against their averages. For the standard error of log ORs and IFs, we plotted the ratios of the estimates from the compared strategies against their averages. On the y-axis, we displayed the average bias (i.e. mean of the differences or mean of log ratios exponentiated) alongside the 95% limits of agreement (LoA) [35, 36]. We considered the compared strategies to have a good agreement when the average bias for a specific NMA estimate was approximating 0 (for differences) or 1 (for ratios) and most of the points were uniformly scattered around the average bias within narrow LoA. To construct the Bland-Altman plots, we used the statistical software R version 3.3.1 [37] where we wrote user-defined functions while using the R package *ggplot2* [38].

#### Cohen’s kappa statistic to measure agreement

We used the Cohen’s kappa statistic [39] to compare ‘on average MAR’ with the other strategies in terms of strength and direction of log ORs and IFs as well as in terms of the extent of *τ*^2^ in each network. To define the extent of *τ*^2^ in each network, we referred to the predictive distributions as elicited by Turner et al. [40], and we judged the median of *τ*^2^ to be low, moderate and large, if it was below the second quartile, between the second and third quartile and above the third quartile of the selected predictive distribution, respectively. We used the divisions of the agreement as reported in Landis and Koch to infer on the degree of agreement [41].

#### Model specification

For each network, we used the four strategies described aboved to obtain the within-trial log ORs and standard errors, and then, we applied the random-effects NMA model as described by Rücker [23] and Rücker and Schwarzer [22] using electrical network theory. We used the R package *netmeta* to fit all NMA models [42]. For the estimation of *τ*^2^, *netmeta* uses the generalisation of DerSimonian and Laird’s procedure in the multivariate setting as proposed by Jackson et al. [43]. The dataset used for the empirical comparisons can be found in Additional file 1. The R scripts applied to convert the dataset into a contrast-level long format to implement the four strategies and then to be used in the *netmeta* function can be found in Additional file 2.

## Results of the empirical study

**Fig. 1:**
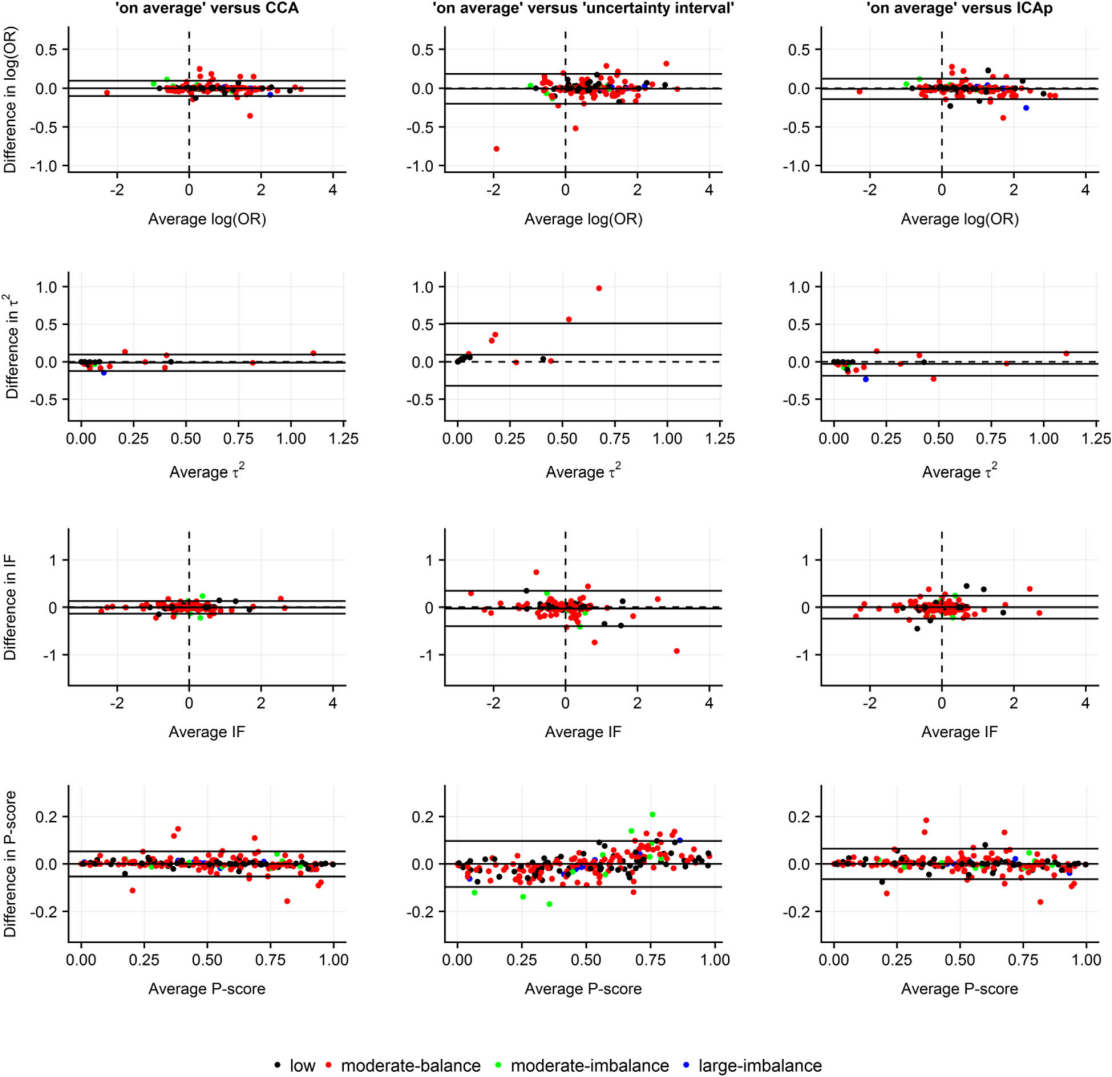
Bland-Altman plots based on the empirical analysis of 29 networks illustrate the level of agreement between ‘on average MAR’ and other strategies for MOD under MAR assumption in terms of estimated log odds ratios (of comparisons with the selected reference intervention of each network), common between-trial variance (*τ*^2^), inconsistency factors, and P-scores. Use of normal distribution on log IMORs with mean 0 and variance 1. Different colours indicate the extent and balance of MOD across 29 networks (17 networks with at least one closed loop). CCA, complete case analysis; ICAp, imputed case analysis of observed event risks; IF, inconsistency factor; OR, odds ratio

‘On average MAR’ appeared to agree with both CCA and ICAp in all NMA estimates, though the differences in the point estimates tended to range in slightly narrower LoA for ‘on average MAR’ versus CCA (Fig. 1). Despite the relatively low average bias, the agreement between ‘on average MAR’ and ‘uncertainty interval’ was inadequate overall, as the differences in the point estimates were scattered within substantially wide LoA that reflected discrepancies between these strategies (Fig. 1). Furthermore, ‘uncertainty interval’ led to systematically smaller *τ*^2^ s as compared to ‘on average MAR’. Interestingly, ‘uncertainty interval’ led also to systematically smaller and larger P-scores for interventions that ranked high or very low in the hierarchy, respectively, as compared to ‘on average MAR’, especially for moderate and large missingness (Fig. 1).

**Fig. 2:**
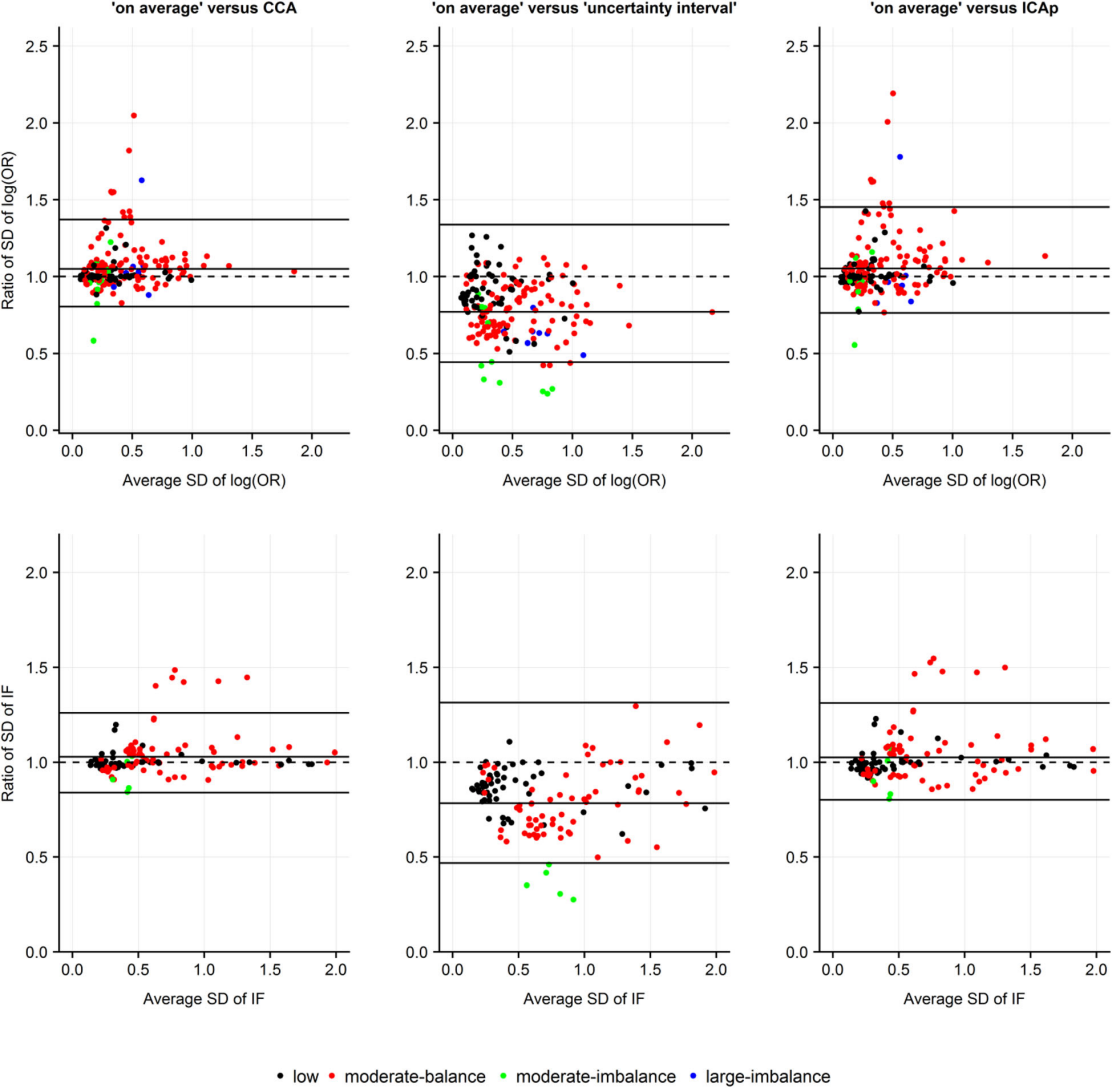
Bland-Altman plots based on the empirical analysis of 29 networks illustrate the level of agreement between ‘on average MAR’ and other strategies for MOD under MAR assumption in terms of the standard error of log odds ratios (of comparisons with the selected reference intervention of each network) and of inconsistency factors. Use of normal distribution on log IMORs with mean 0 and variance 1. Different colours indicate the extent and balance of MOD across 29 networks (17 networks with at least one closed loop). CCA, complete case analysis; ICAp, imputed case analysis of observed event risks; IF, inconsistency factor; OR, odds ratio

As expected, ignoring the uncertainty about MAR – either via CCA or ICAp – led to relatively smaller standard errors of log ORs and IFs, especially for moderate and large missingness in case of CCA, compared to ‘on average MAR’, as most points were scattered above the line of no difference – though within a wider LoA for ‘on average MAR’ versus ICAp (Fig. 2). However, when uncertainty about MOD was considered, ‘uncertainty interval’ led to larger standard errors in both NMA estimates, especially for moderate and large missingness, as opposed to ‘on average MAR’ (average bias: 0.77 and 0.78, for standard error of log OR and IF, respectively) (Fig. 2).

Overall, there was good agreement in strength and direction of log ORs, as well as in the direction of IFs, except for the strength of IFs where the agreement was poor overall (Supplementary Table 1, Additional file 3). The level of agreement in the extent of *τ*^2^ could not be judged with confidence due to few estimated *τ*^2^ s (only 29).

## Simulation study

To supplement the results from Section “Results of the empirical study”, we additionally conducted a comprehensive simulation study. We followed the simulation set-up of our previous work for triangles of two arm-trials comparing placebo, old and new intervention [44], where we used the data generating model (DGM) as proposed by Hartung and Knapp for a random-effects pairwise meta-analysis [45]. We considered new versus old intervention to be the comparison of interest.

### Simulation scenarios using empirical evidence

To determine the trial size (same in the compared arms), the event risks for the control arms, and the extent of the inconsistency, we used the information from the networks collected in the previous empirical work [33]. Following Veroniki et al. [46], we assumed a typical loop with four trials for old intervention versus placebo, three trials for new intervention versus placebo, and one trial for new versus old intervention and we doubled the number of trials in another scenario (Table 1). To define the extent of *τ*^2^ in each arm, we considered smaller variability in log odds for placebo, whereas equal variability in log odds for active arms [44]. We investigated two scenarios for *τ*^2^; small and substantial as reflected by the median of the predictive log-normal distributions *LN*(–3.95, 1.34^2^) for all-cause mortality and *LN*(–2.56, 1.74^2^) for a generic healthcare setting, respectively [40]. To determine the ‘true’ P-score for each intervention, we initially ordered the true log ORs for the placebo comparisons generated from the normal distribution *N*(*μ*_*kP*_, *τ*^2^) with *μ*_*NP*_ =  *log* (2) and *μ*_*OP*_ =  *log* (1.5) being the true log ORs for new and old intervention against placebo, respectively. Then, for each intervention, we calculated the probability of reaching a specific rank and, subsequently, we applied the formula for the SUCRA score as described in Salanti et al. [32].

**Table 1:** Scenarios considered for the simulation set-up

*Number of trials per comparison*	
typical loop^a^	*NO* = 1, *NP* = 3, *OP* = 4
double	*NO* = 2, *NP* = 6, *OP* = 8
*Trial size*	
placebo-controlled trials	*Unif*(102, 187)
old-controlled trials	*Unif*(128, 241)
*Initial event rates of control arm*	
placebo-controlled trials	*Unif*(0.27, 0.40)
old-controlled trials	*Unif*(0.63, 0.76)
*Balanced risk of missing outcome data*	
low	*Unif*(0, 0.04)
moderate	*Unif*(0.05, 0.20)
large	*Unif*(0.21, 0.40)
*Unbalanced risk of missing outcome data*	
low	*Unif*(0, 0.04)^b^
moderate	*Unif*(0.05, 0.10) for E, *Unif*(0.11, 0.20) for C
large	*Unif*(0.21, 0.30) for E, *Unif*(0.31, 0.40) for C
*Missingness mechanisms* via *log (IMOR)*	
informative	*TN*(*μ* = − *ln* (2), *σ*^2^ = 1, *a* = *ln* (1)) for Placebo
	*TN*(*μ* = *ln* (2), *σ*^2^ = 1, *a* = *ln* (1)) for New and Old
missing at random	*N*(0, 1) for all interventions
*Treatment effects*	
basic comparisons	*LOR*_*NP*_ = *ln* (2), *LOR*_*OP*_ = *ln* (1.5)
functional comparison	*LOR*_*NO*_ = *LOR*_*NP*_ − *LOR*_*OP*_ + *IF*
*Loop inconsistency*	
inconsistency factor (IF)^c^	*IF* = absent *IF* = moderate
*Common between-trial variance*	
predictive distribution^d^	*τ*^2^ = 0.02 (small) *τ*^2^ = 0.08 (substantial)
*Surface under cumulative ranking curve*	
new intervention	96 and 88% for small and substantial *τ*^2^, respectively
old intervention	54 and 58% for small and substantial *τ*^2^, respectively
placebo	0 and 4% for small and substantial *τ*^2^, respectively

Note: *C* control arm, *E* experimental arm, *IF* consistency factor, *IMOR* informative missingness odds ratio, *LOR* log odds ratio, *N* normal distribution, *NO* New intervention versus Old intervention, *NP* New intervention versus Old intervention, *OP* Old intervention versus Placebo, *TN* truncated-normal distribution, *Unif* uniform distribution

^a^As defined in Veroniki et al. [46]

^b^In the presence of low missing outcome data, imbalance of missing outcome data in the compared arms is negligible, and therefore, in both arms the risk of missingness was generated from *U*(0, 0.04) irrespectively the type of intervention

^c^Absent and moderate inconsistency refer to the mean of t-distributions *t*(*μ* = 0, *σ*^2^ = 0.44^2^, *df* = 3) and *t*(*μ* = 1, *σ*^2^ = 0.44^2^, *df* = 3), respectively

^d^Small and substantial *τ*^2^ refer to the predictive log-normal distributions *LΝ*(−3.95, 1.34^2^) for all-cause mortality and *LΝ*(−2.56, 1.74^2^) for generic health setting, respectively [40]

To accommodate MOD in the DGM, we followed the ‘five-and-twenty rule’ proposed by Sackett et al. [34], and we considered MOD to be low (0–4%), moderate (5–20%) and large (> 20%) in each arm of every trial. Furthermore, in one scenario we considered an equal risk of MOD in the compared arms (balanced MOD) and in another scenario, we assumed a higher risk of MOD for placebo, as well as for old intervention in trials comparing new with old intervention. We assumed patients randomised in new or old intervention to be on average twice more likely to leave the trial due to improvement as opposed to patients receiving placebo. In another scenario, we assumed MAR for all interventions. We used log IMOR to quantify the degree of informative missingness and we incorporated it in a pattern-mixture model to generate MOD (Eq. (3)). Table 1 summarises the scenarios considered for the simulation study.

### Model specification and illustration of results

For each scenario, we simulated 5000 triangles, and we analysed the generated datasets applying the strategies described in Section “Addressing binary MOD under MAR” to estimate the log OR, *τ*^2^, IF, and P-score for each intervention. We investigated the mean bias (MB) for all NMA estimates, as well as the 95% coverage probability and width of the 95% confidence interval for log OR and IF. Simulations and analyses were performed in the statistical software R version 3.3.1 [37] using the R package *netmeta* [42] to employ the frequentist NMA for each strategy. We used the R package *ggplot2* [38] to create a matrix of panels with the simulation results, where each panel referred to a specific scenario. The simulation code to generate and analyse the triangle networks can be found in Additional file 4.

## Results of the simulation study

We present the results on informative MOD with a moderate and large extent, as it is a more plausible scenario in a medical setting. Results for MAR (Supplementary Figure 7–16, Additional file 5) or low MOD (Supplementary Figure 17–26, Additional file 5) can be found in Additional file 5.

### Mean bias

#### Log OR between new and old intervention

**Fig. 3:**
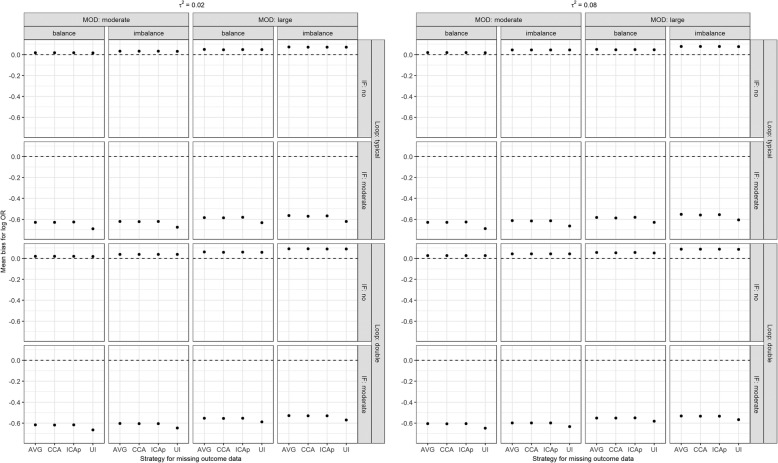
Mean bias for log OR (new versus old intervention) under informative MOD while accounting for the scenarios on the number of studies (typical loop, double), the extent of MOD (moderate, large), the balance of MOD (balance, imbalance), the extent of between-trial variance (*τ*^2^; 0.02 as small, 0.08 as substantial), and extent of inconsistency (absent, moderate). AVG, on average MAR; CCA, complete case analysis; ICAp, imputed case analysis of observed event risks; IF, inconsistency factor; MOD, missing outcome data; UI, uncertainty interval

When moderate MOD were balanced, and consistency regulated the network, all strategies had almost zero MB for log OR (range: 0.02–0.03); however, for large or unbalanced MOD, log OR was similarly overestimated across all strategies – most notably for large and unbalanced MOD (Fig. 3). In the presence of inconsistency, log OR was substantially underestimated in all strategies. Overall, the loop size and/or the magnitude of *τ*^2^ did not implicate the results.

#### Common between-trial variance

**Fig. 4:**
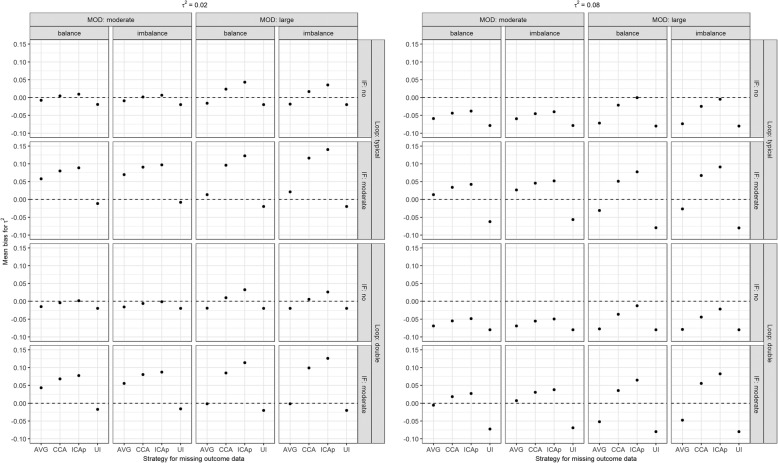
Mean bias for between-trial variance (*τ*^2^) under informative MOD while accounting for the scenarios on the number of studies (typical loop, double), the extent of MOD (moderate, large), the balance of MOD (balance, imbalance), and extent of inconsistency (absent, moderate). AVG, on average MAR; CCA, complete case analysis; ICAp, imputed case analysis of observed event risks; IF, inconsistency factor; MOD, missing outcome data; UI, uncertainty interval

In the presence of consistency and small *τ*^2^, MB for *τ*^2^ was low in all strategies for moderate MOD, but increased slightly in CCA and notably in ICAp for large MOD (Fig. 4). However, when true *τ*^2^ was substantial, *τ*^2^ was underestimated in all strategies, though negligibly in ICAp but markedly in ‘on average MAR’ and ‘uncertainty interval’ for large MOD. In the absence of consistency, *τ*^2^ was substantially overestimated in CCA and ICAp, especially for small *τ*^2^ and large, unbalanced MOD, while ‘uncertainty interval’ slightly underestimated *τ*^2^ but more notably for large MOD and substantial *τ*^2^. Using ‘on average MAR’, MB for *τ*^2^ was somewhere in-between in all scenarios. When the typical loop was doubled, MB for *τ*^2^ decreased slightly in all scenarios and strategies.

#### Inconsistency factor

Under consistency, MB for IF was slightly positive and similar in all strategies for moderate, balanced MOD (range: 0.01–0.03), but increased further for large, balanced MOD (range: 0.05–0.07) (Supplementary Figure 1, Additional file 5). Contrariwise, IF was underestimated for unbalanced MOD (range: − 0.07 – -0.05). Overall, the size of the loop and the extent of *τ*^2^ did not appear to affect the results notably. Nevertheless, under inconsistency, MB for IF sunk approximately to -2 in all strategies regardless of the scenario.

#### P-score for each intervention

Contrary to moderate MOD, P-score of the new intervention (P-score-N) was markedly underestimated in all strategies – but more profoundly in ‘uncertainty interval’ – for large MOD (Supplementary Figure 2, Additional file 5). Underestimation of P-score-N was more considerable under consistency than inconsistency but mitigated for substantial *τ*^2^. However, in inconsistent networks with moderate MOD and substantial *τ*^2^, P-score-N was overestimated.

P-score of old intervention (P-score-O) was underestimated in all strategies for all scenarios, yet more profoundly for large MOD and/or present inconsistency (Supplementary Figure 3, Additional file 5). For large MOD, ‘uncertainty interval’ exerted comparatively lower MB for P-score-O. Overall, substantial *τ*^2^ or a larger loop led to slightly larger negative MB for P-score-O. On the contrary, MB for P-score for placebo was positive in all strategies for all scenarios and became particularly substantial for large MOD irrespectively the presence or absence of inconsistency (Supplementary Figure 4, Additional file 5). The extent of *τ*^2^ and loop size did not implicate the results overall.

### 95% coverage probability

**Fig. 5:**
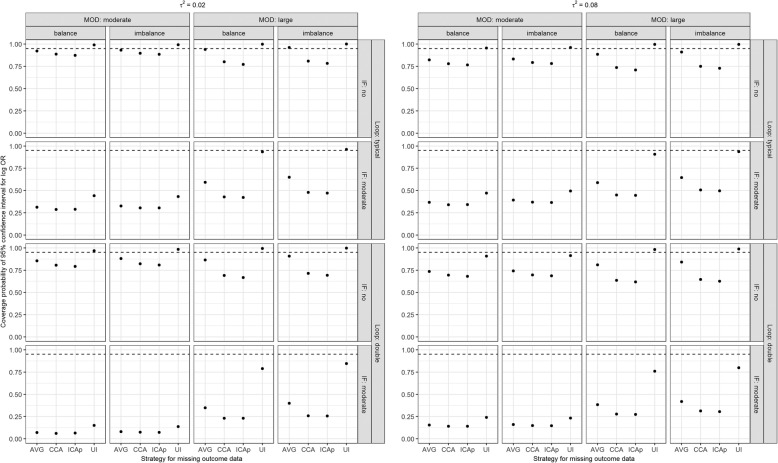
Coverage probability of 95% confidence interval for log OR (new versus old intervention) under informative MOD while accounting for the scenarios on the number of studies (typical loop, double), the extent of MOD (moderate, large), balance of MOD (balance, imbalance), the extent of between-trial variance (*τ*^2^; 0.02 as small, 0.08 as substantial), and extent of inconsistency (absent, moderate). AVG, on average MAR; CCA, complete case analysis; ICAp, imputed case analysis of observed event risks; IF, inconsistency factor; MOD, missing outcome data; UI, uncertainty interval

As expected, the coverage probability for log OR was below its nominal level for CCA and ICAp in all scenarios (Fig. 5). In the presence of consistency and small *τ*^2^, regardless of MOD extent, or substantial *τ*^2^ and large MOD, ‘uncertainty interval’ led to coverage probability for log OR above its nominal level, but it decreased as inconsistency regulated the network. Nevertheless, using ‘uncertainty interval’, coverage probability for log OR reached its nominal level in a typical loop with consistency, moderate MOD and substantial *τ*^2^, as well as in a typical loop with present inconsistency, large MOD and small *τ*^2^. In general, the coverage probability for log OR using ‘on average MAR’ was found somewhere in-between; however, it approached its nominal level only in a typical loop with present consistency and small *τ*^2^. Overall, all strategies underperformed when, in addition to inconsistency, MOD were moderate, or loop became larger. In general, results on the coverage probability for IF were in line with those on the coverage probability for log OR (Supplementary Figure 5, Additional file 5).

### Mean width of 95% confidence interval

**Fig. 6:**
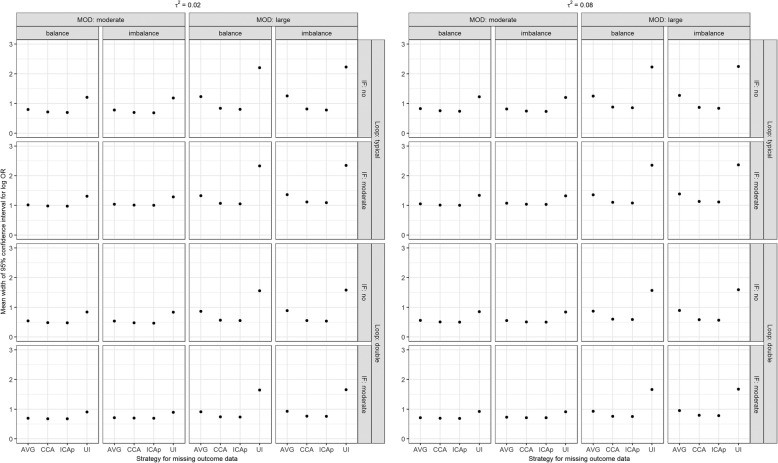
Mean width of 95% confidence interval for log OR (new versus old intervention) under informative MOD while accounting for the scenarios on the number of studies (typical loop, double), the extent of MOD (moderate, large), the balance of MOD (balance, imbalance), the extent of between-trial variance (*τ*^2^; 0.02 as small, 0.08 as substantial), and extent of inconsistency (absent, moderate). AVG, on average MAR; CCA, complete case analysis; ICAp, imputed case analysis of observed event risks; IF, inconsistency factor; MOD, missing outcome data; UI, uncertainty interval

In all scenarios, ‘uncertainty interval’ provided the widest confidence interval for log OR, followed by ‘on average MAR’, whereas CCA and ICAp had similar mean width of the confidence interval for log OR (Fig. 6). When the loop became larger, the mean width of the confidence interval for log OR reduced in all strategies, but it slightly increased in the presence of inconsistency. The extent of *τ*^2^ did not seem to implicate the results. Overall, results on the mean width of the confidence interval for IF were in line with those on the mean width of the confidence interval for log OR (Supplementary Figure 6, Additional file 5).

## Discussion

The present study is the first to investigate the performance of core NMA estimates using four different strategies to address MOD under MAR assumption within a frequentist NMA framework. We used our previous collection of networks from several health-related fields to perform the empirical study and to define the simulation scenarios [33]. We classified the strategies to those modelling (‘on average MAR’ – the reference strategy in our study) versus excluding (CCA and ‘uncertainty interval’) or imputing MOD (ICAp) and to those accounting for (‘on average MAR’ and ‘uncertainty interval’) versus ignoring uncertainty about MAR (CCA and ICAp).

Our empirical study indicated that ‘on average MAR’ agreed overall with CCA and ICAp in terms of log ORs, IFs and P-scores but it led to comparatively larger standard errors of log ORs and IFs under the latter two, especially for moderate and large MOD. Agreement between ‘on average MAR’ and ‘uncertainty interval’ was quite poor overall regarding the standard errors of log ORs and IFs, as they were systematically larger under the latter. By increasing the prior variance of log IMOR to 4 (the maximum allowed value to prevent inaccurate standard error of within-trial log ORs according to White et al. [8]), the agreement between ‘on average MAR’ and ‘uncertainty interval’ improved slightly for all NMA estimates (Supplementary Figure 27, Additional file 5). A good agreement between these two strategies could be achieved for a prior variance of log IMOR above 4, but then the statistical properties of log OR (and IF consequently) would be compromised [8]. It can be, therefore, concluded that ‘uncertainty interval’ leads unnecessarily to excessively large standard errors for log OR and IF and thus, to overly conservative inferences.

The simulation study confirmed the agreement of ‘on average MAR’ with CCA and ICAp in terms of log OR and IF, regardless of the scenario; however, their performance was compromised to a similar extent when MOD was large or unbalanced and inconsistency regulated the network as a consequence of underweighting further studies with large or unbalanced MOD – the sample size is reduced substantially and/or unbalanced and event rate is distorted – which, in conjunction with inconsistency in the network, affects the estimation of *τ*^2^ and by extent, NMA log OR and IF. As also revealed by the simulation study of Gamble and Hollis [13] for meta-analysis log OR, ‘uncertainty interval’ led to the least precise estimation of log OR (and IF as indicated by the large width of confidence intervals), especially in a typical loop with large MOD. Overall, contrary to other scenarios, a larger loop with moderate, balanced MOD, consistent evidence and small *τ*^2^ secured good statistical properties for the NMA estimates, since more (and relatively homogeneous) information was available, such as the number of studies and observed outcome data. As expected, low MOD ensured broad agreement among the strategies for all frequentist measures (Supplementary Figure 17–26, Additional file 5).

As indicated by the empirical study and the mean width of confidence intervals, CCA and ICAp provided more precise estimates of log OR and IF as opposed to ‘on average MAR’ and ‘uncertainty interval’; however, the former two yielded comparatively larger *τ*^2^. A possible explanation may be that the latter strategies assign a comparatively lower weight to trials with MOD, and hence, provide more imprecise within-trial log ORs [8, 10, 13] which result in the reduction of *τ*^2^ [8]. In principle, the trade-off between the precision loss in log ORs and reduced *τ*^2^ intensifies as MOD increase. However, since the estimated *τ*^2^ captures the extent of both *τ*^2^ and IF, and because different strategies quantify *τ*^2^ differently – while also considering the extent of MOD – the estimation of *τ*^2^ was substantially implicated in all strategies and for all scenarios. Having substantial *τ*^2^ and consistent evidence, underestimated *τ*^2^ in all strategies but more profoundly when the uncertainty due to MOD was considered. Since the DerSimonian and Laird estimator was used, truly substantial heterogeneity was inevitably underestimated [47] (in our empirical study, zero *τ*^2^ was estimated in 17, 21, 31, and 69% of the networks using ICAp, CCA, ‘on average MAR’, and ‘uncertainty interval’, respectively), especially for strategies that account for the uncertainty due to MOD as they mitigate statistical heterogeneity in essence by inflating within-trial standard errors. Nevertheless, having inconsistency in conjunction with substantial *τ*^2^, overestimated *τ*^2^ under CCA and ICAp but underestimated *τ*^2^ further using ‘uncertainty interval’. Only when evidence was consistent with small *τ*^2^ and moderate MOD, had different strategies little impact on the estimation of *τ*^2^.

When ‘uncertainty interval’ was used, *netmeta* gave warnings for the multi-arm trials in four networks: within-trial standard errors were inconsistent in some multi-arm trials in two networks [48, 49], whereas treatment-arm variances were negative in some multi-arm trials in another two networks [50, 51]. After using a tolerance threshold of 0.02, the problem disappeared only in one network [49]; however, a new warning appeared, as one of the ‘problematic’ multi-arm trials provided negative treatment variances. To preserve these networks in our analyses while tackling the warnings, we decided to reduce each ‘problematic’ multi-arm trial to a two-arm trial, while ensuring that this amendment would not affect the connectivity of the corresponding networks.

The strategies evaluated in the present work have been proposed for aggregate binary MOD. Mavridis et al. [52] have proposed a two-stage pattern-mixture model (similar to the ‘on average MAR’ strategy) to handle aggregate continuous MOD in a pairwise and network meta-analysis. To our knowledge, we are not aware of any published method to address time-to-event MOD and ordinal MOD in a series of trials. Furthermore, apart from the ‘on average MAR’ strategy (section “Modelling MOD using a two-stage pattern-mixture model”), all other strategies can be applied only under the MAR assumption. To indicate non-MAR assumptions using the two-stage pattern-mixture model (section “Modelling MOD using a two-stage pattern-mixture model”), we should set *Δ*_*i*, *k*_ ≠ 0 in Eq. (4). Ideally, *Δ*_*i*, *k*_ should be informed by clinical expert opinion tailored to the outcome and comparison type [7]. Turner et al. [7], and White et al. [9] discuss elicitation approaches that use an expert opinion on defining the degree of deviation from the MAR assumption as a sensitivity analysis in a series of trials. Nevertheless, extensive elicitation studies are needed to inform the missingness parameters properly in a pairwise and network meta-analysis.

In the present study, we have applied the ‘on average MAR’ strategy without accounting for important effect modifiers. To account also for important effect modifiers while avoiding ecological bias, it would require that we have access to individual patient data and enough trials to allow for effect-modification adjustments in a multiple imputation framework. Provided that both pre-requisites are fulfilled, then multiple imputation that also allows for missing not at random assumptions may offer more flexibility and also improve the results van Buuren et al. [53] developed a multiple imputation model that incorporates a delta parameter like IMOR under pattern-mixture model to investigate the degree of departure from MAR in survival analysis in a clinical trial. However, multiple imputation is currently not the norm in pairwise and network meta-analysis.

Major shortcomings of the present study mainly stem from the reporting quality of the collected networks and the implementation of a two-stage approach to address MOD. The extraction quality of the analysed networks was overall suboptimal since the reviewers failed to provide any information on the outcome of completers and the strategy applied to handle MOD [3, 54]. An inaccurate extraction may seriously compromise the validity of the NMA results, which, by extent, may hinder the true comparative performance of different strategies for MOD [54].

One limitation for using the two-stage approach to address binary MOD is the need for applying an abstract continuity correction to address the zero-cell problem that may arise (we faced this problem in four networks). Continuity correction has been repeatedly criticised for being a suboptimal strategy as it may lead to biased results [28, 55]. Another limitation is the reliance on normality assumption where, in addition, the (actually estimated) within-trial standard errors are assumed known (hidden assumption two in [56]); an assumption that is rather hard to defend in a typical pairwise or network meta-analysis where large and many studies are not the norm to justify this approximation [21, 24]. Consequently, the inherent correlation between within-trial standard errors and log ORs is ignored which, furthermore, increases the risk to obtain biased pooled log ORs [56–58]. These limitations can be tackled using likelihood-based methods – especially, Bayesian analysis, which remains the most popular framework in NMA [19, 20] – as the exact likelihood of the binary outcome data is considered, and thus, both continuity correction and normality assumption are inherently avoided [56].

Lastly, while ‘on average MAR’ is the most proper strategy to address MOD, it does not allow the observed data to contribute to the estimation of log IMOR – while borrowing strength across the trials – so that the model can ‘learn’ about the missingness mechanism(s) [7]. This is because ‘on average MAR’ merely fixes the log ORs and standard errors to the assumed prior mean (equal 0) and variance for log IMOR. Consequently, ‘on average MAR’ considers log IMOR to be independent of observed and missing outcomes [7, 8]. Furthermore, this strategy allows only a few scenarios about the structure of log IMOR to be modelled, therefore, restricting the full spectrum of modelling possibilities that best align with the condition and interventions investigated [7, 8]. These limitations can be overcome easily through a one-stage pattern-mixture model that allows the model to ‘learn’ about the missingness mechanism(s) while using plausible prior structures for the missingness parameter (as proposed in Turner et al. [7] for a pairwise meta-analysis and extended in NMA by Spineli [33]).

## Conclusions

CCA and ICAp are simple to apply yet suboptimal strategies, as they take MAR assumption at face value, and they may result in misleading inferences, especially when MOD are large and/or unbalanced. Accountability of uncertainty due to MOD rendered ‘on average MAR’ and ‘uncertainty interval’ as better alternatives – at least conceptually – to address MOD under MAR. Nevertheless, being a refinement of CCA, ‘uncertainty interval’ shares the same shortcomings and induces unnecessary imprecision in the NMA estimates with implications for the inferences. Therefore, modelling MOD via a pattern-mixture model while assuming MAR as a starting point (i.e. ‘on average MAR’) should be preferred to exclusion and imputation [27] as it constitutes a more proper strategy to address MOD in a systematic review – although computationally less straightforward – because it maintains the randomised sample in each arm of every trial while allowing for possible assumptions to quantify the association between MOD and outcome (and uncertainty thereof) via log IMOR. Nevertheless, in the presence of large MOD alone or in conjunction with substantial *τ*^2^ and inconsistent evidence, NMA estimates under ‘on average MAR’ should be interpreted with caution because their statistical performance is compromised to some extent. In this case, a sensitivity analysis to selected plausible assumptions about log IMOR is highly recommended to frame the limitations in the interpretation of NMA results.

## Supplementary information

**Additional file 1.** Analysed dataset.

**Additional file 2.** R scripts for (i) contrast-level long format dataset & (ii) missing outcome data strategies.

**Additional file 3.** Supplementary tables for the empirical study.

**Additional file 4.** Code to generate triangle networks and analyse in frequentist network meta-analysis.

**Additional file 5.** Supplementary figures for the empirical and simulation study.

## Data Availability

The authors declare that all data supporting the findings of this study are available within the article and its supplementary information files.

## Abbreviations

CCA: Complete case analysis
DGM: Data generating model
ICAp: Imputed case analysis of observed event risks
IF: Inconsistency factor
IMOR: Informative missingness odds ratio
LoA: Limits of agreement
MAR: Missing at random
MB: Mean bias
MOD: Missing participant outcome data
NMA: Network meta-analysis
OR: Odds ratio
P-score-N: P-score for new intervention
P-score-O: P-score for old intervention
SUCRA: Surface under the cumulative ranking curve
