# Supplementary material for: Comparison of exclusion, imputation and modelling of missing binary outcome data in frequentist network meta-analysis
Source: BMC Med Res Methodol. 2020 Feb 28;20:48. doi: 10.1186/s12874-020-00929-9 (PMC7049189; doi:10.1186/s12874-020-00929-9)
Supplement: Supplementary file 2 — Additional file 2. R scripts for (i) contrast-level long format dataset & (ii) missing outcome data strategies. [file 12874_2020_929_MOESM2_ESM.docx]

**R scripts for (i) contrast-level long format dataset & (ii) missing outcome data strategies**

| 1. **Contrast-level Long Format Data-frame** |
| --- |

Function to turn an arm-level wide format (as provided in Additional file 1) into a contrast-level long format. Under the contrast-level long format, the obtained data-frame includes two columns for the pairwise comparisons to indicate the interventions compared (t_1_ and t_2_), two columns for the respective number of events (r_1_ and r_2_), the number of missing participants (m_1_ and m_2_), and total randomised (n_1_ and n_2_). Therefore, a two-arm trial occupies one row of the data-frame, whereas a multi-arm trial occupies as many rows as the number of possible comparisons observed:

**Table 1.** Contrast-level long format for the evidence base of network no 3 (Additional file 1)

| trial | r_1_ | r_2_ | m_1_ | m_2_ | n_1_ | n_2_ | t_1_ | t_2_ |
| --- | --- | --- | --- | --- | --- | --- | --- | --- |
| 1 | 8 | 4 | 0 | 1 | 8 | 8 | 2 | 7 |
| 2 | 78 | 61 | 19 | 27 | 142 | 139 | 2 | 7 |
| … | … | … | … | … | … | … | … | … |
| 9 | 111 | 72 | 20 | 37 | 213 | 201 | 5 | 7 |
| 9 | 122 | 72 | 10 | 37 | 209 | 201 | 6 | 7 |
| 9 | 111 | 122 | 20 | 10 | 213 | 209 | 5 | 6 |
| 10 | 23 | 33 | 68 | 69 | 168 | 181 | 2 | 7 |
| … | … | … | … | … | … | … | … | … |

Function in R to obtain a ‘contrast-level long format’ data-frame for a network. Copy and paste the function into a new R script.

| ## BEGIN  contrast.level.dataset <- function(r, m, n, t){    combi <- rep(NA, length(r[, 1]))  for(i in 1:length(r[, 1])){  combi[i] <- dim(combn(length(na.omit(t[i, ])), 2))[2]  }    total <- mod <- treat.b <- treat <- event <- list()  for(i in 1:length(r[, 1])){  if(length(na.omit(t[i, ])) > 1){  treat.b[[i]] <- expand.grid(treat1 = na.omit(t[i, ]), treat2 = na.omit(t[i, ]))  treat[[i]] <- subset(expand.grid(treat1 = na.omit(t[i, ]), treat2 = na.omit(t[i, ])), treat1 < treat2)  event[[i]] <- subset(expand.grid(na.omit(r[i, ]), na.omit(r[i, ])), treat.b[[i]][,"treat1"] < treat.b[[i]][,"treat2"])  mod[[i]] <- subset(expand.grid(na.omit(m[i, ]), na.omit(m[i, ])), treat.b[[i]][,"treat1"] < treat.b[[i]][,"treat2"])  total[[i]] <- subset(expand.grid(na.omit(n[i, ]), na.omit(n[i, ])), treat.b[[i]][,"treat1"] < treat.b[[i]][,"treat2"])  } else {  treat[[i]] <- expand.grid(t[i, 1], t[i, 2])  event[[i]] <- expand.grid(r[i, 1], r[i, 2])  mod[[i]] <- expand.grid(m[i, 1], m[i, 2])  total[[i]] <- expand.grid(n[i, 1], n[i, 2])  }  }  a <- rep(1:length(r[, 1]), combi)  b <- do.call(rbind,lapply(1:length(r[, 1]), function(i) event[[i]]))  c <- do.call(rbind,lapply(1:length(r[, 1]), function(i) mod[[i]]))  d <- do.call(rbind,lapply(1:length(r[, 1]), function(i) total[[i]]))  e <- do.call(rbind,lapply(1:length(r[, 1]), function(i) treat[[i]]))    (dataset.final <- data.frame(a, b, c, d, e))  colnames(dataset.final) <- c("id", "e1", "e2", "m1", "m2", "n1", "n2", "t1", "t2");dataset.final    return(dataset.final)  }  ## END |
| --- |

Then, the obtained data-frame can be used in the following strategies to obtain log odds ratios and standard errors in each trial of the network: (i) complete case analysis, (ii) uncertainty interval, (iii) on average missing at random (MAR), and (iv) imputed case analysis of observed event risks.

| 1. **Complete Case Analysis** |
| --- |

Function to obtain trial-specific log odds ratios and standard errors after excluding missing participants from each arm of every trial (Section 2.1.1) [1].

| ## BEGIN  CCA <- function(data){ # Data-frame with contrast-level long format    for(i in 1:length(data[, 1])){    # Add 0.5 continuity correction when there is at least on zero cell  if(data[i, 2] == 0 \|\| data[i, 3] == 0 \|\| data[i, 6] - data[i, 4] - data[i, 2] == 0 \|\| data[i, 7] - data[i, 5] - data[i, 3] == 0){  data[i, 2] <- data[i, 2] + 0.5  data[i, 3] <- data[i, 3] + 0.5  data[i, 6] <- data[i, 6] + 1  data[i, 7] <- data[i, 7] + 1    } else {  data[i, 2] <- data[i, 2]  data[i, 3] <- data[i, 3]  data[i, 6] <- data[i, 6]  data[i, 7] <- data[i, 7]  }  }    # Calculate the probability of event among completers in arm 1 and 2  p.o1 <- data[, 2]/(data[, 6] - data[, 4]); p.o2 <- data[, 3]/(data[, 7] - data[, 5])    # Estimates the odds ratio in the logarithmic scale  logOR <- log(p.o1/(1 - p.o1)) - log(p.o2/(1 - p.o2))    # Estimates the standard error of log odds ratio  SElogOR <- sqrt( (1/((data[, 6] - data[, 4])*p.o1*(1 - p.o1))) + (1/((data[, 7] - data[, 5])*p.o2*(1 - p.o2))) )    # Include trial-specific adjusted logORs and SEs in the initial dataset  final <- data.frame(cbind(data[,], round(logOR, 4), round(SElogOR, 4)))  colnames(final) <- c("id", "e1", "e2", "m1", "m2", "n1", "n2", "t1", "t2", "logOR", "SElogOR")    return(final)  }  ## END |
| --- |

| 1. **Uncertainty Interval** |
| --- |

Function to obtain trial-specific log odds ratios and standard errors after applying the ‘uncertainty interval’ strategy in each trial (Section 2.1.2) [2].

| ## BEGIN  UncertaintyInterval <- function(data){ # Data-frame with contrast-level long format    for(i in 1:length(data[, 1])){    # Add 0.5 continuity correction when there is at least on zero cell  if(data[i, 2] == 0 \|\| data[i, 3] == 0 \|\| data[i, 6] - data[i, 4] - data[i, 2] == 0 \|\| data[i, 7] - data[i, 5] - data[i, 3] == 0){  data[i, 2] <- data[i, 2] + 0.5  data[i, 3] <- data[i, 3] + 0.5  data[i, 6] <- data[i, 6] + 1  data[i, 7] <- data[i, 7] + 1    } else {  data[i, 2] <- data[i, 2]  data[i, 3] <- data[i, 3]  data[i, 6] <- data[i, 6]  data[i, 7] <- data[i, 7]  }  }    p.full1 <- p.full2 <- p.obs1 <- p.obs2 <- p.best1 <- p.best2 <- p.worst1 <- p.worst2 <- numeric(length(data[, 1]))  logOR <- logOR.best <- logOR.worst <- SElogOR <- SElogOR.best <- SElogOR.worst <- numeric(length(data[, 1]))  lower.best <- upper.best <- lower.worst <- upper.worst <- lower.GH <- upper.GH <- numeric(length(data[, 1]))  for(i in 1:length(data[, 1])){  if(data[i, 4] == 0 & data[i, 5] == 0){    ## Full data (no MOD)  # Calculate the probability of event among completers in arm 1 and 2  p.full1[i] <- data[i, 2]/data[i, 6]; p.full2[i] <- data[i, 3]/data[i, 7]  # Calculate the logORs  logOR[i] <- log(p.full1[i]/(1 - p.full1[i])) - log(p.full2[i]/(1 - p.full2[i]))  SElogOR[i] <- sqrt((1/data[i, 2]) + (1/data[i, 3]) + (1/(data[i, 6] - data[i, 2])) + (1/(data[i, 7] - data[i, 3])))    } else {    ## Fixed MAR  # Calculate the probability of observed events in each arm of every trial  p.obs1[i] <- data[i, 2]/(data[i, 6] - data[i, 4]); p.obs2[i] <- data[i, 3]/(data[i, 7] - data[i, 5])  # Calculate the trial-specific logOR  logOR[i] <- log(p.obs1[i]/(1 - p.obs1[i])) - log(p.obs2[i]/(1 - p.obs2[i]))    ## Best-case scenario  # Calculate the probability of events under BC scenario in each arm of every trial  p.best1[i] <- (data[i, 2] + data[i, 4])/data[i, 6]; p.best2[i] <- data[i, 3]/data[i, 7]  # Calculate the trial-specific logOR, SE and bounds of 95% CI  logOR.best[i] <- log(p.best1[i]/(1 - p.best1[i])) - log(p.best2[i]/(1 - p.best2[i]))  SElogOR.best[i] <- sqrt((1/(data[i, 2] + data[i, 4])) + (1/data[i, 3]) + (1/(data[i, 6] - data[i, 4] - data[i, 2])) + (1/((data[i, 7] - data[i, 3]) + data[i, 5])))  lower.best[i] <- logOR.best[i] - 1.96*SElogOR.best[i]; upper.best[i] <- logOR.best[i] + 1.96*SElogOR.best[i]    ## Worst-case scenario  # Calculate the probability of event under WC scenario in each arm of every trial  p.worst1[i] <- data[i, 2]/data[i, 6]; p.worst2[i] <- (data[i, 3] + data[i, 5])/data[i, 7]  # Calculate the trial-specific logOR, SE and bounds of 95% C  logOR.worst[i] <- log(p.worst1[i]/(1 - p.worst1[i])) - log(p.worst2[i]/(1 - p.worst2[i]))  SElogOR.worst[i] <- sqrt((1/data[i, 2]) + (1/(data[i, 3] + data[i, 5])) + (1/((data[i, 6] - data[i, 2]) + data[i, 4])) + (1/(data[i, 7] - data[i, 5] - data[i, 3])))  lower.worst[i] <- logOR.worst[i] - 1.96*SElogOR.worst[i]; upper.worst[i] <- logOR.worst[i] + 1.96*SElogOR.worst[i]    ## Define lower and upper bound of 95% CI in each trial using the Gamble-Hollis approach  lower.GH[i] <- apply(cbind(lower.best[i], upper.best[i], lower.worst[i], upper.worst[i]), 1, min)  upper.GH[i] <- apply(cbind(lower.best[i], upper.best[i], lower.worst[i], upper.worst[i]), 1, max)    ## Calculate the trial-specific SE under the Gamble-Hollis approach  SElogOR[i] <- (upper.GH[i] - lower.GH[i])/3.92  }  }    # Include trial-specific adjusted logORs and SEs in the initial dataset  complete <- cbind(round(logOR, 3), round(SElogOR, 3))  colnames(complete) <- c("logOR", "SElogOR")    final <- cbind(data, complete)  return(final)  }  ## END |
| --- |

| 1. **On Average MAR (Two-stage Pattern-mixture Model)** |
| --- |

Function to obtain trial-specific log odds ratios and standard errors after applying the two-stage pattern-mixture model in each arm of every trial (Section 2.1.3) [3]. The arguments delta1 and delta2 refer to the prior mean value of the informative missingness odds ratio (IMOR) parameter in the logarithmic scale for experimental and control intervention, respectively, whereas var.delta1 and var.delta2 refer to the corresponding prior variance value of log IMOR. The argument rho indicates the correlated log IMORs across arms. Note that in our study, we used delta1 = delta2 = 0 to reflect the MAR assumption on average, var.delta1 = var.delta2 = 1 and rho = 0 to indicate uncorrelated log IMORs.

| ## BEGIN  Taylor.IMOR <- function(data, delta1, delta2, var.delta1, var.delta2, rho){    for(i in 1:length(data[, 1])){ # Data-frame with contrast-level long format    # Add 0.5 continuity correction when there is at least on zero cell  if(data[i, 2] == 0 \|\| data[i, 3] == 0 \|\| data[i, 6] - data[i, 4] - data[i, 2] == 0 \|\| data[i, 7] - data[i, 5] - data[i, 3] == 0){  data[i, 2] <- data[i, 2] + 0.5  data[i, 3] <- data[i, 3] + 0.5  data[i, 6] <- data[i, 6] + 1  data[i, 7] <- data[i, 7] + 1    } else {  data[i, 2] <- data[i, 2]  data[i, 3] <- data[i, 3]  data[i, 6] <- data[i, 6]  data[i, 7] <- data[i, 7]  }  }    # Calculate the probability of event among completers in arm 1 and 2  p.o1 <- data[, 2]/(data[, 6] - data[, 4]); p.o2 <- data[, 3]/(data[, 7] - data[, 5])    # Calculate the probability of missing outcome data in arm 1 and 2  a1 <- data[, 4]/data[, 6]; a2 <- data[, 5]/data[, 7]    # Calculate the probability of event in randomised sample in arm 1 and 2 via pattern-mixture model  p.all1 <- (1 - a1)*p.o1 + a1*( (exp(delta1)*p.o1)/(exp(delta1)*p.o1 + 1 - p.o1) ); p.all2 <- (1 - a2)*p.o2 + a2*( (exp(delta2)*p.o2)/(exp(delta2)*p.o2 + 1 - p.o2) )    # Estimates the odds ratio in the logarithmic scale  logOR <- log(p.all1/(1 - p.all1)) - log(p.all2/(1 - p.all2))    #################################  ## USE OF TAYLOR APPROXIMATION ##  #################################    # Derivative of p.all by p.o per arm (first term in Equation 14 in PMID: 17703496)  A1 <- 1 - a1 + (a1*exp(delta1))/(exp(delta1)*p.o1 + 1 - p.o1)^2; A2 <- 1 - a2 + (a2*exp(delta2))/(exp(delta2)*p.o2 + 1 - p.o2)^2    # Variance of p.o per arm (second term in Equation 14 in PMID: 17703496)  B1 <- (p.o1*(1 - p.o1))/(data[, 6] - data[, 4]); B2 <- (p.o2*(1 - p.o2))/(data[, 7] - data[, 5])    # Derivative of p.all by prob of MOD (i.e. a) per arm (third term in Equation 14 in PMID: 17703496)  C1 <- (p.o1*(1 - p.o1)*(exp(delta1) - 1))/(exp(delta1)*p.o1 + 1 - p.o1); C2 <- (p.o2*(1 - p.o2)*(exp(delta2) - 1))/(exp(delta2)*p.o2 + 1 - p.o2)    # Variance of prob of MOD (i.e. a) per arm (fourth term in Equation 14 in PMID: 17703496)  D1 <- (a1*(1 - a1))/data[, 6]; D2 <- (a2*(1 - a2))/data[, 7]    # Variance of log odds using delta-method per arm  E1 <- 1/(p.all1*(1 - p.all1)); E2 <- 1/(p.all2*(1 - p.all2))    # Derivative of p.all by delta per arm (second Equation after Equation (15) in PMID: 17703496)  H1 <- (a1*p.o1*(1 - p.o1)*exp(delta1))/(exp(delta1)*p.o1 + 1 - p.o1)^2; H2 <- (a2*p.o2*(1 - p.o2)*exp(delta2))/(exp(delta2)*p.o2 + 1 - p.o2)^2    # Variance using the observed cases (Equation 13 in PMID: 17703496)  v.obs <- (A1*A1*B1 + C1*C1*D1)*E1*E1 + (A2*A2*B2 + C2*C2*D2)*E2*E2    # Variance due to informative missingness (Equation 16 with correlation in PMID: 17703496)  v.delta <- H1*H1*var.delta1*E1*E1 + H2*H2*var.delta2*E2*E2 - 2*rho*H1*H2*sqrt(var.delta1)*sqrt(var.delta2)*E1*E2    # Variance using the randomised sample (Equation 10 in PMID: 17703496)  v.all <- v.obs + v.delta    # Include trial-specific adjusted logORs and SEs in the initial dataset  final <- data.frame(cbind(data, round(logOR, 3), round(sqrt(v.all), 3)))  colnames(final) <- c("id", "e1", "e2", "m1", "m2", "n1", "n2", "t1", "t2", "logOR", "SElogOR")    return(final)  }  ## END |
| --- |

| 1. **Imputed Case Analysis of Observed Event Risks** |
| --- |

Function to obtain trial-specific log odds ratios and standard errors after imputing the probability of event conditional on missing participants (i.e. p^m^ in equation (2)) with the probability of event conditional on completers (i.e. p^c^ in equation (2)) in each arm of every trial (Section 2.1.4) [1].

| ## BEGIN  ImputedMAR <- function(data){ # Data-frame with contrast-level long format    for(i in 1:length(data[, 1])){    # Add 0.5 continuity correction when there is at least on zero cell  if(data[i, 2] == 0 \|\| data[i, 3] == 0 \|\| data[i, 6] - data[i, 4] - data[i, 2] == 0 \|\| data[i, 7] - data[i, 5] - data[i, 3] == 0){  data[i, 2] <- data[i, 2] + 0.5  data[i, 3] <- data[i, 3] + 0.5  data[i, 6] <- data[i, 6] + 1  data[i, 7] <- data[i, 7] + 1    } else {  data[i, 2] <- data[i, 2]  data[i, 3] <- data[i, 3]  data[i, 6] <- data[i, 6]  data[i, 7] <- data[i, 7]  }  }    # Calculate the probability of event among completers in arm 1 and 2  p.o1 <- data[, 2]/(data[, 6] - data[, 4]); p.o2 <- data[, 3]/(data[, 7] - data[, 5])    # Calculate the probability of event in randomised sample in arm 1 and 2 via pattern-mixture model  p.all1 <- p.o1; p.all2 <- p.o2    # Estimates the odds ratio in the logarithmic scale  logOR <- log(p.all1/(1 - p.all1)) - log(p.all2/(1 - p.all2))    # Estimates the standard error of log odds ratio  SElogOR <- sqrt( (1/(data[, 6]*p.all1*(1 - p.all1))) + (1/(data[, 7]*p.all2*(1 - p.all2))) )    # Include trial-specific adjusted logORs and SEs in the initial dataset  final <- data.frame(cbind(data, round(logOR, 3), round(SElogOR, 3)))  colnames(final) <- c("id", "e1", "e2", "m1", "m2", "n1", "n2", "t1", "t2", "logOR", "SElogOR")    return(final)  }  ## END |
| --- |

Then, the output of functions B, C, D and E can be fed directly into the netmeta function of the R package netmeta [4] in order to perform frequentist network meta-analysis based on electrical networks and graph theory [5].

**References**

1. Higgins JP, White IR, Wood AM. Imputation methods for missing outcome data in meta-analysis of clinical trials. Clin Trials. 2008;5:225-39.
2. Gamble C, Hollis S. Uncertainty method improved on best-worst case analysis in a binary meta-analysis. J Clin Epidemiol. 2005;58:579-88.
3. White IR, Higgins JP, Wood AM. Allowing for uncertainty due to missing data in meta-analysis--part 1: two-stage methods. Stat Med. 2008;27:711-27.
4. Schwarzer G. netmeta: Network meta-analysis using frequentist methods. R package, version 0.9-7. 2015. URL: <https://github.com/guido-s/netmeta>.
5. Rücker G. Network meta-analysis, electrical networks and graph theory. Res Synth Methods. 2012;3(4):312–24.
