# Supplementary material for: Comparison of exclusion, imputation and modelling of missing binary outcome data in frequentist network meta-analysis
Source: BMC Med Res Methodol. 2020 Feb 28;20:48. doi: 10.1186/s12874-020-00929-9 (PMC7049189; doi:10.1186/s12874-020-00929-9)
Supplement: Supplementary file 3 — Additional file 3. Supplementary tables for the empirical study. [file 12874_2020_929_MOESM3_ESM.docx]

**Supplementary tables for the empirical study**

**Table S1.** Agreement on direction and strength of evidence and extent of heterogeneity

| **log odds ratio (reference-specific comparisons)** | | | | | | | | | | | | | | | | | |
| --- | --- | --- | --- | --- | --- | --- | --- | --- | --- | --- | --- | --- | --- | --- | --- | --- | --- |
|  | CCA | | | | | Uncertainty interval | | | | | ICAp | | | | |  |  |
| *Strength of evidence*^a^ | | | | | | | | | | | | | | | | | |
| Average | Weak | | | Strong | | Weak | | | Strong | | Weak | | | Strong | | | |
| Weak | 49% | | | 4% | | 51% | | | 1% | | 49% | | | 3% | | | |
| Strong | 1% | | | 47% | | 13% | | | 35% | | 3% | | | 45 | | | |
| Kappa | 0.91 (0.85, 0.97)* | | | | | 0.72 (0.62, 0.82) ^‡^ | | | | | 0.88 (0.81, 0.95)* | | | | | | |
| *Direction of evidence*^b^ | | | | | | | | | | | | | | | | | |
| Average | first | | | second | | first | | | second | | first | | | second | | |  |
| First | 85% | | | 0% | | 84% | | | 1% | | 85% | | | 0% | | |  |
| second | 1% | | | 15% | | 1% | | | 14% | | 1% | | | 15% | | |  |
| Kappa | 0.98 (0.94, 1.00)* | | | | | 0.94 (0.86 1.00)* | | | | | 0.98 (0.94, 1.00)* | | | | | |  |
| *Extent of between-trial variance*^c^ | | | | | | | | | | | | | | | | | |
| Average | LO | MO | | | LA | LO | MO | | | LA | LO | MO | | | LA | |  |
| Low | 59% | 10% | | | 3% | 72% | 0% | | | 0% | 59% | 10% | | | 3% | |  |
| Medium | 0% | 10% | | | 0% | 7% | 3% | | | 0% | 0% | 10% | | | 0% | |  |
| Large | 0% | 0% | | | 17% | 3% | 7% | | | 7% | 0% | 0% | | | 17% | |  |
| Kappa | 0.73 (0.49, 0.98) ^†^ | | | | | 0.54 (0.18, 0.91) ^†^ | | | | | 0.73 (0.49, 0.98) ^†^ | | | | | |  |
| **Inconsistency factor (log odds ratio)** | | | | | | | | | | | | | | | | | |
|  | CCA | | | | | Uncertainty interval | | | | | ICAp | | | | | |  |
| *Strength of evidence*^a^ | | | | | | | | | | | | | | | | | |
| Average | Weak | | Strong | | | Weak | | Strong | | | Weak | | Strong | | | |  |
| Weak | 97% | | 0% | | | 95% | | 2% | | | 97% | | 0% | | | |  |
| Strong | 1% | | 2% | | | 3% | | 0% | | | 1% | | 1% | | | |  |
| Kappa | 0.85 (0.57, 1.00) ^†^ | | | | | -0.02 (-0.76, 0.72) ^§^ | | | | | 0.66 (0.19, 1.00) ^†^ | | | | | |  |
| *Direction of evidence*^b^ | | | | | | | | | | | | | | | | | |
| Average | Positive | | Negative | | | Positive | | Negative | | | Positive | | Negative | | | |  |
| Positive | 47% | | 1% | | | 48% | | 0% | | | 45% | | 3% | | | |  |
| Negative | 1% | | 51% | | | 1% | | 51% | | | 1% | | 51% | | | |  |
| Kappa | 0.96 (0.92, 1.00)* | | | | | 0.97 (0.94, 1.00)* | | | | | 0.91 (0.84, 0.97)* | | | | | |  |

Average, on average missing at random; CCA, complete case analysis; ICAp, imputed case analysis of observed event risks; LA, large; LO, low; MO, moderate.

^a^Strong evidence when 0 (in the log scale) is not included in the 95% confidence interval, otherwise weak evidence.

^b^Whether the estimated log odds ratio favors the first or second intervention in a comparison. In the case of inconsistency factor, whether the difference between direct and indirect estimate for a specific comparison is positive or negative.

^c^Estimated between-trial variance is low, moderate and large when it is smaller than the median, between the median and 3^rd^ quartile and larger than the 3^rd^ quartile, respectively, of the selected predictive distribution for the true between-trial variance.

*Almost perfect agreement (0.81 – 1.00); ^‡^Substantial agreement (0.61 – 0.80); ^§^Poor agreement (< 0.00); ^†^95% confidence interval is too wide to judge the level of agreement with confidence.
