# Supplementary material for: Comparison of exclusion, imputation and modelling of missing binary outcome data in frequentist network meta-analysis
Source: BMC Med Res Methodol. 2020 Feb 28;20:48. doi: 10.1186/s12874-020-00929-9 (PMC7049189; doi:10.1186/s12874-020-00929-9)
Supplement: Supplementary file 4 — Additional file 4. Code to generate triangle networks and analyse in frequentist network meta-analysis. [file 12874_2020_929_MOESM4_ESM.docx]

**Code to generate triangle networks and analyse in frequentist network meta-analysis**

| **simulation.binmod.network: Generate triangle networks** |
| --- |

Simulate sim arm-level triangle networks (se, Additional file 1) for all possible scenarios about the type of loop (k), true odds ratio of old versus placebo and new versus placebo (mOP and mNP, respectively), true extent of between-trial variance and inconsistency factor (tau2 and IF, respectively), true prevalence and balance of missing outcome data within trial (pm and dm, respectively) and missingness mechanism as described by the magnitude of the informative missingness odds ratio parameter in the logarithmic scale (LOGIMOR). Load library truncnorm (truncated normal distribution) [1].

| simulation.binmod.network <- function(k, mOP, mNP, tau2, IF, pm, dm, LOGIMOR, sim){  options(warn = -1)    # Consistency equation to obtain TRUE odds ratio for the comparison of interest  if(IF == 0){  mNO <- exp(log(mNP) - log(mOP) + 0) # TRUE odds ratio on 'New vs Old' under no inconsistency  } else if(IF == 1){  mNO <- exp(log(mNP) - log(mOP) + 1) # TRUE odds ratio on 'New vs Old' under moderate inconsistency  }      # Set matrix of 'Initial' TRUE event risks in (E)xperimental- and (C)ontrol-arm  pE1.OP <- pC1.OP <- matrix(NA, nrow = sim, ncol = 4*k) # Old/Placebo in 'Old vs Placebo'  pE1.NP <- pC1.NP <- matrix(NA, nrow = sim, ncol = 3*k) # New/Placebo in 'New vs Placebo'  pE1.NO <- pC1.NO <- matrix(NA, nrow = sim, ncol = 1*k) # New/Old in 'New vs Old'      # Set matrix of 'Initial' TRUE logits in (E)xperimental- and (C)ontrol-arm  logitE1.OP <- logitC1.OP <- matrix(NA, nrow = sim, ncol = 4*k) # Old/Placebo in 'Old vs Placebo'  logitE1.NP <- logitC1.NP <- matrix(NA, nrow = sim, ncol = 3*k) # New/Placebo in 'New vs Placebo'  logitE1.NO <- logitC1.NO <- matrix(NA, nrow = sim, ncol = 1*k) # New/Old in 'New vs Old'      # Set matrix of TRUE logits (via 'Initial' TRUE logits) in (E)xperimental- and (C)ontrol-arm  logitE.OP <- logitC.OP <- matrix(NA, nrow = sim, ncol = 4*k) # Old/Placebo in 'Old vs Placebo'  logitE.NP <- logitC.NP <- matrix(NA, nrow = sim, ncol = 3*k) # New/Placebo in 'New vs Placebo'  logitE.NO <- logitC.NO <- matrix(NA, nrow = sim, ncol = 1*k) # New/Old in 'New vs Old'      # Set matrix of TRUE event risks (the back-calculated) in Experimental- and Control-arm  pE.OP <- pC.OP <- matrix(NA, nrow = sim, ncol = 4*k) # Old/Placebo in 'Old vs Placebo'  pE.NP <- pC.NP <- matrix(NA, nrow = sim, ncol = 3*k) # New/Placebo in 'New vs Placebo'  pE.NO <- pC.NO <- matrix(NA, nrow = sim, ncol = 1*k) # New/Old in 'New vs Old'      # Set matrix of OBSERVED event risks in Experimental- and Control-arm  poE.OP <- poC.OP <- matrix(NA, nrow = sim, ncol = 4*k) # Old/Placebo in 'Old vs Placebo'  poE.NP <- poC.NP <- matrix(NA, nrow = sim, ncol = 3*k) # New/Placebo in 'New vs Placebo'  poE.NO <- poC.NO <- matrix(NA, nrow = sim, ncol = 1*k) # New/Old in 'New vs Old'      # Set matrix of number of OBSERVED events in Experimental- and Control-arm  eventE.OP <- eventC.OP <- matrix(NA, nrow = sim, ncol = 4*k) # Old/Placebo in 'Old vs Placebo'  eventE.NP <- eventC.NP <- matrix(NA, nrow = sim, ncol = 3*k) # New/Placebo in 'New vs Placebo'  eventE.NO <- eventC.NO <- matrix(NA, nrow = sim, ncol = 1*k) # New/Old in 'New vs Old'      # Set matrix of logIMORs (informative missingness odds ratio) in Experimental- and Control-arm  deltaE.OP <- deltaC.OP <- matrix(NA, nrow = sim, ncol = 4*k) # Old/Placebo in 'Old vs Placebo'  deltaE.NP <- deltaC.NP <- matrix(NA, nrow = sim, ncol = 3*k) # New/Placebo in 'New vs Placebo'  deltaE.NO <- deltaC.NO <- matrix(NA, nrow = sim, ncol = 1*k) # New/Old in 'New vs Old'      # Set matrix with coding to indicate the interventions in each comparison  treatP.OP <- matrix(1, nrow = sim, ncol = 4*k) # Placebo in 'Old vs Placebo'  treatP.NP <- matrix(1, nrow = sim, ncol = 3*k) # Placebo in 'New vs Placebo'  treatO.NO <- matrix(2, nrow = sim, ncol = 1*k) # Old in 'New vs Old'  treatO.OP <- matrix(2, nrow = sim, ncol = 4*k) # Old in 'Old vs Placebo'  treatN.NP <- matrix(3, nrow = sim, ncol = 3*k) # New in 'New vs Placebo'  treatN.NO <- matrix(3, nrow = sim, ncol = 1*k) # New in 'New vs Old'      # Set matrix of number of arms in each trial (all two-arms)  id.OP <- matrix(NA, nrow = sim, ncol = 4*k) # 'Old vs Placebo'  id.NP <- matrix(NA, nrow = sim, ncol = 3*k) # 'New vs Placebo'  id.NO <- matrix(NA, nrow = sim, ncol = 1*k) # 'New vs Old'      # Set matrix of sample size in Experimental- and Control-arm  nE.OP <- nC.OP <- matrix(NA, nrow = sim, ncol = 4*k) # Old/Placebo in 'Old vs Placebo'  nE.NP <- nC.NP <- matrix(NA, nrow = sim, ncol = 3*k) # New/Placebo in 'New vs Placebo'  nE.NO <- nC.NO <- matrix(NA, nrow = sim, ncol = 1*k) # New/Old in 'New vs Old'      # Set matrix of number of missing outcome data (MOD) in Experimental- and Control-arm  mE.OP <- mC.OP <- matrix(NA, nrow = sim, ncol = 4*k) # Old/Placebo in 'Old vs Placebo'  mE.NP <- mC.NP <- matrix(NA, nrow = sim, ncol = 3*k) # New/Placebo in 'New vs Placebo'  mE.NO <- mC.NO <- matrix(NA, nrow = sim, ncol = 1*k) # New/Old in 'New vs Old'      # Set matrix of risk of MOD in Experimental- and Control-arm  pmE.OP <- pmC.OP <- matrix(NA, nrow = sim, ncol = 4*k) # Old/Placebo in 'Old vs Placebo'  pmE.NP <- pmC.NP <- matrix(NA, nrow = sim, ncol = 3*k) # New/Placebo in 'New vs Placebo'  pmE.NO <- pmC.NO <- matrix(NA, nrow = sim, ncol = 1*k) # New/Old in 'New vs Old'      # Set matrix of necessary quantities in the linking equation via IMOR in Experimental- and Control-arm (PMID: 25809313, Equations A.3, page 2075)  AE.OP <- AC.OP <- matrix(NA, nrow = sim, ncol = 4*k) # Old/Placebo in 'Old vs Placebo'  AE.NP <- AC.NP <- matrix(NA, nrow = sim, ncol = 3*k) # New/Placebo in 'New vs Placebo'  AE.NO <- AC.NO <- matrix(NA, nrow = sim, ncol = 1*k) # New/Old in 'New vs Old'  BE.OP <- BC.OP <- matrix(NA, nrow = sim, ncol = 4*k) # Old/Placebo in 'Old vs Placebo'  BE.NP <- BC.NP <- matrix(NA, nrow = sim, ncol = 3*k) # New/Placebo in 'New vs Placebo'  BE.NO <- BC.NO <- matrix(NA, nrow = sim, ncol = 1*k) # New/Old in 'New vs Old'      # Arrays of separate datasets: one for each direct comparison of the network  dataset.OP <- array(NA, dim = c(4*k, 9, sim)) # 'Old vs Placebo'  dataset.NP <- array(NA, dim = c(3*k, 9, sim)) # 'New vs Placebo'  dataset.NO <- array(NA, dim = c(1*k, 9, sim)) # 'NeW vs Old'      # List with ALL datasets to be used for NMA via GeMTC  arm.level.dataset <- array(0, dim = c(4*k + 3*k + 1*k, 9, sim), dimnames = list(NULL, c("id","r1","r2","m1","m2","n1","n2","t1","t2"), NULL))      for(i in 1:sim){  # Generate 'Initial' TRUE event risks in Control-am  pC1.OP[i, ] <- runif(4*k, 0.27, 0.40) # Placebo in 'Old vs Placebo'  pC1.NP[i, ] <- runif(3*k, 0.27, 0.40) # Placebo in 'New vs Placebo'  pC1.NO[i, ] <- runif(1*k, 0.63, 0.76) # Old in 'New vs Old'  # Obtain 'Initial' TRUE event risks in Experimental-arm  pE1.OP[i, ] <- (pC1.OP[i, ]*mOP)/(1 - pC1.OP[i, ] + pC1.OP[i, ]*mOP) # Old in 'Old vs Placebo'  pE1.NP[i, ] <- (pC1.NP[i, ]*mNP)/(1 - pC1.NP[i, ] + pC1.NP[i, ]*mNP) # New in 'New vs Placebo'  pE1.NO[i, ] <- (pC1.NO[i, ]*mNO)/(1 - pC1.NO[i, ] + pC1.NO[i, ]*mNO) # New in 'New vs Old'      # Obtain 'Initial' TRUE logitS in Experimental-arm  logitE1.OP[i, ] <- log(pE1.OP[i, ]/(1 - pE1.OP[i, ])) # Old in 'Old vs Placebo'  logitE1.NP[i, ] <- log(pE1.NP[i, ]/(1 - pE1.NP[i, ])) # New in 'New vs Placebo'  logitE1.NO[i, ] <- log(pE1.NO[i, ]/(1 - pE1.NO[i, ])) # New in 'New vs Old'  # Obtain 'Initial' TRUE logitS in Control-arm  logitC1.OP[i, ] <- log(pC1.OP[i, ]/(1 - pC1.OP[i, ])) # Placebo in 'Old vs Placebo'  logitC1.NP[i, ] <- log(pC1.NP[i, ]/(1 - pC1.NP[i, ])) # Placebo in 'New vs Placebo'  logitC1.NO[i, ] <- log(pC1.NO[i, ]/(1 - pC1.NO[i, ])) # Old in 'New vs Old'    ## Use predictive prior for 'all-cause mortality' for 'pharma vs. PBO/control' (Table IV in PMID: 25475839) - Low between-trial variance  if(tau2 == 0.02){  # Generate TRUE logits (via 'Initial' TRUE logitS) in Experimental-arm  logitE.OP[i, ] <- rnorm(4*k, logitE1.OP[i, ], sqrt(2*0.02/3)) # Old in 'Old vs Placebo'  logitE.NP[i, ] <- rnorm(3*k, logitE1.NP[i, ], sqrt(2*0.02/3)) # New in 'New vs Placebo'  logitE.NO[i, ] <- rnorm(1*k, logitE1.NO[i, ], sqrt(0.02/2)) # New in 'New vs Old'  # Generate TRUE logits (via 'Initial' TRUE logitS) in Generate Control-arm  logitC.OP[i, ] <- rnorm(4*k, logitC1.OP[i, ], sqrt(0.02/3)) # Placebo in 'Old vs Placebo'  logitC.NP[i, ] <- rnorm(3*k, logitC1.NP[i, ], sqrt(0.02/3)) # Placebo in 'New vs Placebo'  logitC.NO[i, ] <- rnorm(1*k, logitC1.NO[i, ], sqrt(0.02/2)) # Old in 'New vs Old'    ## Use predictive prior for 'generic healthcare setting' for 'pharma vs. PBO/control' (Table IV in PMID: 25475839) - Moderate between-trial variance  } else if(tau2 == 0.08){  # Generate TRUE logits (via 'Initial' TRUE logitS) in Experimental-arm  logitE.OP[i, ] <- rnorm(4*k, logitE1.OP[i, ], sqrt(2*0.08/3)) # Old in 'Old vs Placebo'  logitE.NP[i, ] <- rnorm(3*k, logitE1.NP[i, ], sqrt(2*0.08/3)) # New in 'New vs Placebo'  logitE.NO[i, ] <- rnorm(1*k, logitE1.NO[i, ], sqrt(0.08/2)) # New in 'New vs Old'  # Generate TRUE logits (via 'Initial' TRUE logitS) in Generate Control-arm  logitC.OP[i, ] <- rnorm(4*k, logitC1.OP[i, ], sqrt(0.08/3)) # Placebo in 'Old vs Placebo'  logitC.NP[i, ] <- rnorm(3*k, logitC1.NP[i, ], sqrt(0.08/3)) # Placebo in 'New vs Placebo'  logitC.NO[i, ] <- rnorm(1*k, logitC1.NO[i, ], sqrt(0.08/2)) # Old in 'New vs Old'  }      # Obtain 'back-calculated' TRUE event risks in Experimental-arm  pE.OP[i, ] <- 1/(1 + exp(-logitE.OP[i, ])) # Old in 'Old vs Placebo'  pE.NP[i, ] <- 1/(1 + exp(-logitE.NP[i, ])) # New in 'New vs Placebo'  pE.NO[i, ] <- 1/(1 + exp(-logitE.NO[i, ])) # New in 'New vs Old'  # Obtain 'back-calculated' TRUE event risks in Control-arm  pC.OP[i, ] <- 1/(1 + exp(-logitC.OP[i, ])) # Placebo in 'Old vs Placebo'  pC.NP[i, ] <- 1/(1 + exp(-logitC.NP[i, ])) # Placebo in 'New vs Placebo'  pC.NO[i, ] <- 1/(1 + exp(-logitC.NO[i, ])) # Old in 'New vs Old'      # Set matrix of number of arms in each trial (all two-arms)  id.OP[i, ] <- c(1:(4*k)) # 'Old vs Placebo'  id.NP[i, ] <- c(((4*k) + 1):((4*k) + (3*k))) # 'New vs Placebo'  id.NO[i, ] <- c(((4*k) + (3*k) + 1):((4*k) + (3*k) + (1*k))) # 'New vs Old'      # Generate Sample size in Experimental- and Control-arm (equal in both arms)  nE.OP[i, ] <- nC.OP[i, ] <- round(runif(4*k, 102, 187), 0) # Old/Placebo in 'Old vs Placebo'  nE.NP[i, ] <- nC.NP[i, ] <- round(runif(3*k, 102, 187), 0) # New/Placebo in 'New vs Placebo'  nE.NO[i, ] <- nC.NO[i, ] <- round(runif(1*k, 128, 241), 0) # New/Old in 'New vs Old'      ## Generate risk of MOD, while accounting for extent (low, moderate, large) and balance (balance or imbalance) of MOD  if(pm == "low" & dm == "balance" \|\| pm == "low" & dm == "imbalance"){  # Generate risk of MOD in Experimental- and Control-arm  pmE.OP[i, ] <- pmC.OP[i, ] <- runif(4*k, 0.00, 0.04) # Old/Placebo in 'Old vs Placebo'  pmE.NP[i, ] <- pmC.NP[i, ] <- runif(3*k, 0.00, 0.04) # New/Placebo in 'New vs Placebo'  pmE.NO[i, ] <- pmC.NO[i, ] <- runif(1*k, 0.00, 0.04) # New/Old in 'New vs Old'    } else if(pm == "moderate" & dm == "balance"){  # Generate risk of MOD in Experimental- and Control-arm  pmE.OP[i, ] <- pmC.OP[i, ] <- runif(4*k, 0.05, 0.20) # Old/Placebo in 'Old vs Placebo'  pmE.NP[i, ] <- pmC.NP[i, ] <- runif(3*k, 0.05, 0.20) # New/Placebo in 'New vs Placebo'  pmE.NO[i, ] <- pmC.NO[i, ] <- runif(1*k, 0.05, 0.20) # New/Old in 'New vs Old'    } else if(pm == "moderate" & dm == "imbalance"){  # Generate risk of MOD in Experimental-arm  pmE.OP[i, ] <- runif(4*k, 0.05, 0.10) # Old in 'Old vs Placebo'  pmE.NP[i, ] <- runif(3*k, 0.05, 0.10) # New in 'New vs Placebo'  pmE.NO[i, ] <- runif(1*k, 0.05, 0.10) # New in 'New vs Old'  # Generate risk of MOD in Control-arm  pmC.OP[i, ] <- runif(4*k, 0.11, 0.20) # Placebo in 'Old vs Placebo'  pmC.NP[i, ] <- runif(3*k, 0.11, 0.20) # Placebo in 'New vs Placebo'  pmC.NO[i, ] <- runif(1*k, 0.11, 0.20) # Old in 'New vs Old'    } else if(pm == "large" & dm == "balance"){  # Generate risk of MOD in Experimental- and Control-arm  pmE.OP[i, ] <- pmC.OP[i, ] <- runif(4*k, 0.21, 0.40) # Old/Placebo in 'Old vs Placebo'  pmE.NP[i, ] <- pmC.NP[i, ] <- runif(3*k, 0.21, 0.40) # New/Placebo in 'New vs Placebo'  pmE.NO[i, ] <- pmC.NO[i, ] <- runif(1*k, 0.21, 0.40) # New/Old in 'New vs Old'    } else if(pm == "large" & dm == "imbalance"){  # Generate risk of MOD in Experimental-arm  pmE.OP[i, ] <- runif(4*k, 0.21, 0.30) # Old in 'Old vs Placebo'  pmE.NP[i, ] <- runif(3*k, 0.21, 0.30) # New in 'New vs Placebo'  pmE.NO[i, ] <- runif(1*k, 0.21, 0.30) # New in 'New vs Old'  # Generate risk of MOD in Control-arm  pmC.OP[i, ] <- runif(4*k, 0.31, 0.40) # Placebo in 'Old vs Placebo'  pmC.NP[i, ] <- runif(3*k, 0.31, 0.40) # Placebo in 'New vs Placebo'  pmC.NO[i, ] <- runif(1*k, 0.31, 0.40) # Old in 'New vs Old'  }      # Generate number of MOD in Experimental-arm  mE.OP[i, ] <- rbinom(4*k, nE.OP[i, ], pmE.OP[i, ]) # Old in 'Old vs Placebo'  mE.NP[i, ] <- rbinom(3*k, nE.NP[i, ], pmE.NP[i, ]) # New in 'New vs Placebo'  mE.NO[i, ] <- rbinom(1*k, nE.NO[i, ], pmE.NO[i, ]) # New in 'New vs Old'  # Generate number of MOD in Control-arm  mC.OP[i, ] <- rbinom(4*k, nC.OP[i, ], pmC.OP[i, ]) # Placebo in 'Old vs Placebo'  mC.NP[i, ] <- rbinom(3*k, nC.NP[i, ], pmC.NP[i, ]) # Placebo in 'New vs Placebo'  mC.NO[i, ] <- rbinom(1*k, nC.NO[i, ], pmC.NO[i, ]) # Old in 'New vs Old'      # Generate logIMOR in Experimental-arm  if(LOGIMOR == "T"){  deltaE.OP[i, ] <- rtruncnorm(4*k, a = log(1), b = Inf, log(2), 1) # Old in 'Old vs Placebo'  deltaE.NP[i, ] <- rtruncnorm(3*k, a = log(1), b = Inf, log(2), 1) # New in 'New vs Placebo'  deltaE.NO[i, ] <- rtruncnorm(1*k, a = log(1), b = Inf, log(2), 1) # New in 'New vs Old'  } else {  deltaE.OP[i, ] <- rep(0.0001, 4*k) # Old in 'Old vs Placebo'  deltaE.NP[i, ] <- rep(0.0001, 3*k) # New in 'New vs Placebo'  deltaE.NO[i, ] <- rep(0.0001, 1*k) # New in 'New vs Old'  }  # Generate logIMOR in Control-arm  if(LOGIMOR == "T"){  deltaC.OP[i, ] <- rtruncnorm(4*k, a = -Inf, b = log(1), -log(2), 1) # Placebo in 'Old vs Placebo'  deltaC.NP[i, ] <- rtruncnorm(3*k, a = -Inf, b = log(1), -log(2), 1) # Placebo in 'New vs Placebo'  deltaC.NO[i, ] <- rtruncnorm(1*k, a = log(1), b = Inf, log(2), 1) # Old in 'New vs Old'  } else {  deltaC.OP[i, ] <- rep(0.0001, 4*k) # Placebo in 'Old vs Placebo'  deltaC.NP[i, ] <- rep(0.0001, 3*k) # Placebo in 'New vs Placebo'  deltaC.NO[i, ] <- rep(0.0001, 1*k) # Old in 'New vs Old'  }      # Obtain A component of linkage function (Experimental-arm) (PMID: 25809313, Equations A.3, page 2075)  AE.OP[i, ] <- (pmE.OP[i, ] - pE.OP[i, ])*(1 - exp(deltaE.OP[i, ])) - 1 # Old in 'Old vs Placebo'  AE.NP[i, ] <- (pmE.NP[i, ] - pE.NP[i, ])*(1 - exp(deltaE.NP[i, ])) - 1 # New in 'New vs Placebo'  AE.NO[i, ] <- (pmE.NO[i, ] - pE.NO[i, ])*(1 - exp(deltaE.NO[i, ])) - 1 # New in 'New vs Old'  # Obtain A component of linkage function (Control-arm)  AC.OP[i, ] <- (pmC.OP[i, ] - pC.OP[i, ])*(1 - exp(deltaC.OP[i, ])) - 1 # Placebo in 'Old vs Placebo'  AC.NP[i, ] <- (pmC.NP[i, ] - pC.NP[i, ])*(1 - exp(deltaC.NP[i, ])) - 1 # Placebo in 'New vs Placebo'  AC.NO[i, ] <- (pmC.NO[i, ] - pC.NO[i, ])*(1 - exp(deltaC.NO[i, ])) - 1 # Old in 'New vs Old'      # Obtain B component of linkage function (Experimental-arm) (PMID: 25809313, Equations A.3, page 2075)  BE.OP[i, ] <- 2*(1 - exp(deltaE.OP[i, ]))*(1 - pmE.OP[i, ]) # Old in 'Old vs Placebo'  BE.NP[i, ] <- 2*(1 - exp(deltaE.NP[i, ]))*(1 - pmE.NP[i, ]) # New in 'New vs Placebo'  BE.NO[i, ] <- 2*(1 - exp(deltaE.NO[i, ]))*(1 - pmE.NO[i, ]) # New in 'New vs Old'  # Obtain B component of linkage function (Control-arm)  BC.OP[i, ] <- 2*(1 - exp(deltaC.OP[i, ]))*(1 - pmC.OP[i, ]) # Placebo in 'Old vs Placebo'  BC.NP[i, ] <- 2*(1 - exp(deltaC.NP[i, ]))*(1 - pmC.NP[i, ]) # Placebo in 'New vs Placebo'  BC.NO[i, ] <- 2*(1 - exp(deltaC.NO[i, ]))*(1 - pmC.NO[i, ]) # Old in 'New vs Old'      # Obtain risk of observed events in Experimental-arm (PMID: 25809313, Equation A.4, page 2075)  poE.OP[i, ] <- max(0, min(1, (-AE.OP[i, ] - sqrt(AE.OP[i, ]*AE.OP[i, ] - 2*pE.OP[i, ]*BE.OP[i, ]))/BE.OP[i, ] )) # Old in 'Old vs Placebo'  poE.NP[i, ] <- max(0, min(1, (-AE.NP[i, ] - sqrt(AE.NP[i, ]*AE.NP[i, ] - 2*pE.NP[i, ]*BE.NP[i, ]))/BE.NP[i, ] )) # New in 'New vs Placebo'  poE.NO[i, ] <- max(0, min(1, (-AE.NO[i, ] - sqrt(AE.NO[i, ]*AE.NO[i, ] - 2*pE.NO[i, ]*BE.NO[i, ]))/BE.NO[i, ] )) # New in 'New vs Old'  # Obtain risk of observed events in Control-arm  poC.OP[i, ] <- max(0, min(1, (-AC.OP[i, ] - sqrt(AC.OP[i, ]*AC.OP[i, ] - 2*pC.OP[i, ]*BC.OP[i, ]))/BC.OP[i, ] )) # Placebo in 'Old vs Placebo'  poC.NP[i, ] <- max(0, min(1, (-AC.NP[i, ] - sqrt(AC.NP[i, ]*AC.NP[i, ] - 2*pC.NP[i, ]*BC.NP[i, ]))/BC.NP[i, ] )) # Placebo in 'New vs Placebo'  poC.NO[i, ] <- max(0, min(1, (-AC.NO[i, ] - sqrt(AC.NO[i, ]*AC.NO[i, ] - 2*pC.NO[i, ]*BC.NO[i, ]))/BC.NO[i, ] )) # Old in 'New vs Old'      # Generate number of observed events in Experimental-arm  eventE.OP[i, ] <- rbinom(4*k, nE.OP[i, ] - mE.OP[i, ], poE.OP[i, ]) # Old in 'Old vs Placebo'  eventE.NP[i, ] <- rbinom(3*k, nE.NP[i, ] - mE.NP[i, ], poE.NP[i, ]) # New in 'New vs Placebo'  eventE.NO[i, ] <- rbinom(1*k, nE.NO[i, ] - mE.NO[i, ], poE.NO[i, ]) # New in 'New vs Old'  # Generate number of observed events in Control-arm  eventC.OP[i, ] <- rbinom(4*k, nC.OP[i, ] - mC.OP[i, ], poC.OP[i, ]) # Placebo in 'Old vs Placebo'  eventC.NP[i, ] <- rbinom(3*k, nC.NP[i, ] - mC.NP[i, ], poC.NP[i, ]) # Placebo in 'New vs Placebo'  eventC.NO[i, ] <- rbinom(1*k, nC.NO[i, ] - mC.NO[i, ], poC.NO[i, ]) # Old in 'New vs Old'      # List of separate datasets: one for each direct comparison of the network  dataset.OP[,, i] <- cbind(id.OP[i, ], eventE.OP[i, ], eventC.OP[i, ], mE.OP[i, ], mC.OP[i, ],nE.OP[i, ], nC.OP[i, ], treatO.OP[i, ], treatP.OP[i, ]) # 'Old vs Placebo'  dataset.NP[,, i] <- cbind(id.NP[i, ], eventE.NP[i, ], eventC.NP[i, ], mE.NP[i, ], mC.NP[i, ],nE.NP[i, ], nC.NP[i, ], treatN.NP[i, ], treatP.NP[i, ]) # 'New vs Placebo'  dataset.NO[,, i] <- cbind(id.NO[i, ], eventE.NO[i, ], eventC.NO[i, ], mE.NO[i, ], mC.NO[i, ],nE.NO[i, ], nC.NO[i, ], treatN.NO[i, ], treatO.NO[i, ]) # 'New vs Old'      # Array with ALL datasets to be used in the GeMTC  arm.level.dataset[,, i] <- rbind(dataset.OP[,, i], dataset.NP[,, i], dataset.NO[,, i])  }  return(arm.level.dataset)  } |
| --- |

Description of scenarios considered for each important factor.

| sim <- 5000 # Number of simulations  mOP <- 1.5 # True odds ratio on 'Old vs Placebo'  mNP <- 2 # True odds ratio on 'New vs Placebo'  k <- c(1, 2) # Number of trials (k = 1 for typical loop; k = 2 for larger loop)  tau2 <- c(0.02, 0.08) # small and moderate between-trial variance equal to the median of the respective selected empirical distributions  IF <- c(0, 1) # Extent of inconsistency factor: no and moderate, respectively  pm <- c("low", "moderate", "large") # Extent of missing outcome data  dm <- c("balance", "imbalance") # Balance of missing outcome data within a trial  LOGIMOR <- c("T", "F") # Intervention-specific logIMOR or MAR (IMOR = 1)  compile <- expand.grid(list(k = k, tau2 = tau2, IF = IF, pm = pm, dm = dm, LOGIMOR = LOGIMOR)) |
| --- |

Collect the simulated triangle networks (arm-level dataset) for all scenarios

| mat <- list()  set.seed(123)  for(l in 1:length(compile[, 1])){ # The index 'l' corresponds to a specific scenario as a list and it contains all simulations as indicated by 'sim'.  mat[[l]] <- simulation.binmod.network(compile[l, 'k'], mOP, mNP, compile[l, 'tau2'], compile[l, 'IF'], compile[l, 'pm'], compile[l, 'dm'], compile[l, 'LOGIMOR'], sim)  } |
| --- |

Create a data-frame for each missing outcome data strategy (Section 2.1.1 – 2.1.4) to store the frequentist measures of performance for each scenario: complete case analysis (results.CCA), on average missing at random (results.average), uncertainty interval (results.UI) and imputed cases analysis of observed event risks (results.ICAp).

| results.CCA <- matrix(NA, length(k)*length(tau2)*length(IF)*length(pm)*length(dm)*length(LOGIMOR), 19)  results.CCA[, 1:6] <- as.matrix(expand.grid(k, tau2, IF, pm, dm, LOGIMOR)) # Create all posible combinations of scenarios  results.ICAp <- results.average <- results.UI <- results.CCA  colnames(results.ICAp) <- colnames(results.average) <- colnames(results.UI) <- colnames(results.CCA) <- c("n.studies", "tau2", "IF", "prob.miss", "miss.balance", "IMOR", "pscoreN", "pscoreO", "pscoreP","MB.mu", "MB.tau2", "MB.IF", "MB.pbest.N", "MB.pbest.O", "MB.pbest.P", "CP.mu", "CP.IF", "IW.mu", "IW.IF") |
| --- |

Generate true SUCRA (surface under the cumulative ranking) values [2] for each intervention while using the scenarios for (i) true odds ratios of the placebo-comparisons and (ii) true between-trial variance.

| set.seed(123)  B <- 10000  pbo <- matrix(0, nrow = B, ncol = length(tau2)) # 'Placebo versus Placebo'  old <- new <- pbo  rank <- array(0, dim = c(3, 3, length(tau2)))  hier <- list()  for(i in 1:length(tau2)){  old[, i] <- rnorm(B, log(mOP), sqrt(tau2[i])) # 'Old versus Placebo'  new[, i] <- rnorm(B, log(mNP), sqrt(tau2[i])) # 'New versus Placebo'  hier[[i]] <- data.frame(cbind(pbo[, i], old[, i], new[, i]), 3 + 1 - t(apply(cbind(pbo[, i], old[, i], new[, i]), 1, rank, ties.method = 'max'))) # For negative outcome remove 'nt + 1 -'  }  ## Obtain the rank probability for each rank and each intervention  colnames(rank) <- c("placebo", "old", "new")  for(i in 1:length(tau2)){  for(j in 1:3){  rank[j, , i] <- apply(hier[[i]][, 4:6], 2, function(x) sum(x == j))/B  }  }  (SUCRA.P <- apply(apply(rank[, 1, ], 2, cumsum)[1:2, ], 2, sum)/2) # SUCRA for PLACEBO  (SUCRA.O <- apply(apply(rank[, 2, ], 2, cumsum)[1:2, ], 2, sum)/2) # SUCRA for OLD  (SUCRA.N <- apply(apply(rank[, 3, ], 2, cumsum)[1:2, ], 2, sum)/2) # SUCRA for NEW  ## Store the true SUCRAs in the data-frame for each missing outcome data strategy (see, above)  (results.CCA[, 7] <- rep(rep(SUCRA.N, each = length(k)), length(results.CCA[, 1])/(length(k)*length(tau2))))  (results.CCA[, 8] <- rep(rep(SUCRA.O, each = length(k)), length(results.CCA[, 1])/(length(k)*length(tau2))))  (results.CCA[, 9] <- rep(rep(SUCRA.P, each = length(k)), length(results.CCA[, 1])/(length(k)*length(tau2))))  results.ICAp[, 7] <- results.average[, 7] <- results.UI[, 7] <- results.CCA[, 7]  results.ICAp[, 8] <- results.average[, 8] <- results.UI[, 8] <- results.CCA[, 8]  results.ICAp[, 9] <- results.average[, 9] <- results.UI[, 9] <- results.CCA[, 9] |
| --- |

Analyse the simulated triangle networks using frequentist network meta-analysis (NMA) based on electrical network theory to obtain the NMA estimates (i.e. log odds ratio of new versus old intervention, between-trial variance, inconsistency factor (for log odds ratio) and p-score for each intervention) and frequentist measures of performance (i.e. mean bias, coverage probability and width of confidence interval). Load library netmeta (Network Meta-Analysis using Frequentist Methods) [3] and the R scripts for the missing outcome data strategies (functions B – E, Additional file 2):

| binmod.freq.network.analysis <- function(dataset, k, tau2, IF, pm, dm, LOGIMOR, mNP, mOP, sim){    counter1 <- 0 # Returning the % of completed simulations  index <- 1    ###########################################################################################################  ### CREATE VECTOR FOR EACH STATISTIC TO STORE NMA Estimates FOR ALL SIMULATIONS AND FOR EACH STRATEGY ###  ###########################################################################################################      tempmat.ICAp <- tempmat.average <- tempmat.UI <- tempmat.CCA <- as.data.frame(matrix(NA, sim, 10))        for(i in 1:length(results.CCA[, 1])){ # LOOP For SCENARIOS    index.sim <- 1      for(l in 1:sim){ # LOOP FOR SIMULATED DATASET  sim.data <- as.data.frame(mat[[i]][,, l])        ## Run netmeta  (NMA.CCA <- netmeta(logOR, SElogOR, t1, t2, id, data = CCA(sim.data), comb.fixed = F, comb.random = T))  (NMA.UI <- netmeta(logOR, SElogOR, t1, t2, id, data = UncertaintyInterval(sim.data), comb.fixed = F, comb.random = T))  (NMA.average <- netmeta(logOR, SElogOR, t1, t2, id, data = Taylor.IMOR(sim.data, delta1 = 0, delta2 = 0, var.delta1 = 1, var.delta2 = 1, rho = 0), comb.fixed = F, comb.random = T))  (NMA.ICAp <- netmeta(logOR, SElogOR, t1, t2, id, data = ImputedMAR(sim.data), comb.fixed = F, comb.random = T))    #########################################################################################################################  ### COLLECT RESULTS ON (i) LOR & 95%CI, (ii) BETWEEN-TRIAL VARIANCE (iii) IF & 95% CrI (iv) PSCORES PER INTERVENTION ###  #########################################################################################################################      ## Collect estimated logORs on 'New vs Old' comparison and 95% CI  tempmat.CCA[l, 1] <- NMA.CCA$TE.random[3, 2]  tempmat.CCA[l, 2] <- NMA.CCA$lower.random[3, 2]  tempmat.CCA[l, 3] <- NMA.CCA$upper.random[3, 2]  tempmat.UI[l, 1] <- NMA.UI$TE.random[3, 2]  tempmat.UI[l, 2] <- NMA.UI$lower.random[3, 2]  tempmat.UI[l, 3] <- NMA.UI$upper.random[3, 2]  tempmat.average[l, 1] <- NMA.average$TE.random[3, 2]  tempmat.average[l, 2] <- NMA.average$lower.random[3, 2]  tempmat.average[l, 3] <- NMA.average$upper.random[3, 2]  tempmat.ICAp[l, 1] <- NMA.ICAp$TE.random[3, 2]  tempmat.ICAp[l, 2] <- NMA.ICAp$lower.random[3, 2]  tempmat.ICAp[l, 3] <- NMA.ICAp$upper.random[3, 2]    ## Collect estimated between-trial variance  tempmat.CCA[l, 4] <- (NMA.CCA$tau)^2  tempmat.UI[l, 4] <- (NMA.UI$tau)^2  tempmat.average[l, 4] <- (NMA.average$tau)^2  tempmat.ICAp[l, 4] <- (NMA.ICAp$tau)^2      ## Collect estimated IF and 95% CI  tempmat.CCA[l, 5] <- netsplit(NMA.CCA)$compare.random[3, 2]  tempmat.CCA[l, 6] <- netsplit(NMA.CCA)$compare.random[3, 4]  tempmat.CCA[l, 7] <- netsplit(NMA.CCA)$compare.random[3, 5]  tempmat.UI[l, 5] <- netsplit(NMA.UI)$compare.random[3, 2]  tempmat.UI[l, 6] <- netsplit(NMA.UI)$compare.random[3, 4]  tempmat.UI[l, 7] <- netsplit(NMA.UI)$compare.random[3, 5]  tempmat.average[l, 5] <- netsplit(NMA.average)$compare.random[3, 2]  tempmat.average[l, 6] <- netsplit(NMA.average)$compare.random[3, 4]  tempmat.average[l, 7] <- netsplit(NMA.average)$compare.random[3, 5]  tempmat.ICAp[l, 5] <- netsplit(NMA.ICAp)$compare.random[3, 2]  tempmat.ICAp[l, 6] <- netsplit(NMA.ICAp)$compare.random[3, 4]  tempmat.ICAp[l, 7] <- netsplit(NMA.ICAp)$compare.random[3, 5]      ## Collect estimated pscore for all interventions  tempmat.CCA[l, 8] <- netrank(NMA.CCA, small.values = "bad")$Pscore.random[1]  tempmat.CCA[l, 9] <- netrank(NMA.CCA, small.values = "bad")$Pscore.random[2]  tempmat.CCA[l, 10] <- netrank(NMA.CCA, small.values = "bad")$Pscore.random[3]  tempmat.UI[l, 8] <- netrank(NMA.UI, small.values = "bad")$Pscore.random[1]  tempmat.UI[l, 9] <- netrank(NMA.UI, small.values = "bad")$Pscore.random[2]  tempmat.UI[l, 10] <- netrank(NMA.UI, small.values = "bad")$Pscore.random[3]  tempmat.average[l, 8] <- netrank(NMA.average, small.values = "bad")$Pscore.random[1]  tempmat.average[l, 9] <- netrank(NMA.average, small.values = "bad")$Pscore.random[2]  tempmat.average[l, 10] <- netrank(NMA.average, small.values = "bad")$Pscore.random[3]  tempmat.ICAp[l, 8] <- netrank(NMA.ICAp, small.values = "bad")$Pscore.random[1]  tempmat.ICAp[l, 9] <- netrank(NMA.ICAp, small.values = "bad")$Pscore.random[2]  tempmat.ICAp[l, 10] <- netrank(NMA.ICAp, small.values = "bad")$Pscore.random[3]    index.sim <- index.sim + 1  counter1 <- counter1 + 1    print(paste("Percentage completed: ", round((counter1/(sim*length(k)*length(tau2)*length(IF)*length(pm)*length(dm)*length(LOGIMOR)))*100, 6),"%"))    } # END of SIMULATIONS LOOP    #################################################################################################################  ### COLLECT FREQUENTIST MEASURES OF PERFORMANCE: (i) MEAN BIAS (ii) COVERAGE PROBABILITY (iii) 95% CI WIDTH ###  #################################################################################################################      ## MEAN BIAS  # logOR  MB.mu.CCA <- sum(tempmat.CCA[, 1] - (log(mNP) - log(mOP) + as.numeric(results.CCA[index, 3])))/sim  MB.mu.UI <- sum(tempmat.UI[, 1] - (log(mNP) - log(mOP) + as.numeric(results.UI[index, 3])))/sim  MB.mu.average <- sum(tempmat.average[, 1] - (log(mNP) - log(mOP) + as.numeric(results.average[index, 3])))/sim  MB.mu.ICAp <- sum(tempmat.ICAp[, 1] - (log(mNP) - log(mOP) + as.numeric(results.ICAp[index, 3])))/sim  # tausq  MB.tau2.CCA <- sum(tempmat.CCA[, 4] - as.numeric(results.CCA[index, 2]))/sim  MB.tau2.UI <- sum(tempmat.UI[, 4] - as.numeric(results.UI[index, 2]))/sim  MB.tau2.average <- sum(tempmat.average[, 4] - as.numeric(results.average[index, 2]))/sim  MB.tau2.ICAp <- sum(tempmat.ICAp[, 4] - as.numeric(results.ICAp[index, 2]))/sim  # IF  MB.IF.CCA <- sum(tempmat.CCA[, 5] - as.numeric(results.CCA[index, 3]))/sim  MB.IF.UI <- sum(tempmat.UI[, 5] - as.numeric(results.UI[index, 3]))/sim  MB.IF.average <- sum(tempmat.average[, 5] - as.numeric(results.average[index, 3]))/sim  MB.IF.ICAp <- sum(tempmat.ICAp[, 5] - as.numeric(results.ICAp[index, 3]))/sim  # pscore NEW  MB.pbest.N.CCA <- sum(tempmat.CCA[, 10] - as.numeric(results.CCA[index, 7]))/sim  MB.pbest.N.UI <- sum(tempmat.UI[, 10] - as.numeric(results.UI[index, 7]))/sim  MB.pbest.N.average <- sum(tempmat.average[, 10] - as.numeric(results.average[index, 7]))/sim  MB.pbest.N.ICAp <- sum(tempmat.ICAp[, 10] - as.numeric(results.ICAp[index, 7]))/sim  # pscore OLD  MB.pbest.O.CCA <- sum(tempmat.CCA[, 9] - as.numeric(results.CCA[index, 8]))/sim  MB.pbest.O.UI <- sum(tempmat.UI[, 9] - as.numeric(results.UI[index, 8]))/sim  MB.pbest.O.average <- sum(tempmat.average[, 9] - as.numeric(results.average[index, 8]))/sim  MB.pbest.O.ICAp <- sum(tempmat.ICAp[, 9] - as.numeric(results.ICAp[index, 8]))/sim  # pbest PBO  MB.pbest.P.CCA <- sum(tempmat.CCA[, 8] - as.numeric(results.CCA[index, 9]))/sim  MB.pbest.P.UI <- sum(tempmat.UI[, 8] - as.numeric(results.UI[index, 9]))/sim  MB.pbest.P.average <- sum(tempmat.average[, 8] - as.numeric(results.average[index, 9]))/sim  MB.pbest.P.ICAp <- sum(tempmat.ICAp[, 8] - as.numeric(results.ICAp[index, 9]))/sim      ## COVERAGE PROBABILITY  # logOR  CP.mu.CCA <- sum(tempmat.CCA[, 2] <= (log(mNP) - log(mOP) + as.numeric(results.CCA[index, 3])) & tempmat.CCA[, 3] >= (log(mNP) - log(mOP) + as.numeric(results.CCA[index, 3])))/sim  CP.mu.UI <- sum(tempmat.UI[, 2] <= (log(mNP) - log(mOP) + as.numeric(results.UI[index, 3])) & tempmat.UI[, 3] >= (log(mNP) - log(mOP) + as.numeric(results.UI[index, 3])))/sim  CP.mu.average <- sum(tempmat.average[, 2] <= (log(mNP) - log(mOP) + as.numeric(results.average[index, 3])) & tempmat.average[, 3] >= (log(mNP) - log(mOP) + as.numeric(results.average[index, 3])))/sim  CP.mu.ICAp <- sum(tempmat.ICAp[, 2] <= (log(mNP) - log(mOP) + as.numeric(results.ICAp[index, 3])) & tempmat.ICAp[, 3] >= (log(mNP) - log(mOP) + as.numeric(results.ICAp[index, 3])))/sim  # IF  CP.IF.CCA <- sum(tempmat.CCA[, 6] <= as.numeric(results.CCA[index, 3]) & tempmat.CCA[, 7] >= as.numeric(results.CCA[index, 3]))/sim  CP.IF.UI <- sum(tempmat.UI[, 6] <= as.numeric(results.UI[index, 3]) & tempmat.UI[, 7] >= as.numeric(results.UI[index, 3]))/sim  CP.IF.average <- sum(tempmat.average[, 6] <= as.numeric(results.average[index, 3]) & tempmat.average[, 7] >= as.numeric(results.average[index, 3]))/sim  CP.IF.ICAp <- sum(tempmat.ICAp [, 6] <= as.numeric(results.ICAp[index, 3]) & tempmat.ICAp[, 7] >= as.numeric(results.ICAp[index, 3]))/sim    ## INTERVAL WIDTH  # logOR  IW.mu.CCA <- sum(tempmat.CCA[, 3] - tempmat.CCA[, 2])/sim  IW.mu.UI <- sum(tempmat.UI[, 3] - tempmat.UI[, 2])/sim  IW.mu.average <- sum(tempmat.average[, 3] - tempmat.average[, 2])/sim  IW.mu.ICAp <- sum(tempmat.ICAp[, 3] - tempmat.ICAp[, 2])/sim  # IF  IW.IF.CCA <- sum(tempmat.CCA[, 7] - tempmat.CCA[, 6])/sim  IW.IF.UI <- sum(tempmat.UI[, 7] - tempmat.UI[, 6])/sim  IW.IF.average <- sum(tempmat.average[, 7] - tempmat.average[, 6])/sim  IW.IF.ICAp <- sum(tempmat.ICAp[, 7] - tempmat.ICAp[, 6])/sim    results.CCA[index, c(10:19)] <- c(MB.mu.CCA, MB.tau2.CCA, MB.IF.CCA, MB.pbest.N.CCA, MB.pbest.O.CCA, MB.pbest.P.CCA, CP.mu.CCA, CP.IF.CCA, IW.mu.CCA, IW.IF.CCA)  results.UI[index, c(10:19)] <- c(MB.mu.UI, MB.tau2.UI, MB.IF.UI, MB.pbest.N.UI, MB.pbest.O.UI, MB.pbest.P.UI, CP.mu.UI, CP.IF.UI, IW.mu.UI, IW.IF.UI)  results.average[index, c(10:19)] <- c(MB.mu.average, MB.tau2.average, MB.IF.average, MB.pbest.N.average, MB.pbest.O.average, MB.pbest.P.average, CP.mu.average, CP.IF.average, IW.mu.average, IW.IF.average)  results.ICAp[index, c(10:19)] <- c(MB.mu.ICAp, MB.tau2.ICAp, MB.IF.ICAp, MB.pbest.N.ICAp, MB.pbest.O.ICAp, MB.pbest.P.ICAp, CP.mu.ICAp, CP.IF.ICAp, IW.mu.ICAp, IW.IF.ICAp)  index <- index + 1    }  return(list(results.CCA, results.UI, results.average, results.ICAp))  } |
| --- |

Calculate time needed to complete the whole simulation-analysis procedure.

| start.time <- Sys.time()  (mtc.results <- binmod.freq.network.analysis(mat, k, tau2, IF, pm, dm, LOGIMOR, mNP, mOP, sim))  end.time <- Sys.time()  (time.taken <- end.time - start.time) |
| --- |

Export results as R.Data: save(mtc.results, file = "<your directory>/RESULTS.RData")

**References**

1. Mersmann O, Trautmann H, Steuer D, Bornkamp B. truncnorm: Density, probability, quantile and random number generation functions for the truncated normal distribution. R package, version 1.0-8. 2018. URL: <https://github.com/olafmersmann/truncnorm>.
2. Salanti G, Ades AE, Ioannidis JPA. Graphical methods and numerical summaries for presenting results from multiple-treatment meta-analysis: an overview and tutorial. J Clin Epidemiol. 2011;64:163–71.
3. Schwarzer G. netmeta: Network meta-analysis using frequentist methods. R package, version 0.9-7. 2015. <https://github.com/guido-s/netmeta>.
