# Supplementary material for: Comparison of exclusion, imputation and modelling of missing binary outcome data in frequentist network meta-analysis
Source: BMC Med Res Methodol. 2020 Feb 28;20:48. doi: 10.1186/s12874-020-00929-9 (PMC7049189; doi:10.1186/s12874-020-00929-9)
Supplement: Supplementary file 5 — Additional file 5. Supplementary figures for the empirical and simulation study. [file 12874_2020_929_MOESM5_ESM.docx]

**Supplementary figures for the empirical and simulation study**

| **Informative missing outcome data with moderate and large extent** |
| --- |

1. **Mean bias**

**
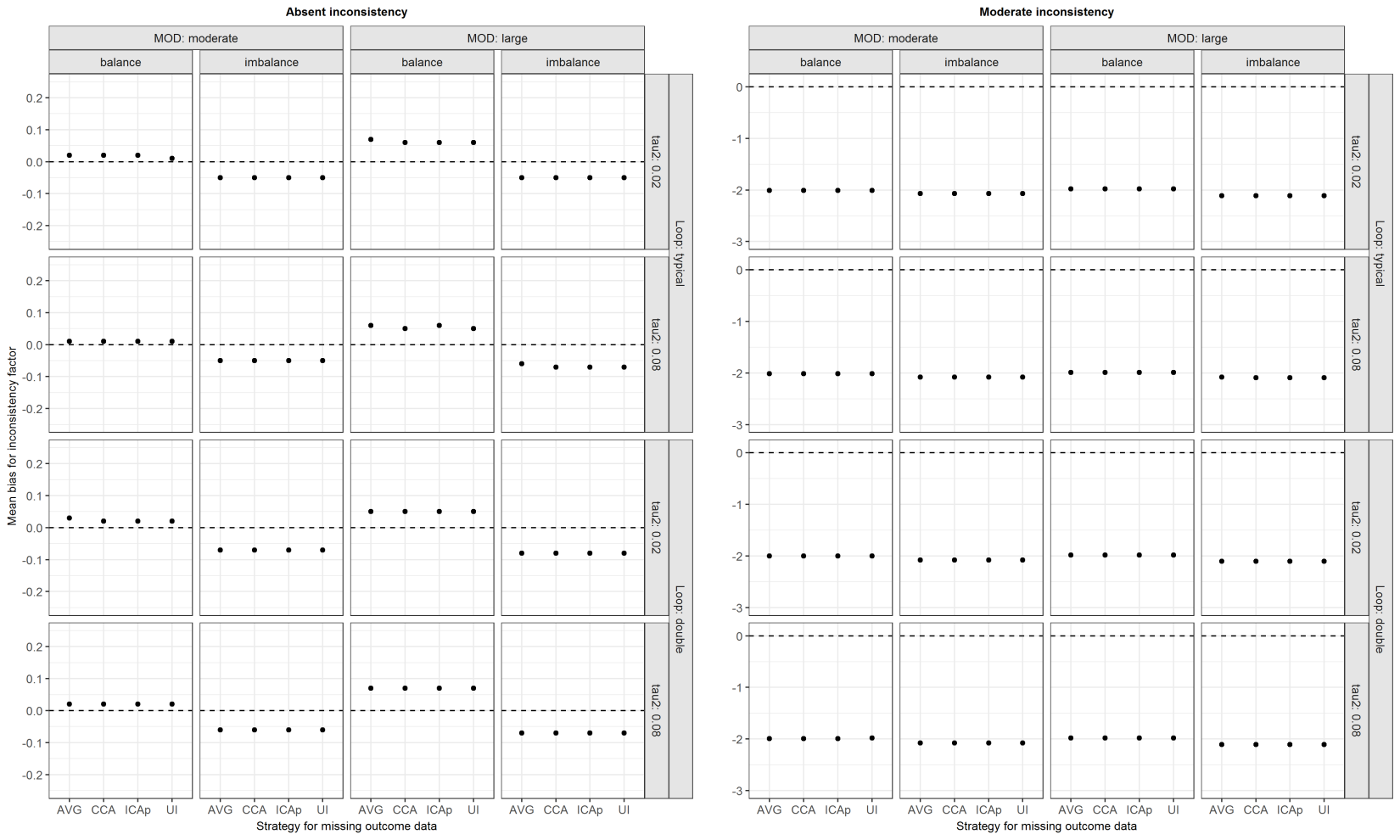
**

**Figure S1.** Mean bias for inconsistency factor (difference between direct and indirect evidence for the comparison of new and old intervention) under informative missingness while accounting for the number of studies (typical loop, double), extent of missing outcome data (moderate, large), balance of missing outcome data (balance, imbalance), and extent of between-trial variance ($\tau^{2}$; 0.02 as small, 0.08 as substantial). AVG, on average MAR; CCA, complete case analysis; ICAp, imputed case analysis of observed event risks; MOD, missing outcome data; UI, uncertainty interval.

**
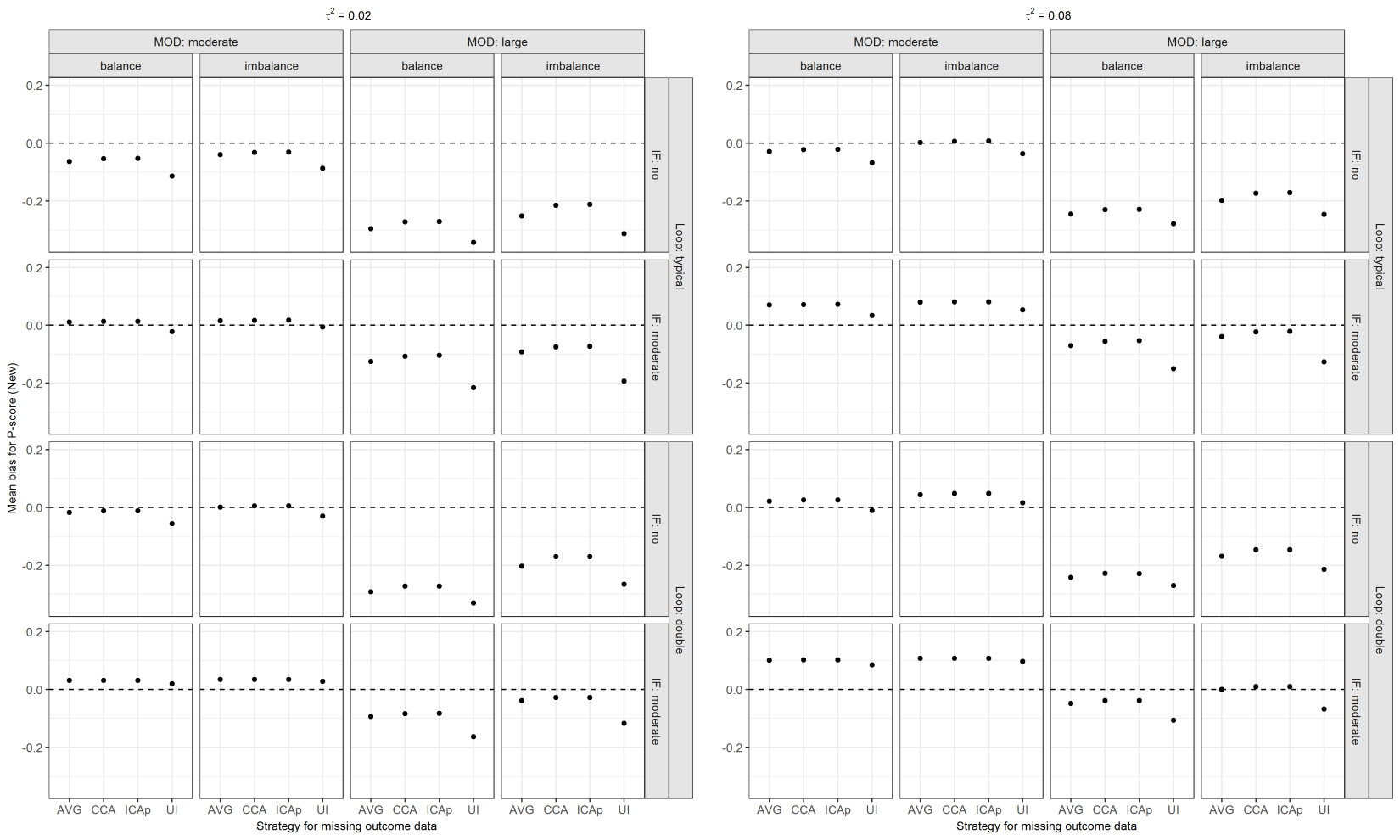
**

**Figure S2.** Mean bias for P-score of new intervention under informative missingness while accounting for the number of studies (typical loop, double), extent of missing outcome data (moderate, large), balance of missing outcome data (balance, imbalance), extent of between-trial variance ($\tau^{2}$; 0.02 as small, 0.08 as substantial), and extent of inconsistency (absent, moderate). AVG, on average MAR; CCA, complete case analysis; ICAp, imputed case analysis of observed event risks; IF, inconsistency factor; MOD, missing outcome data; UI, uncertainty interval.

**
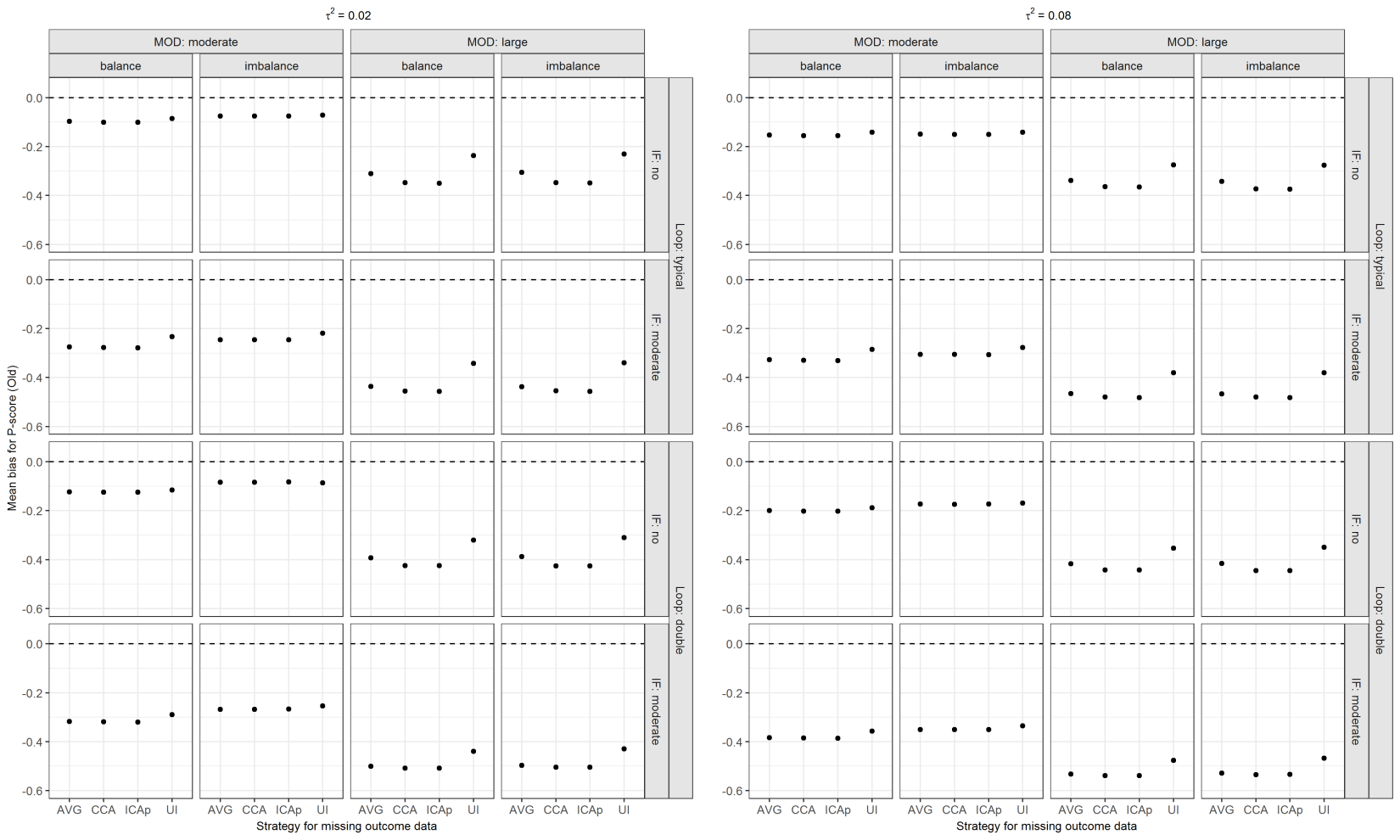
**

**Figure S3.** Mean bias for P-score of old intervention under informative missingness while accounting for the number of studies (typical loop, double), extent of missing outcome data (moderate, large), balance of missing outcome data (balance, imbalance), extent of between-trial variance ($\tau^{2}$; 0.02 as small, 0.08 as substantial), and extent of inconsistency (absent, moderate). AVG, on average MAR; CCA, complete case analysis; ICAp, imputed case analysis of observed event risks; IF, inconsistency factor; MOD, missing outcome data; UI, uncertainty interval.

**
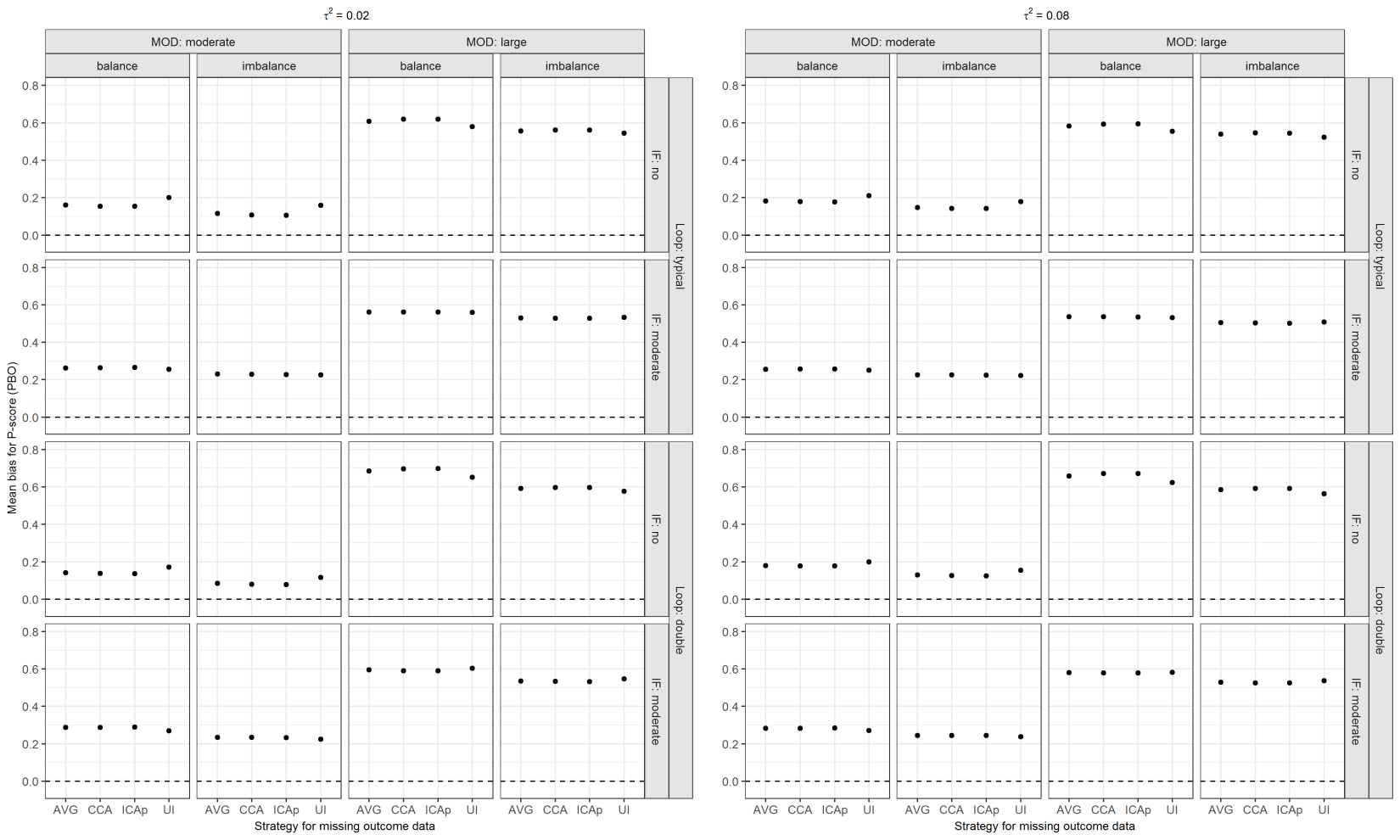
**

**Figure S4.** Mean bias for P-score of placebo under informative missingness while accounting for the number of studies (typical loop, double), extent of missing outcome data (moderate, large), balance of missing outcome data (balance, imbalance), extent of between-trial variance ($\tau^{2}$; 0.02 as small, 0.08 as substantial), and extent of inconsistency (absent, moderate). AVG, on average MAR; CCA, complete case analysis; ICAp, imputed case analysis of observed event risks; IF, inconsistency factor; MOD, missing outcome data; UI, uncertainty interval.

1. **Coverage probability of 95% confidence interval**

**
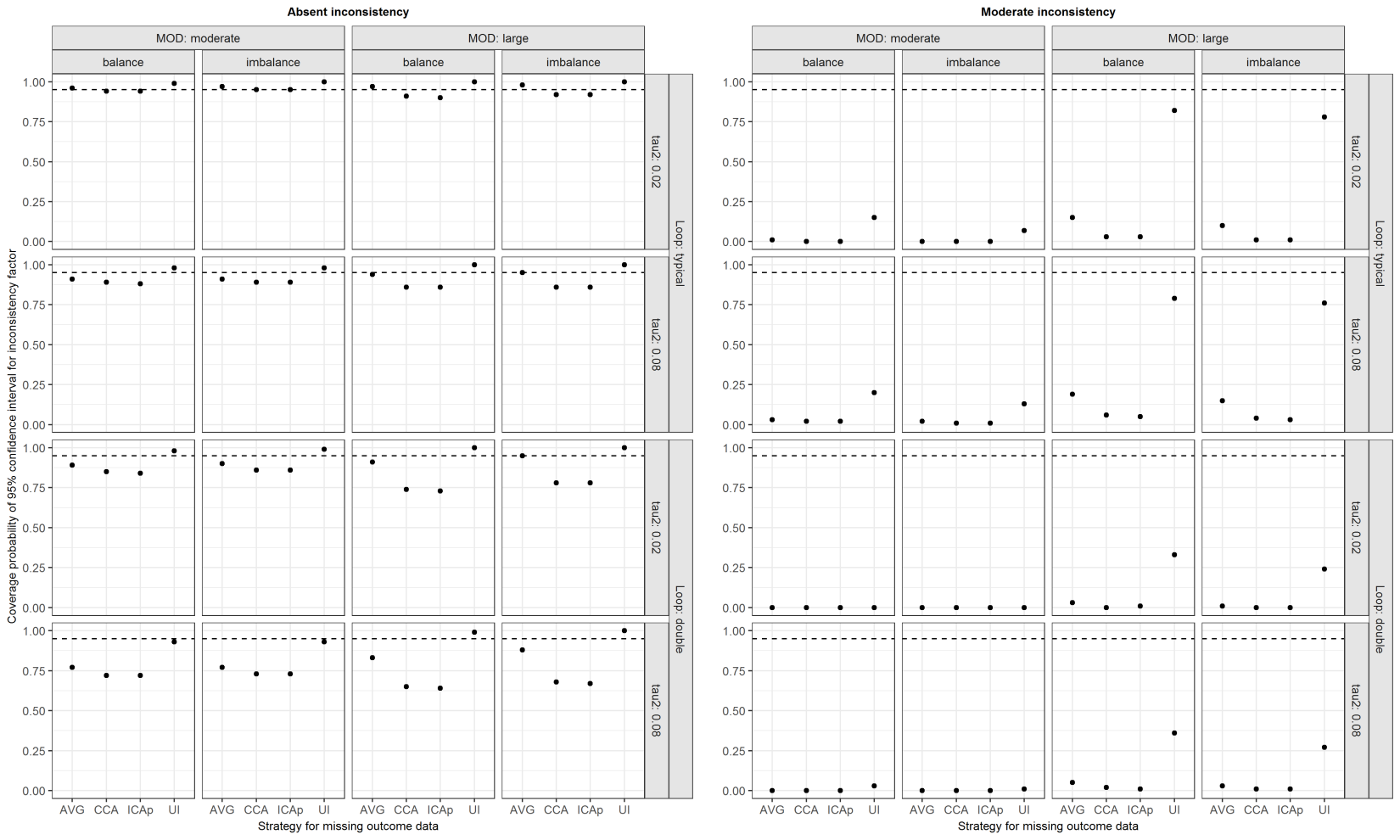
**

**Figure S5.** Coverage probability of 95% confidence interval for inconsistency factor (difference between direct and indirect evidence for the comparison between new and old intervention) under informative missingness while accounting for the number of studies (typical loop, double), extent of missing outcome data (moderate, large), balance of missing outcome data (balance, imbalance), and extent of between-trial variance ($\tau^{2}$; 0.02 as small, 0.08 as substantial). AVG, on average MAR; CCA, complete case analysis; ICAp, imputed case analysis of observed event risks; MOD, missing outcome data; UI, uncertainty interval.

1. **Mean width of 95% confidence interval**

**
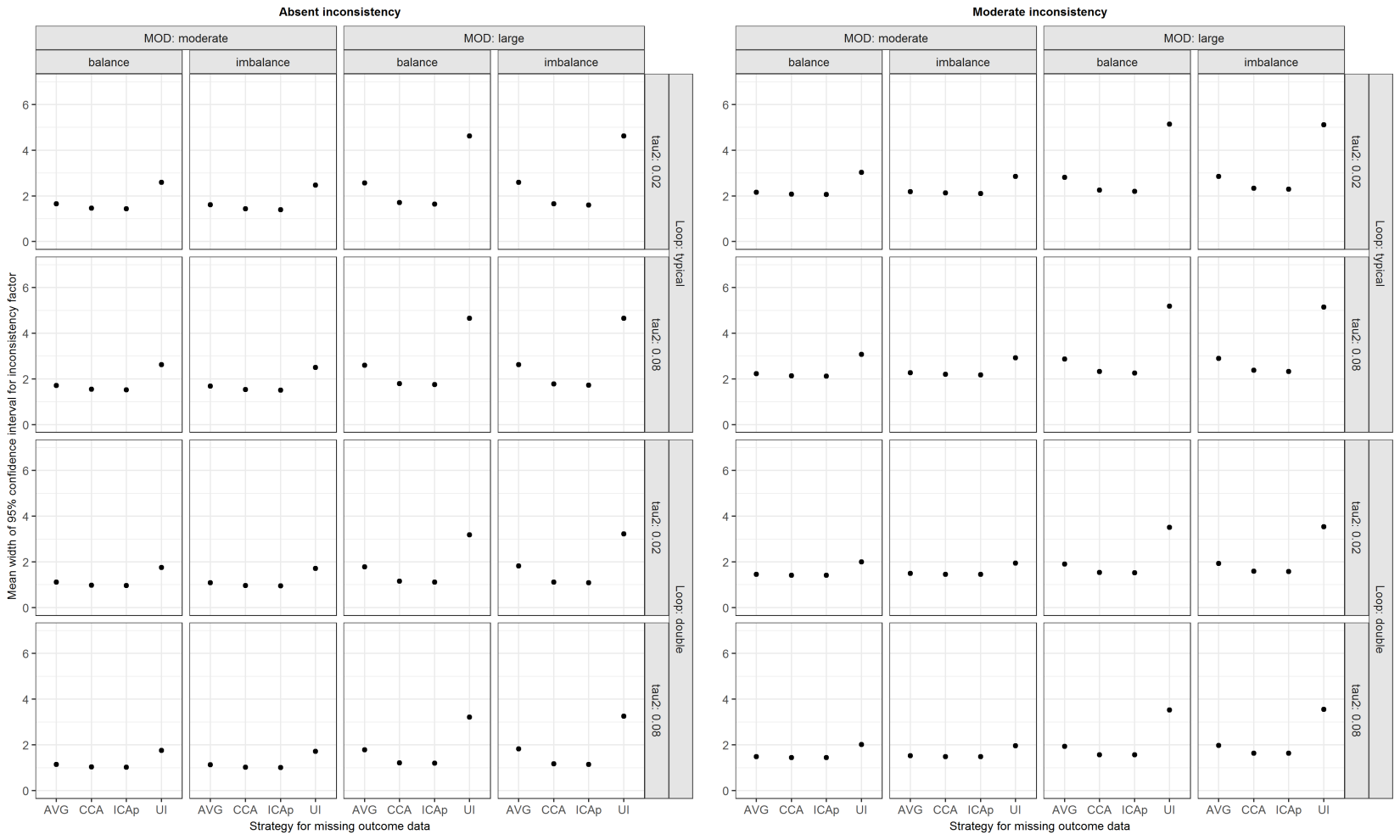
**

**Figure S6.** Mean width of 95% confidence interval for inconsistency factor (difference between direct and indirect evidence for the comparison between new and old intervention) under informative missingness while accounting for the number of studies (typical loop, double), extent of missing outcome data (moderate, large), balance of missing outcome data (balance, imbalance), and extent of between-trial variance ($\tau^{2}$; 0.02 as small, 0.08 as substantial). AVG, on average MAR; CCA, complete case analysis; ICAp, imputed case analysis of observed event risks; MOD, missing outcome data; UI, uncertainty interval.

| **Missing at random (MAR) with moderate and large missing outcome data** |
| --- |

1. **Mean bias**

**
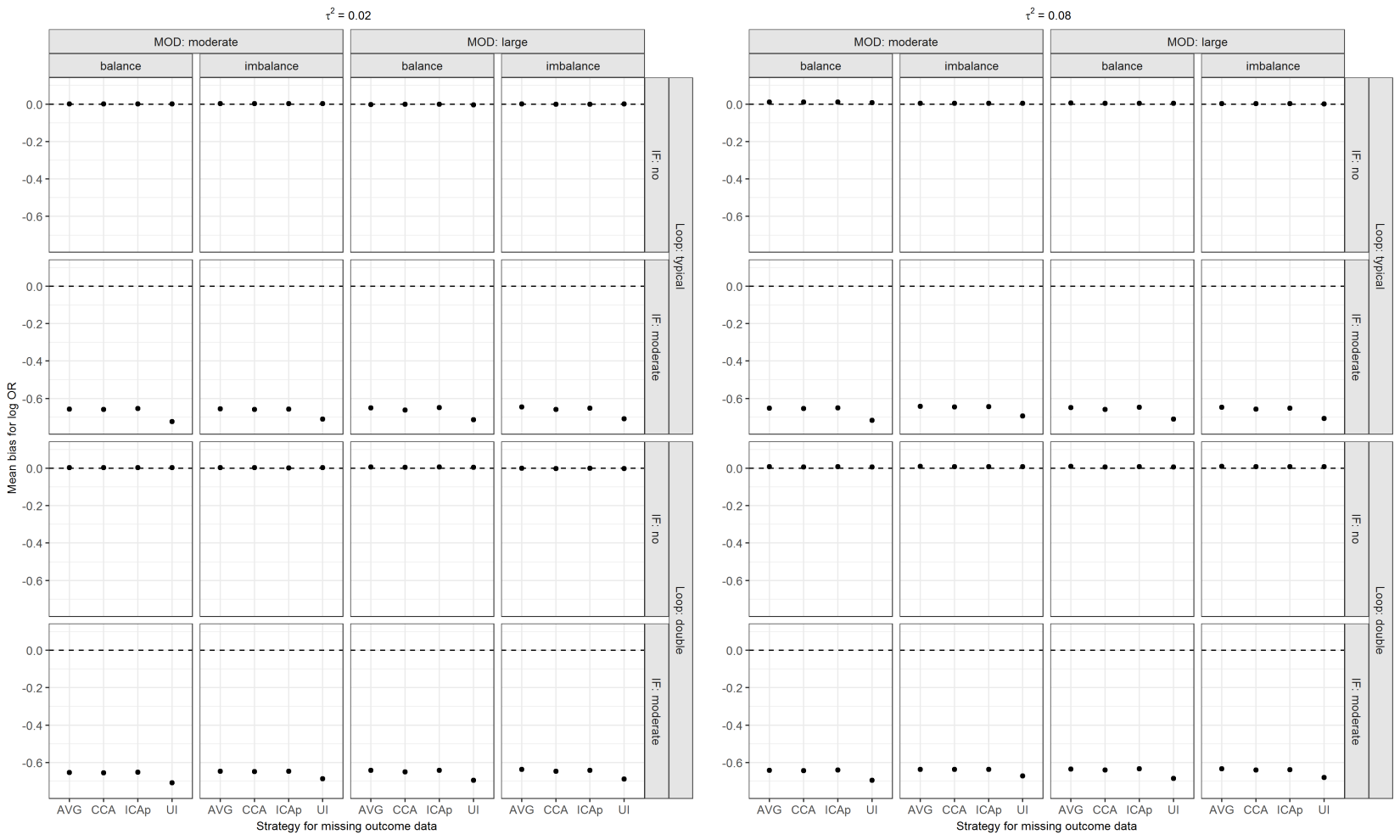
**

**Figure S7.** Mean bias for log OR (between new and old intervention) under MAR while accounting for the number of studies (typical loop, double), extent of missing outcome data (moderate, large), balance of missing outcome data (balance, imbalance), extent of between-trial variance ($\tau^{2}$; 0.02 as small, 0.08 as substantial), and extent of inconsistency (absent, moderate). AVG, on average MAR; CCA, complete case analysis; ICAp, imputed case analysis of observed event risks; IF, inconsistency factor; MOD, missing outcome data; UI, uncertainty interval.

**
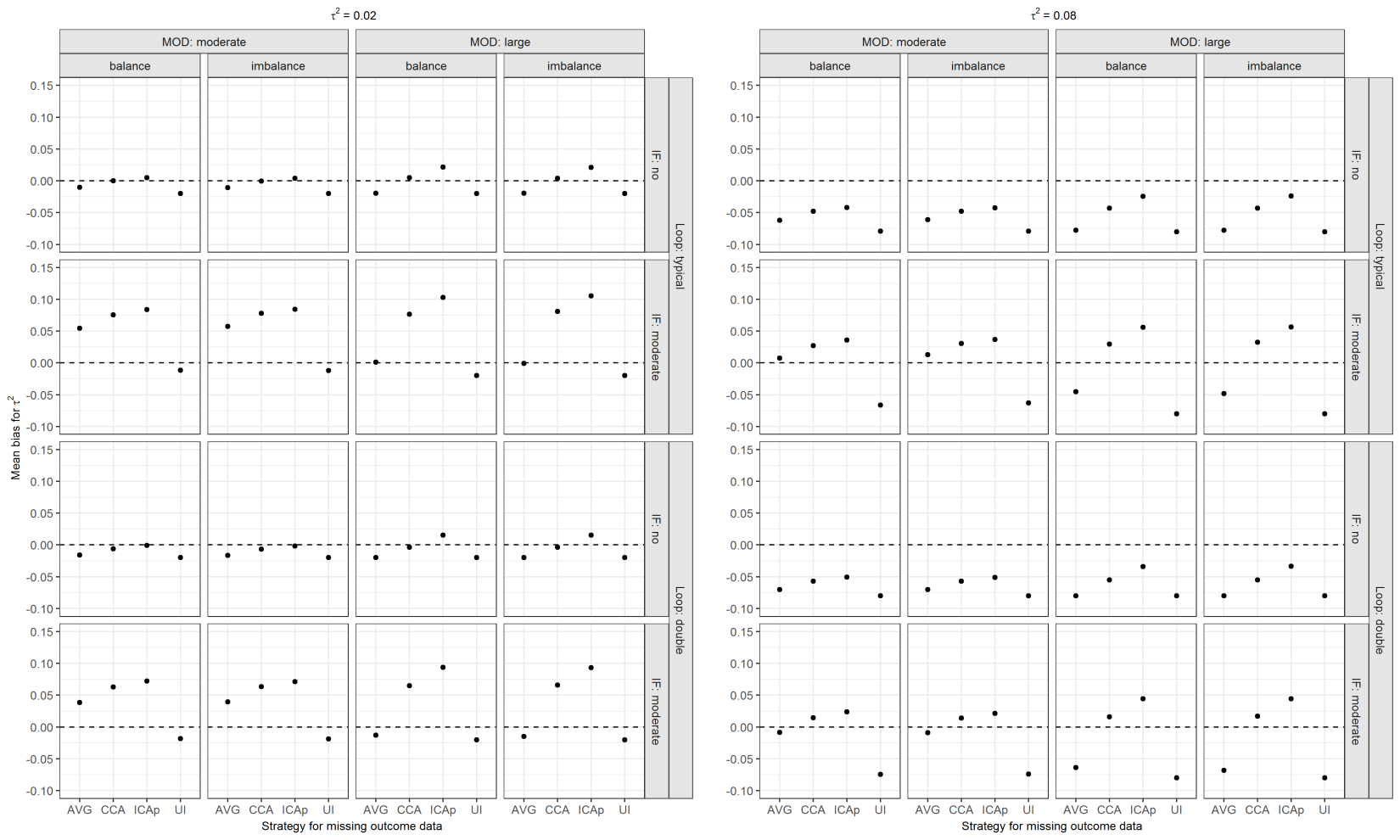
**

**Figure S8.** Mean bias for between-trial variance ($\tau^{2}$) under MAR while accounting for the number of studies (typical loop, double), extent of missing outcome data (moderate, large), balance of missing outcome data (balance, imbalance), and extent of inconsistency (absent, moderate). AVG, on average MAR; CCA, complete case analysis; ICAp, imputed case analysis of observed event risks; IF, inconsistency factor; MOD, missing outcome data; UI, uncertainty interval.

**
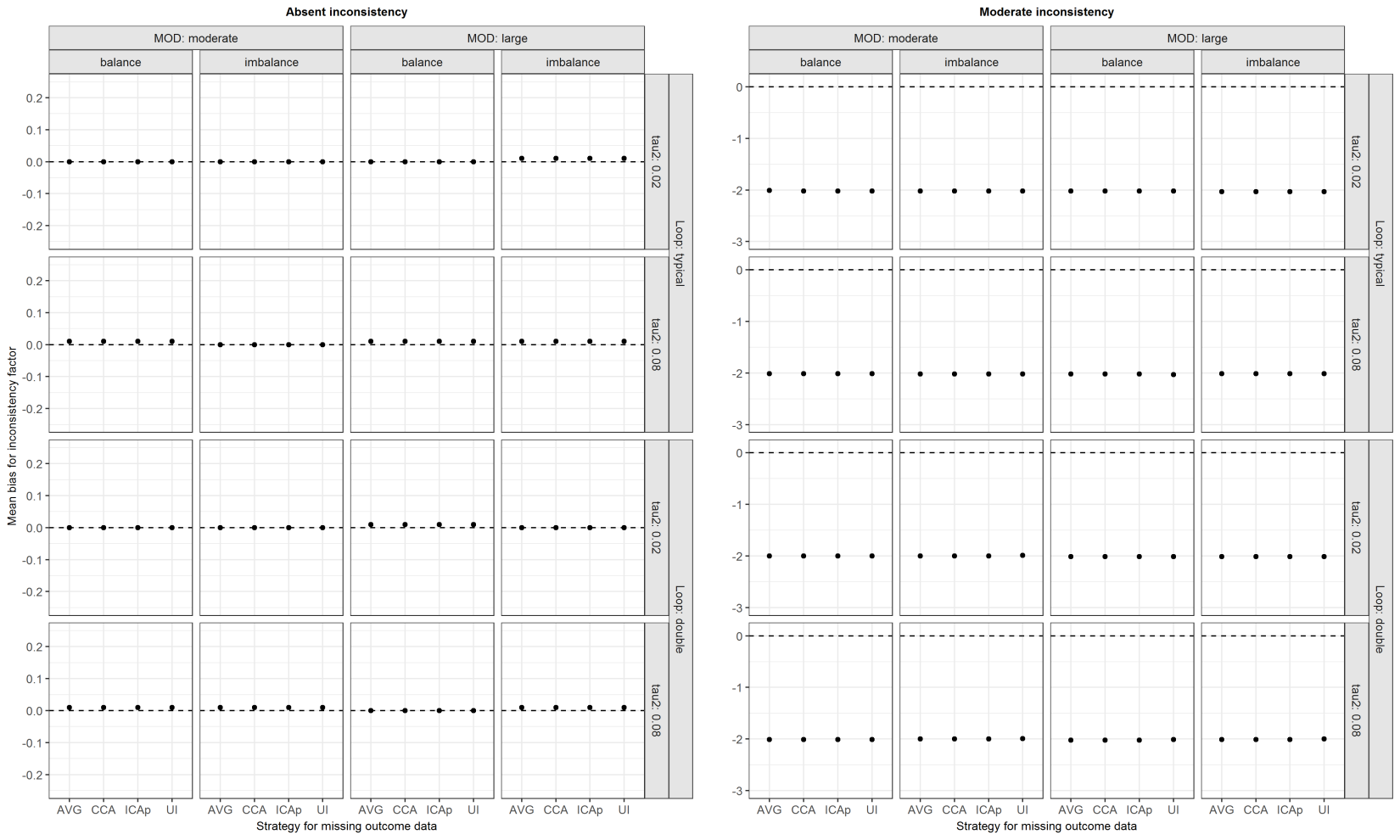
**

**Figure S9.** Mean bias for inconsistency factor (difference between direct and indirect evidence for the comparison between new and old intervention) under MAR while accounting for the number of studies (typical loop, double), extent of missing outcome data (moderate, large), balance of missing outcome data (balance, imbalance), and extent of between-trial variance ($\tau^{2}$; 0.02 as small, 0.08 as substantial). AVG, on average MAR; CCA, complete case analysis; ICAp, imputed case analysis of observed event risks; MOD, missing outcome data; UI, uncertainty interval.


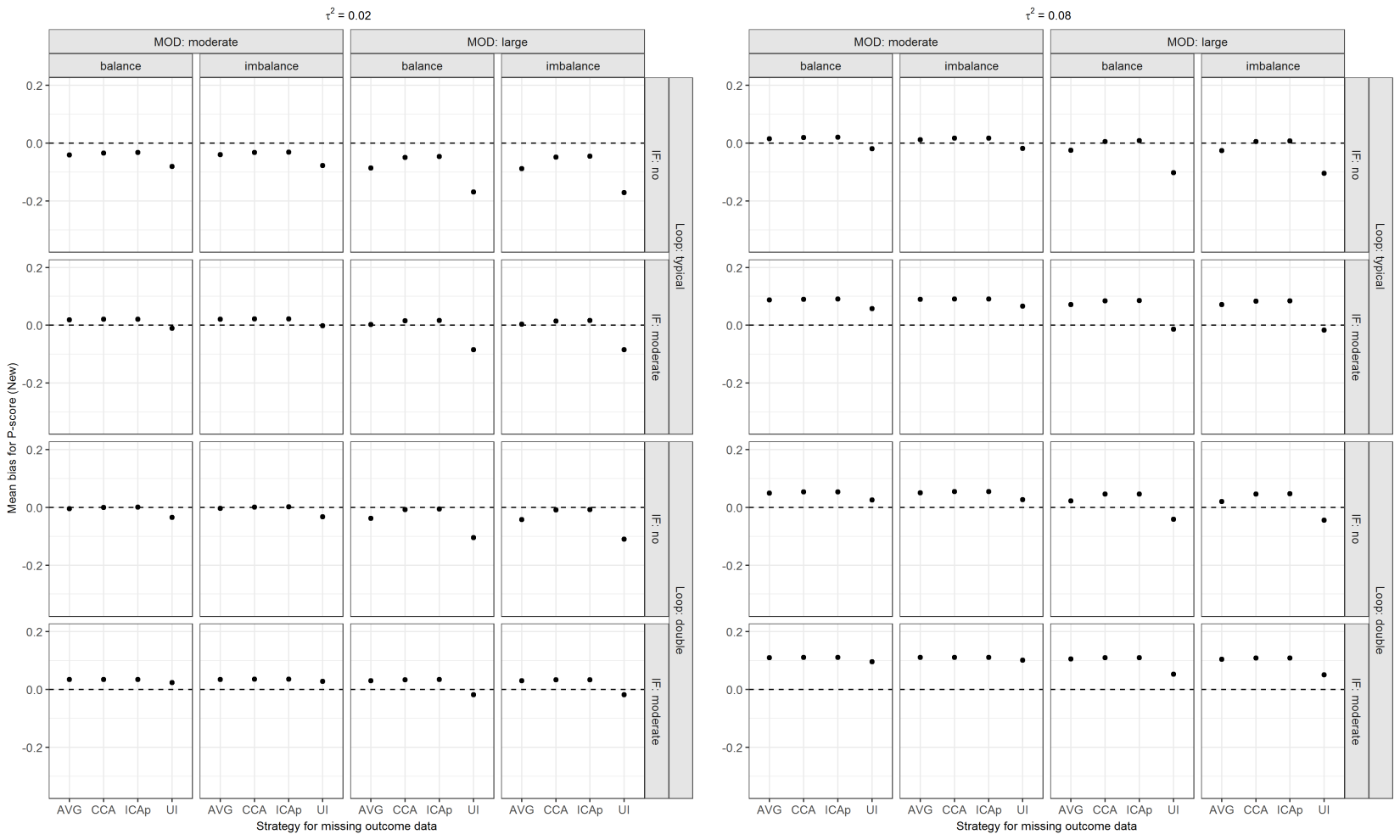


**Figure S10.** Mean bias for P-score of new intervention under MAR while accounting for the number of studies (typical loop, double), extent of missing outcome data (moderate, large), balance of missing outcome data (balance, imbalance), extent of between-trial variance ($\tau^{2}$; 0.02 as small, 0.08 as substantial), and extent of inconsistency (absent, moderate). AVG, on average MAR; CCA, complete case analysis; ICAp, imputed case analysis of observed event risks; IF, inconsistency factor; MOD, missing outcome data; UI, uncertainty interval.

**
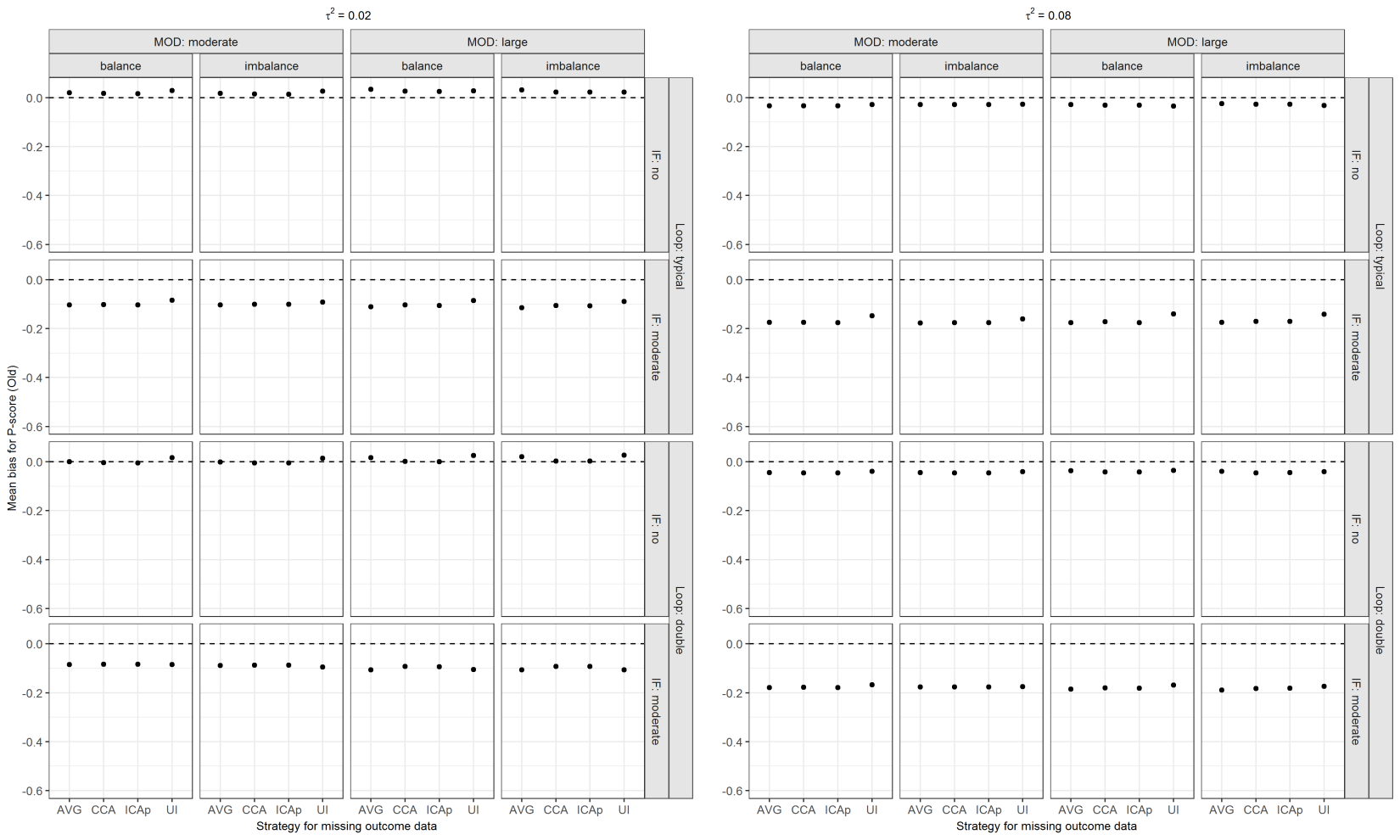
**

**Figure S11.** Mean bias for P-score of old intervention under MAR while accounting for the number of studies (typical loop, double), extent of missing outcome data (moderate, large), balance of missing outcome data (balance, imbalance), extent of between-trial variance ($\tau^{2}$; 0.02 as small, 0.08 as substantial), and extent of inconsistency (absent, moderate). AVG, on average MAR; CCA, complete case analysis; ICAp, imputed case analysis of observed event risks; IF, inconsistency factor; MOD, missing outcome data; UI, uncertainty interval.

**
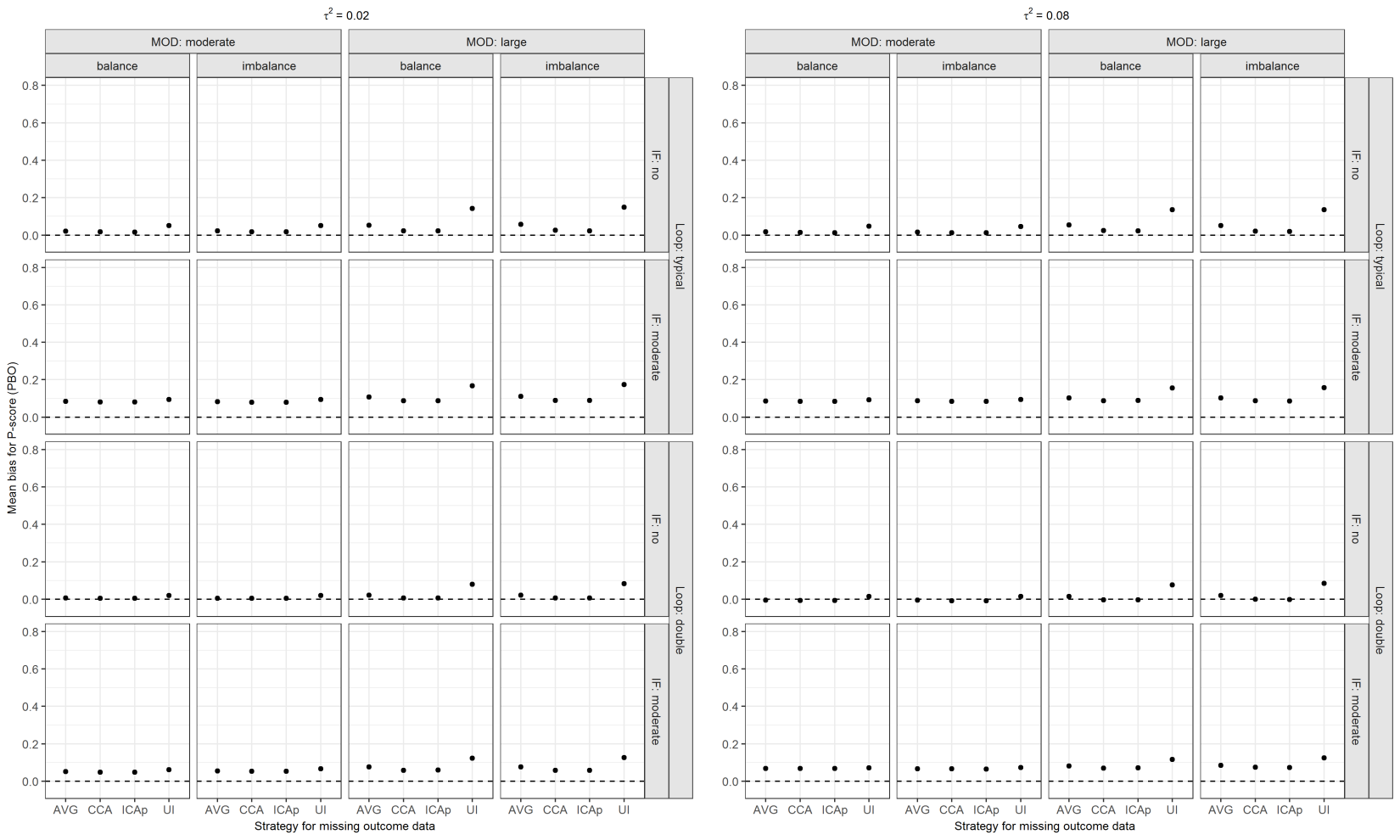
**

**Figure S12.** Mean bias for P-score of placebo under MAR while accounting for the number of studies (typical loop, double), extent of missing outcome data (moderate, large), balance of missing outcome data (balance, imbalance), extent of between-trial variance ($\tau^{2}$; 0.02 as small, 0.08 as substantial), and extent of inconsistency (absent, moderate). AVG, on average MAR; CCA, complete case analysis; ICAp, imputed case analysis of observed event risks; IF, inconsistency factor; MOD, missing outcome data; UI, uncertainty interval.

1. **Coverage probability of 95% confidence interval**

**
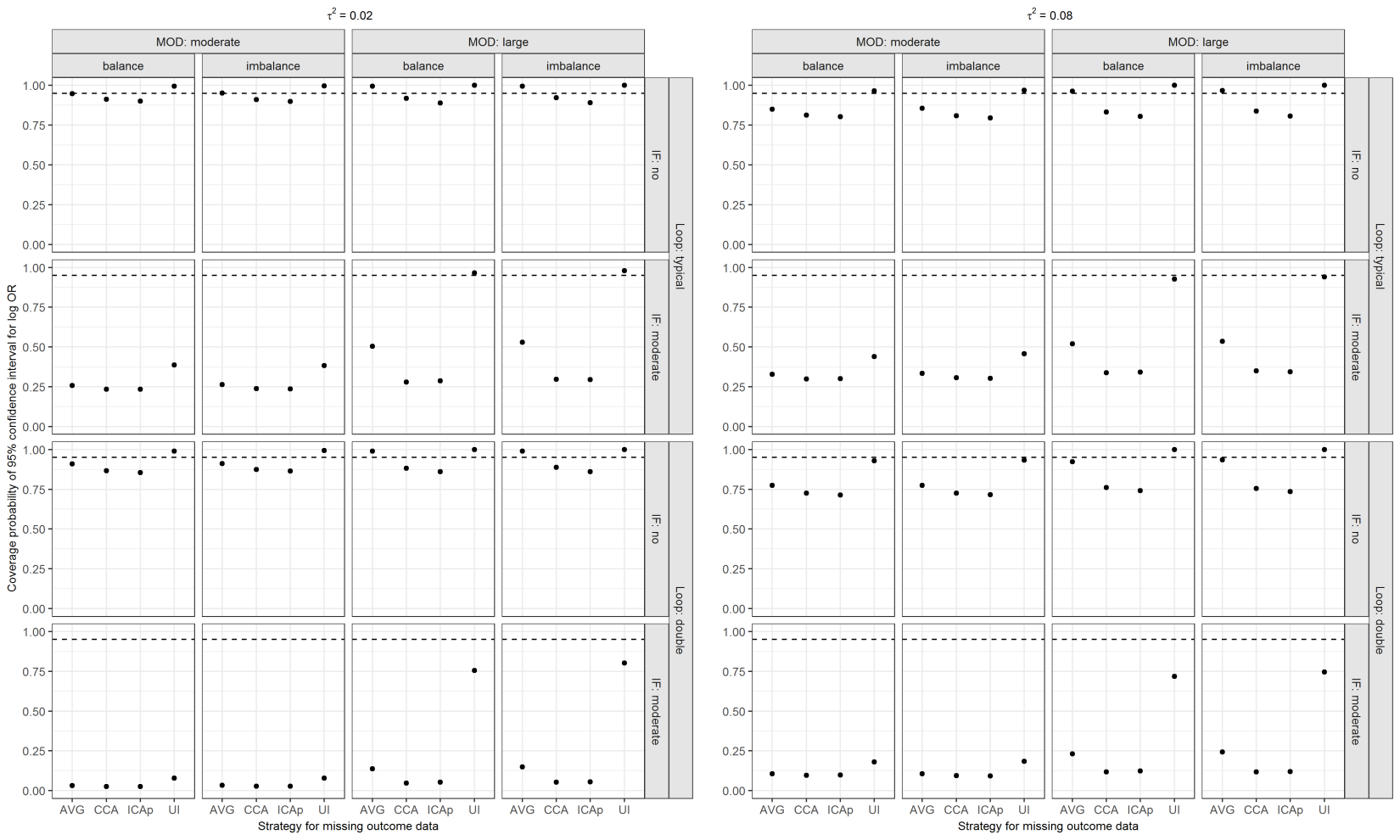
**

**Figure S13.** Coverage probability of 95% confidence interval for log OR (between new and old intervention) under MAR while accounting for the number of studies (typical loop, double), extent of missing outcome data (moderate, large), balance of missing outcome data (balance, imbalance), extent of between-trial variance ($\tau^{2}$; 0.02 as small, 0.08 as substantial), and extent of inconsistency (absent, moderate). AVG, on average MAR; CCA, complete case analysis; ICAp, imputed case analysis of observed event risks; IF, inconsistency factor; MOD, missing outcome data; UI, uncertainty interval.

**
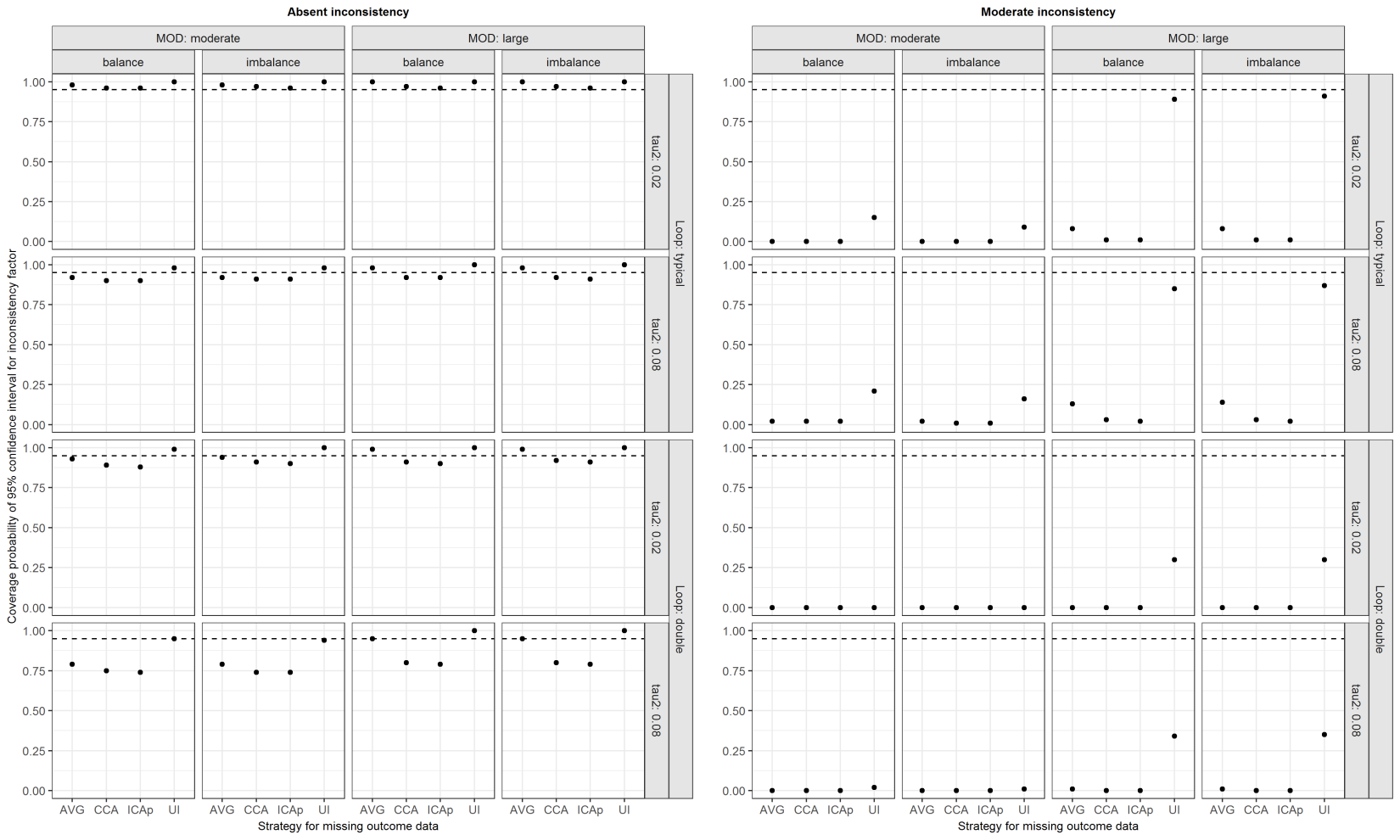
**

**Figure S14.** Coverage probability of 95% confidence interval for inconsistency factor (difference between direct and indirect evidence for the comparison between new and old intervention) under MAR while accounting for the number of studies (typical loop, double), extent of missing outcome data (moderate, large), balance of missing outcome data (balance, imbalance), and extent of between-trial variance ($\tau^{2}$; 0.02 as small, 0.08 as substantial). AVG, on average MAR; CCA, complete case analysis; ICAp, imputed case analysis of observed event risks; MOD, missing outcome data; UI, uncertainty interval.

1. **Mean width of 95% confidence interval**

**
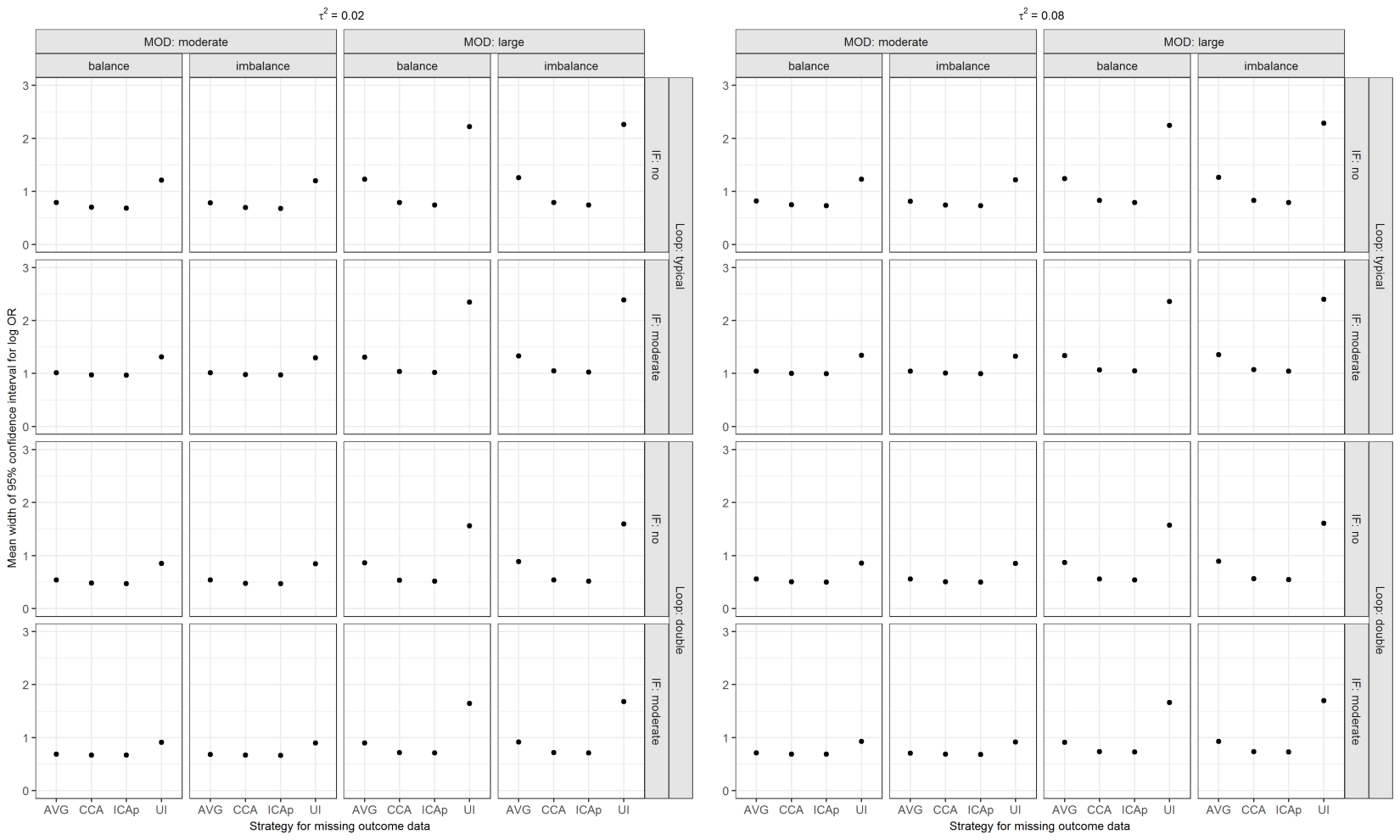
**

**Figure S15.** Mean width of 95% confidence interval for log OR (between new and old intervention) under MAR while accounting for the number of studies (typical loop, double), extent of missing outcome data (moderate, large), balance of missing outcome data (balance, imbalance), extent of between-trial variance ($\tau^{2}$; 0.02 as small, 0.08 as substantial), and extent of inconsistency (absent, moderate). AVG, on average MAR; CCA, complete case analysis; ICAp, imputed case analysis of observed event risks; IF, inconsistency factor; MOD, missing outcome data; UI, uncertainty interval.


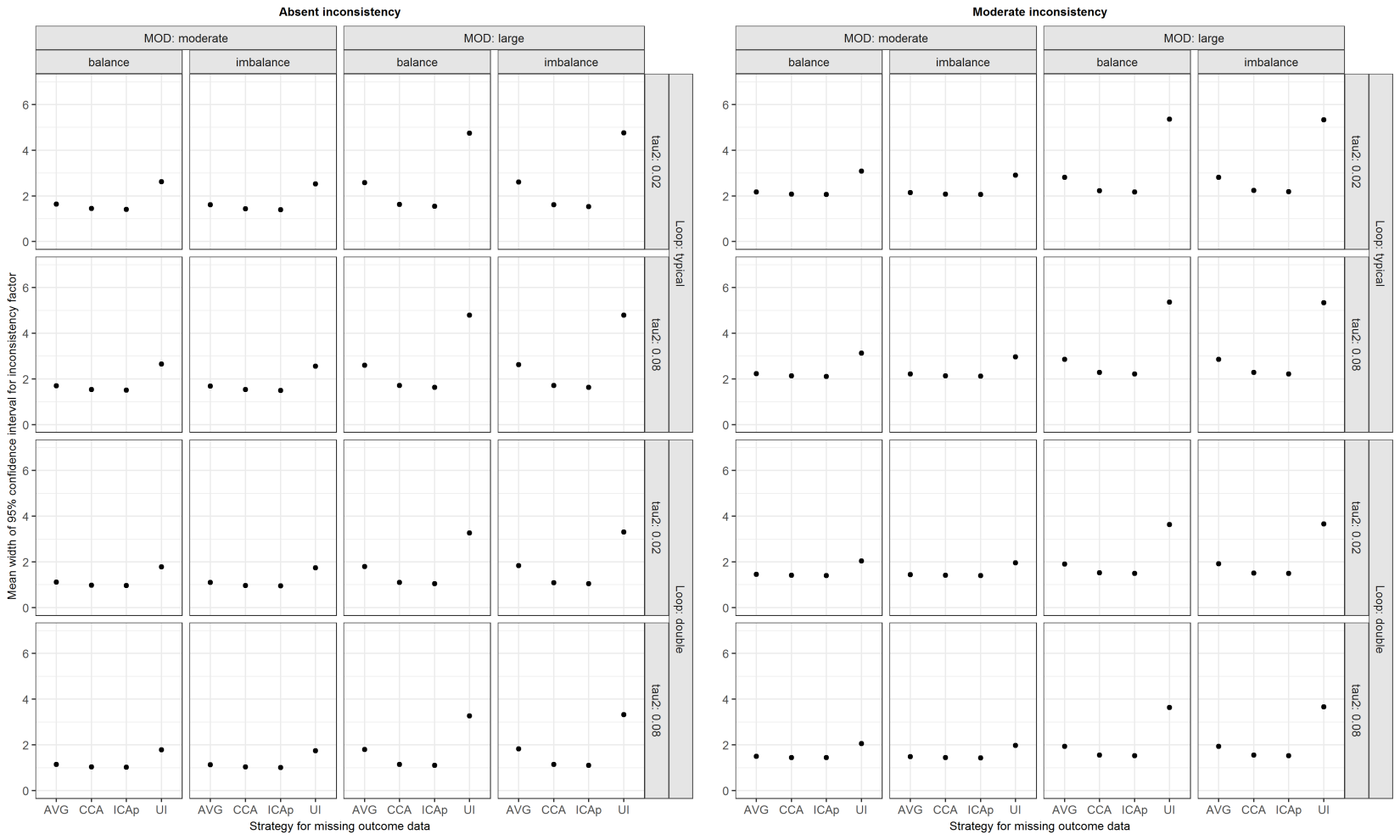


**Figure S16.** Mean width of 95% confidence interval for inconsistency factor (difference between direct and indirect evidence for the comparison between new and old intervention) under MAR while accounting for the number of studies (typical loop, double), extent of missing outcome data (moderate, large), balance of missing outcome data (balance, imbalance), and extent of between-trial variance ($\tau^{2}$; 0.02 as small, 0.08 as substantial). AVG, on average MAR; CCA, complete case analysis; ICAp, imputed case analysis of observed event risks; MOD, missing outcome data; UI, uncertainty interval.

| **Low missing outcome data with informative and MAR mechanism** |
| --- |

1. **Mean bias**

**
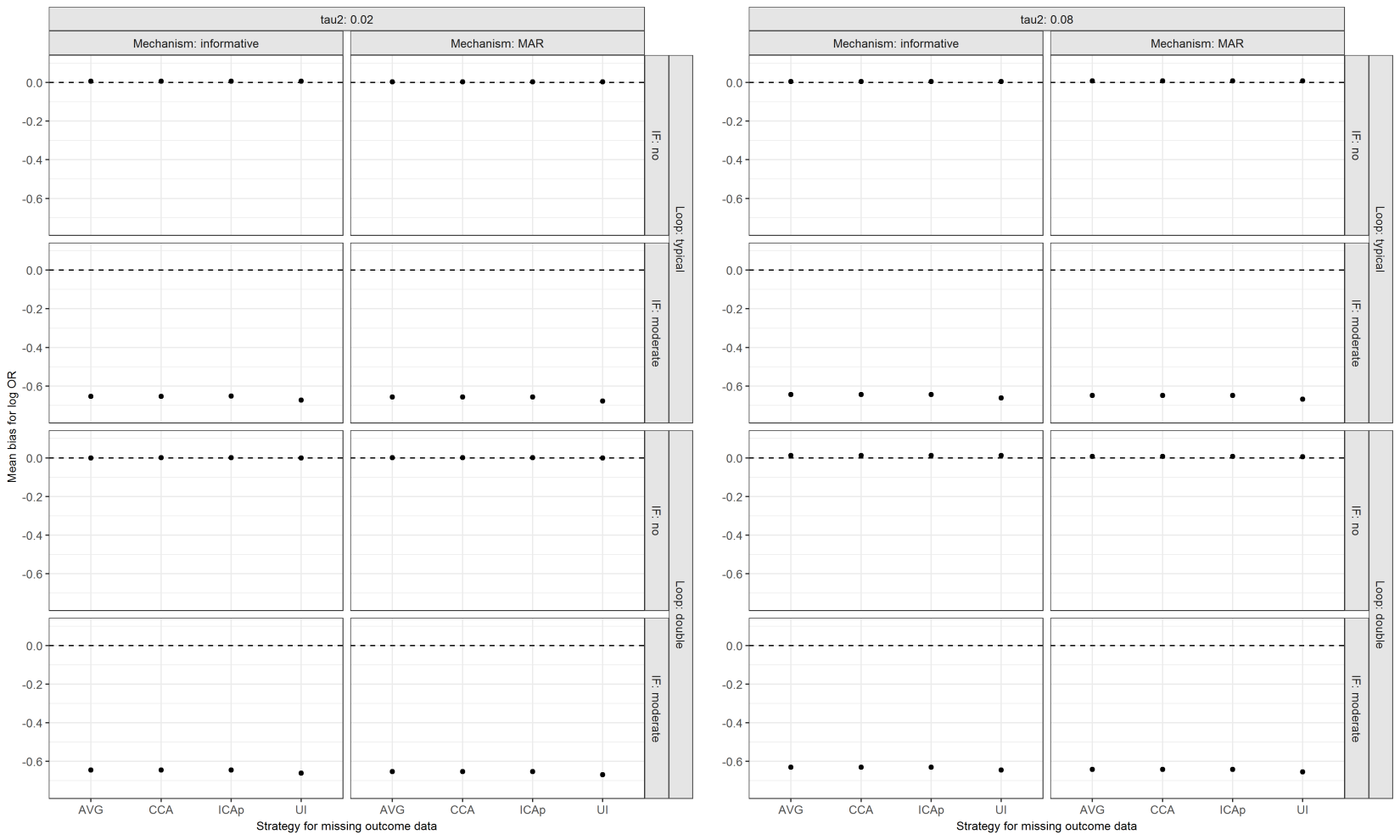
**

**Figure S17.** Mean bias for log OR (between new and old intervention) under low (informative and MAR) MOD while accounting for the number of studies (typical loop, double), extent of between-trial variance ($\tau^{2}$; 0.02 as small, 0.08 as substantial), and extent of inconsistency (absent, moderate). AVG, on average MAR; CCA, complete case analysis; ICAp, imputed case analysis of observed event risks; IF, inconsistency factor; MOD, missing outcome data; UI, uncertainty interval.

**
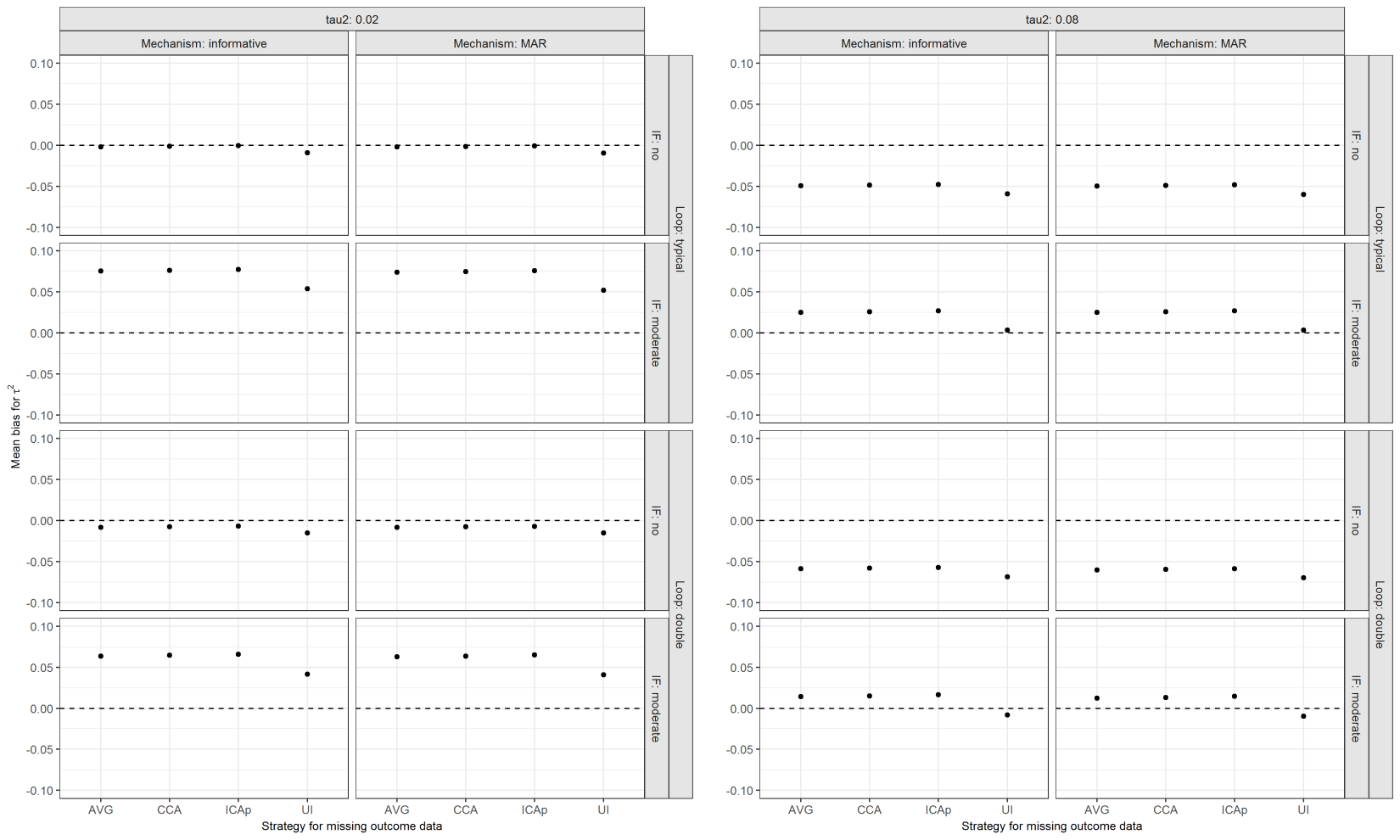
**

**Figure S18.** Mean bias for between-trial variance ($\tau^{2}$) under low (informative and MAR) MOD while accounting for the number of studies (typical loop, double), and extent of inconsistency (absent, moderate). AVG, on average MAR; CCA, complete case analysis; ICAp, imputed case analysis of observed event risks; IF, inconsistency factor; MOD, missing outcome data; UI, uncertainty interval.

**
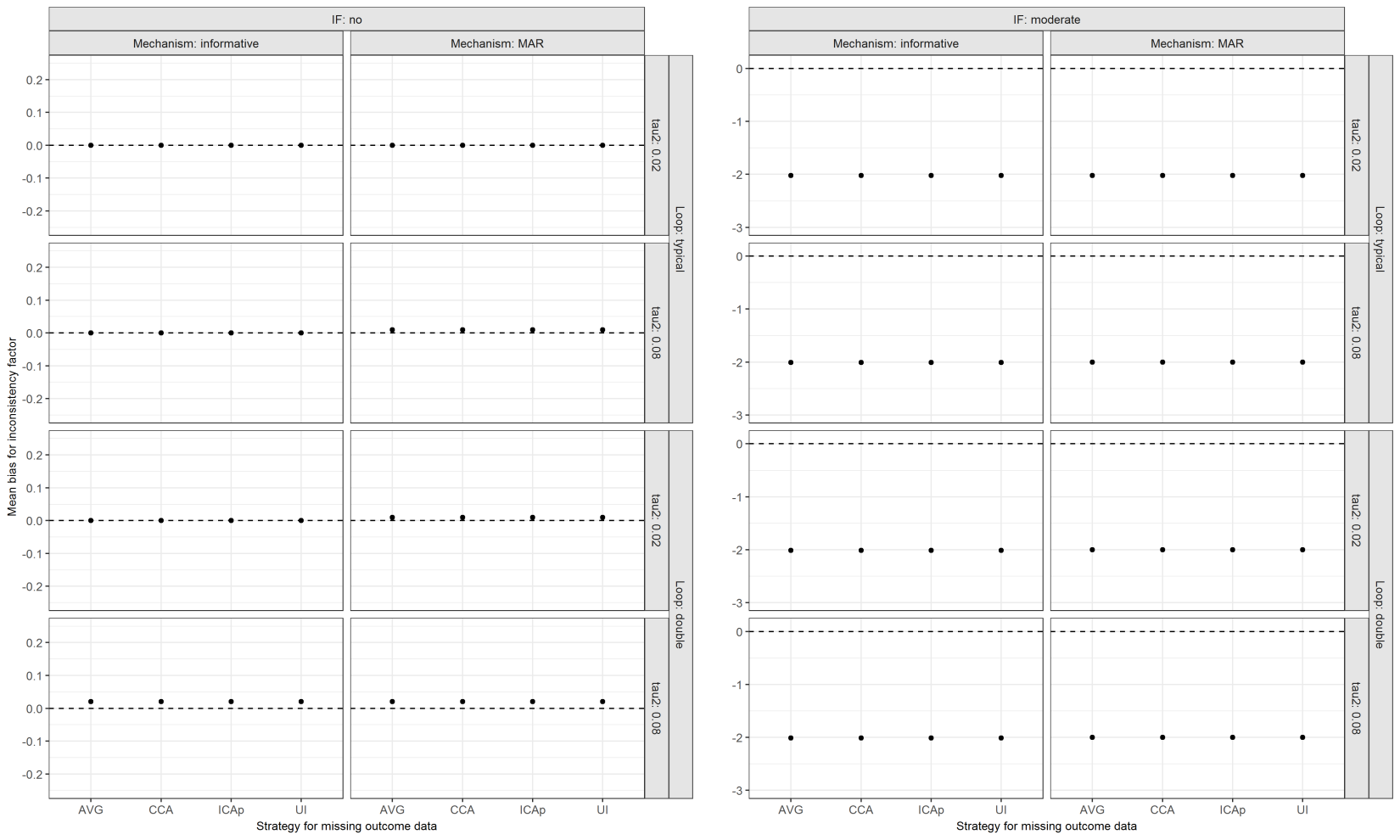
**

**Figure S19.** Mean bias for inconsistency factor (difference between direct and indirect evidence for the comparison between new and old intervention) under low (informative and MAR) MOD while accounting for the number of studies (typical loop, double), and extent of between-trial variance ($\tau^{2}$; 0.02 as small, 0.08 as substantial). AVG, on average MAR; CCA, complete case analysis; ICAp, imputed case analysis of observed event risks; MOD, missing outcome data; UI, uncertainty interval.

**
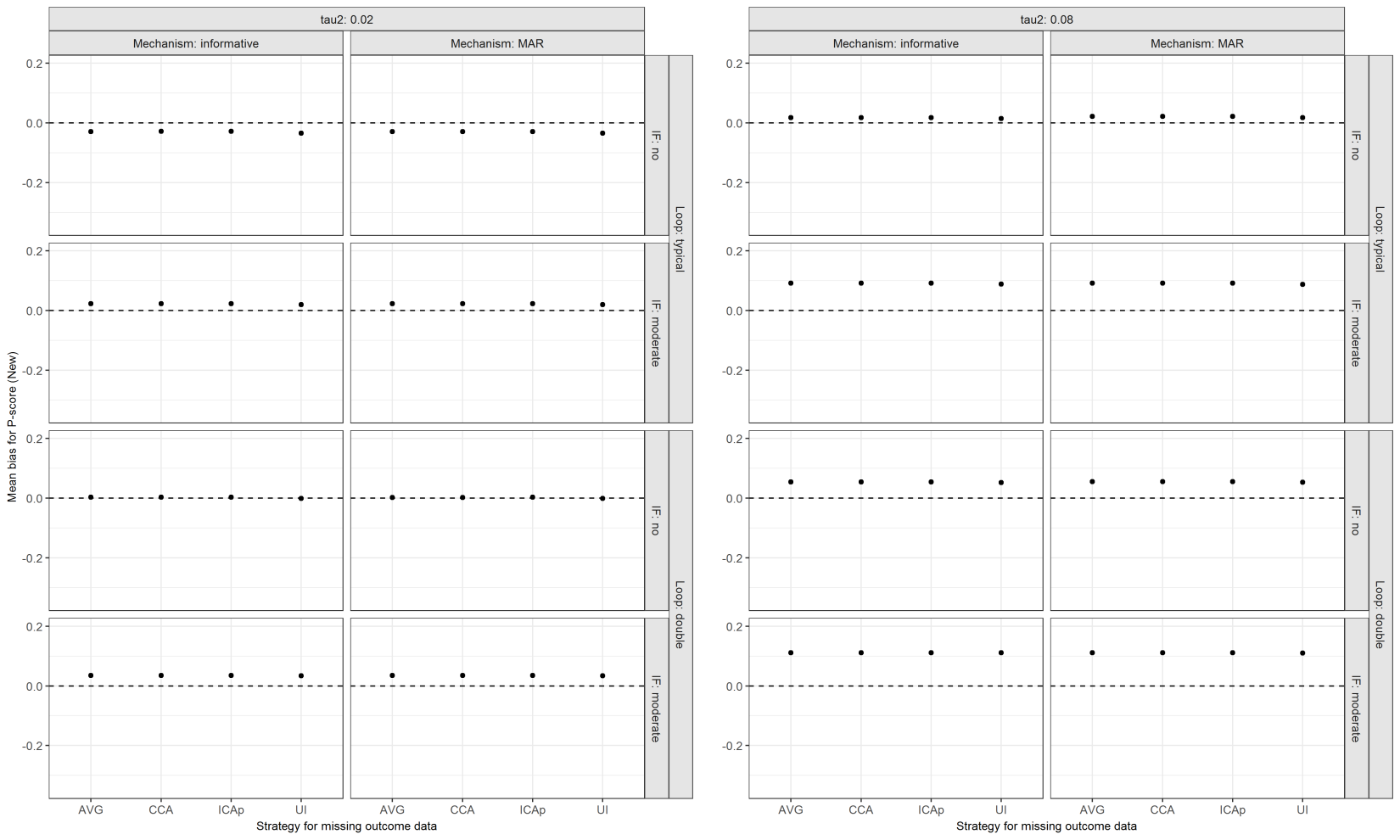
**

**Figure S20.** Mean bias for P-score of new intervention under low (informative and MAR) MOD while accounting for the number of studies (typical loop, double), extent of between-trial variance ($\tau^{2}$; 0.02 as small, 0.08 as substantial), and extent of inconsistency (absent, moderate). AVG, on average MAR; CCA, complete case analysis; ICAp, imputed case analysis of observed event risks; IF, inconsistency factor; MOD, missing outcome data; UI, uncertainty interval.

**
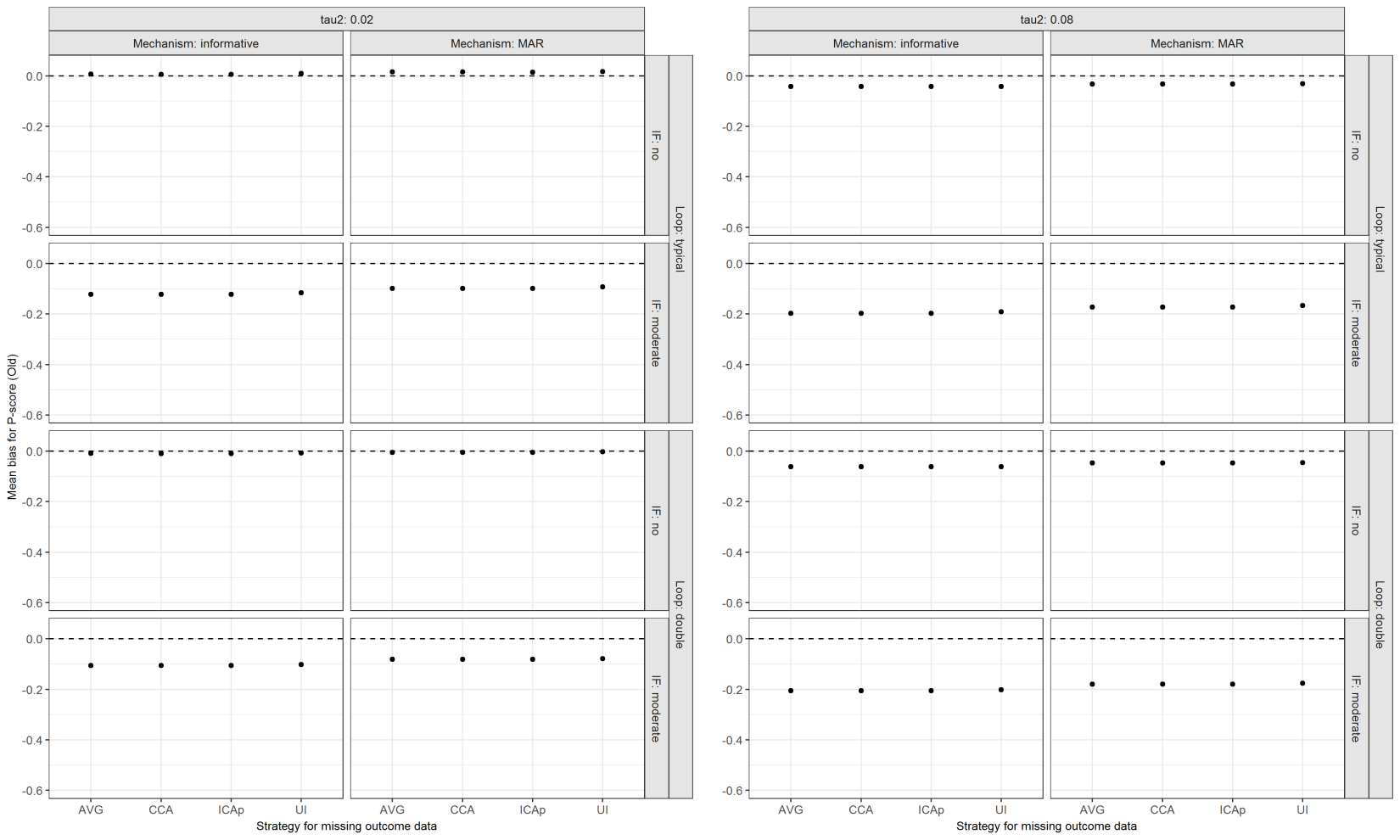
**

**Figure S21.** Mean bias for P-score of old intervention under low (informative and MAR) MOD while accounting for the number of studies (typical loop, double), extent of between-trial variance ($\tau^{2}$; 0.02 as small, 0.08 as substantial), and extent of inconsistency (absent, moderate). AVG, on average MAR; CCA, complete case analysis; ICAp, imputed case analysis of observed event risks; IF, inconsistency factor; MOD, missing outcome data; UI, uncertainty interval.

**
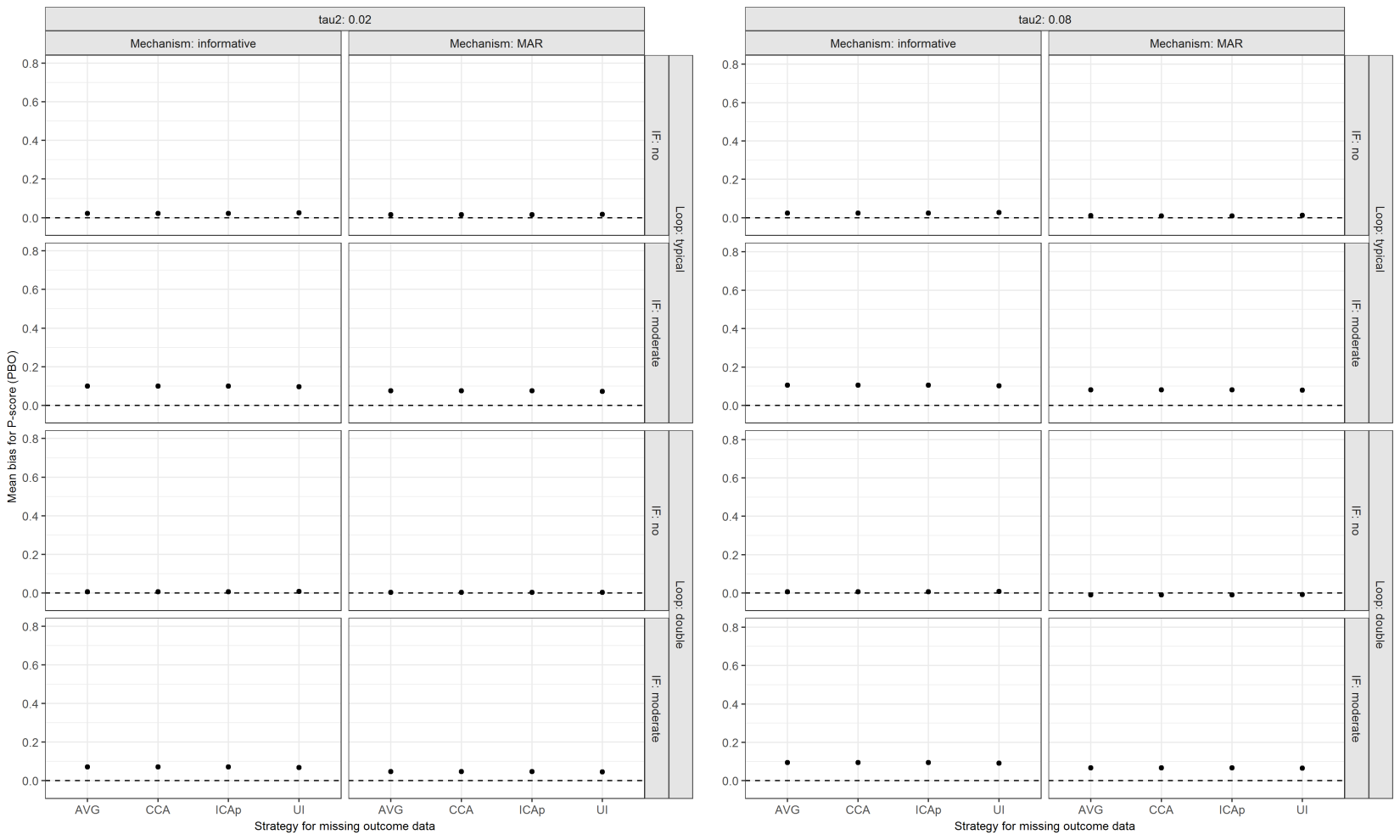
**

**Figure S22.** Mean bias for P-score of placebo under low (informative and MAR) MOD while accounting for the number of studies (typical loop, double), extent of between-trial variance ($\tau^{2}$; 0.02 as small, 0.08 as substantial), and extent of inconsistency (absent, moderate). AVG, on average MAR; CCA, complete case analysis; ICAp, imputed case analysis of observed event risks; IF, inconsistency factor; MOD, missing outcome data; UI, uncertainty interval.

1. **Coverage probability of 95% confidence interval**

**
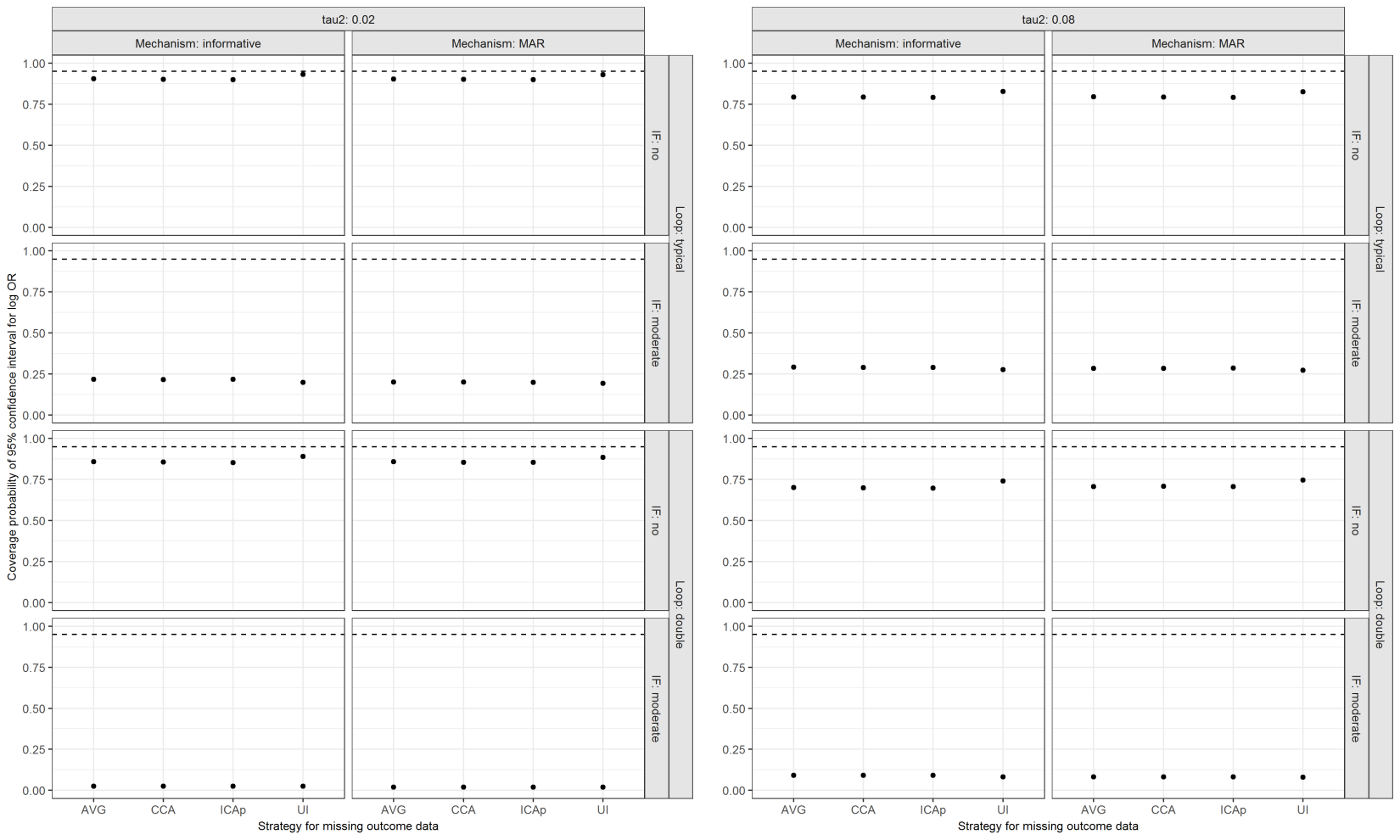
**

**Figure S23.** Coverage probability of 95% confidence interval for log OR (between new and old intervention) under low (informative and MAR) MOD while accounting for the number of studies (typical loop, double), extent of between-trial variance ($\tau^{2}$; 0.02 as small, 0.08 as substantial), and extent of inconsistency (absent, moderate). AVG, on average MAR; CCA, complete case analysis; ICAp, imputed case analysis of observed event risks; IF, inconsistency factor; MOD, missing outcome data; UI, uncertainty interval.

**
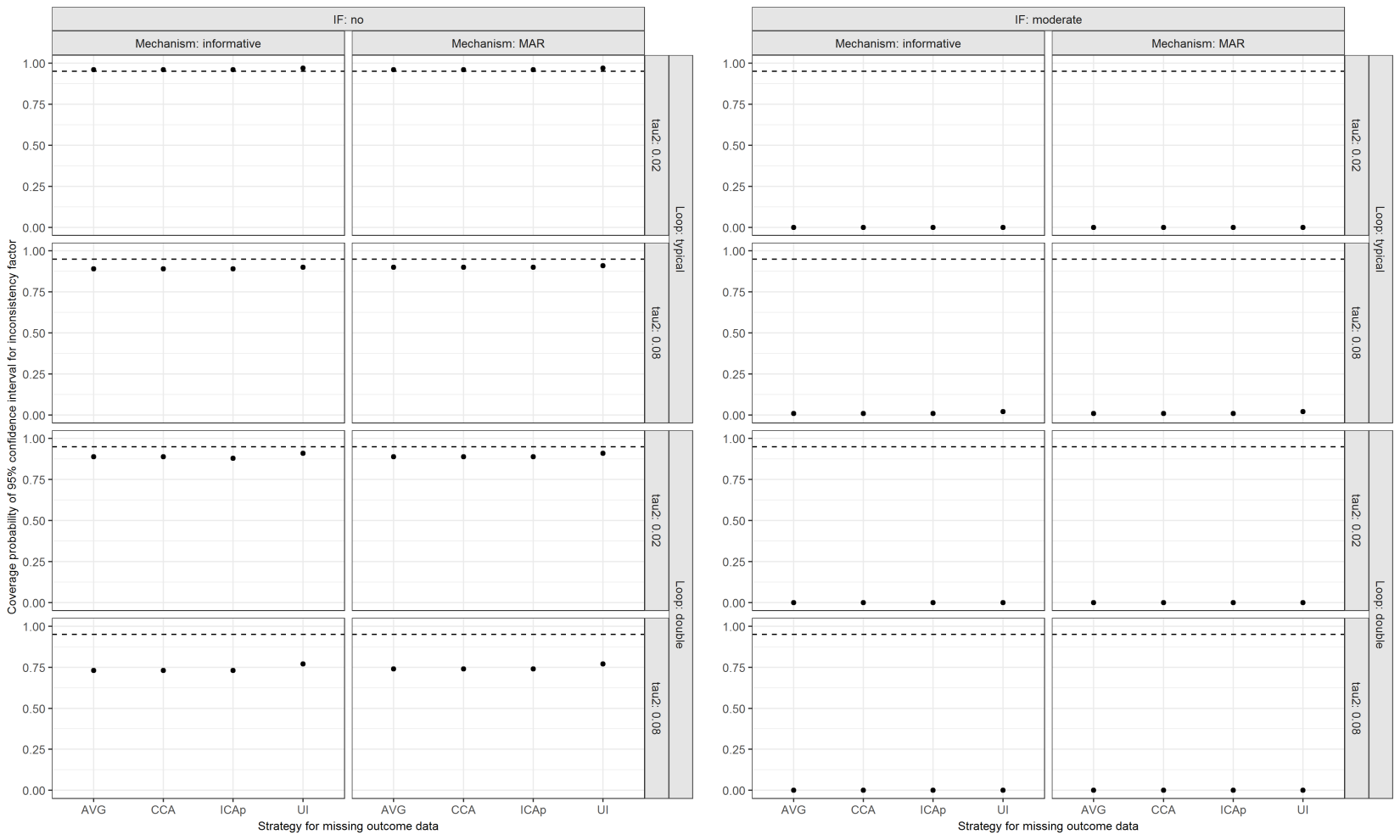
**

**Figure S24.** Coverage probability of 95% confidence interval for inconsistency factor (difference between direct and indirect evidence for the comparison between new and old intervention) under low (informative and MAR) MOD while accounting for the number of studies (typical loop, double), and extent of between-trial variance ($\tau^{2}$; 0.02 as small, 0.08 as substantial). AVG, on average MAR; CCA, complete case analysis; ICAp, imputed case analysis of observed event risks; MOD, missing outcome data; UI, uncertainty interval.

1. **Mean width of 95% confidence interval**

**
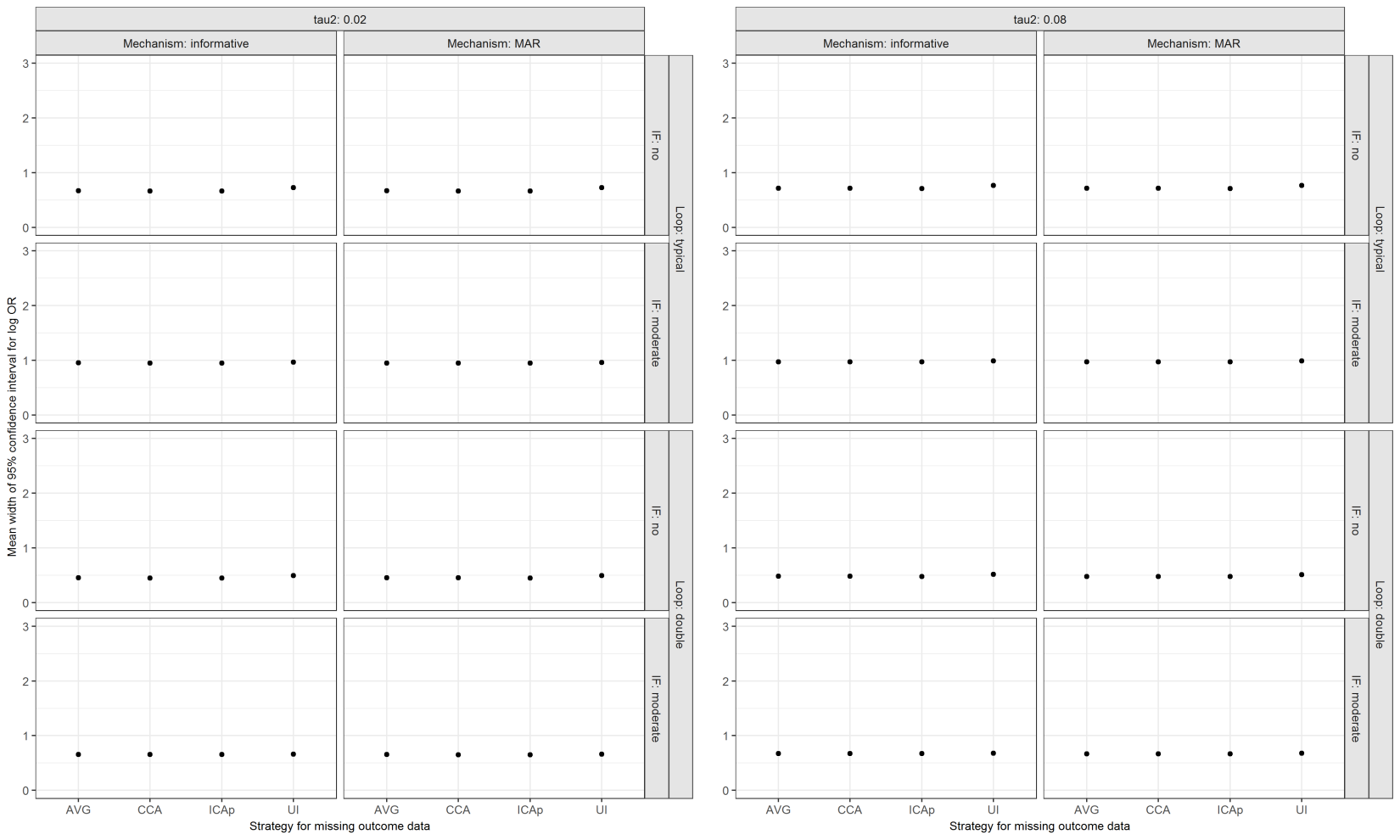
**

**Figure S25.** Mean width of 95% confidence interval for log OR (between new and old intervention) under low (informative and MAR) MOD while accounting for the number of studies (typical loop, double), extent of between-trial variance ($\tau^{2}$; 0.02 as small, 0.08 as substantial), and extent of inconsistency (absent, moderate). AVG, on average MAR; CCA, complete case analysis; ICAp, imputed case analysis of observed event risks; IF, inconsistency factor; MOD, missing outcome data; UI, uncertainty interval.

**
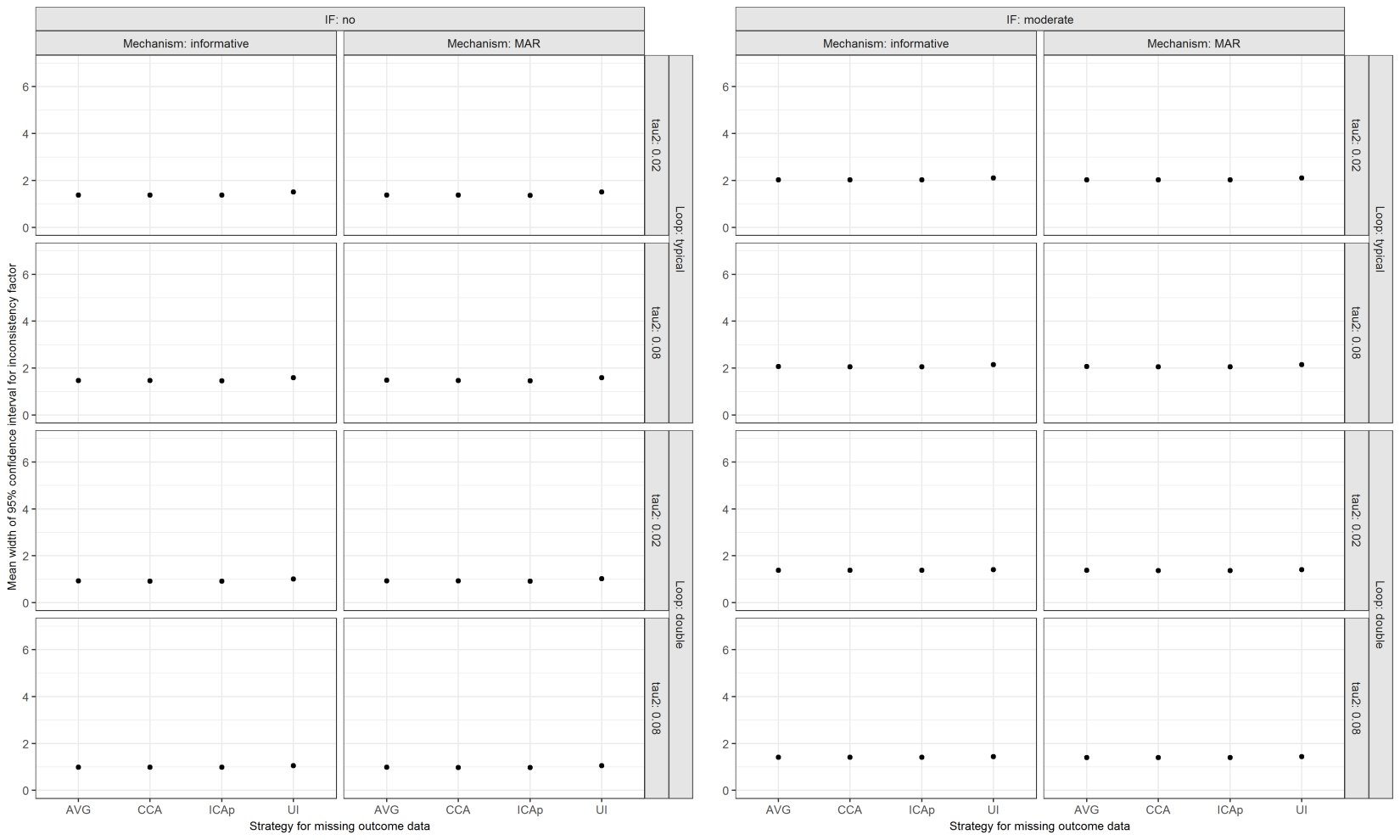
**

**Figure S26.** Mean width of 95% confidence interval for inconsistency factor (difference between direct and indirect evidence for the comparison between new and old intervention) under low (informative and MAR) MOD while accounting for the number of studies (typical loop, double), and extent of between-trial variance ($\tau^{2}$; 0.02 as small, 0.08 as substantial). AVG, on average MAR; CCA, complete case analysis; ICAp, imputed case analysis of observed event risks; MOD, missing outcome data; UI, uncertainty interval.

| **Agreement between ‘on average MAR’ and ‘uncertainty interval’** |
| --- |


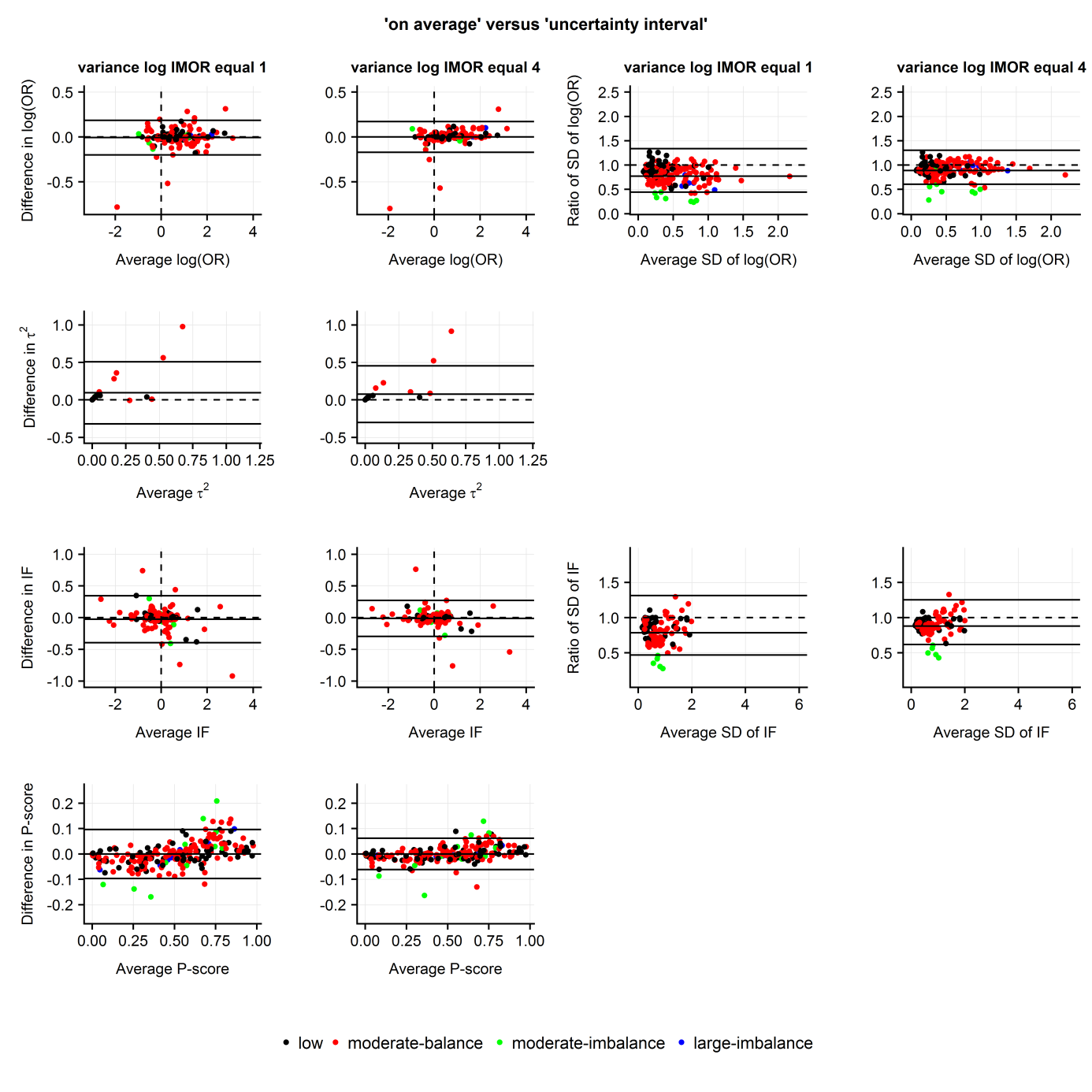


**Figure S27.** Bland-Altman plots that illustrate the level of agreement between ‘on average MAR’ and ‘uncertainty interval’ under different variances for log IMOR in terms of estimated log odds ratios of comparisons with the selected reference intervention of each network (first row – left panel), common between-trial variance (second row – left panel), inconsistency factors (third row – left panel) and P-scores (fourth row – left panel), as well as in terms of standard error of log odds ratios (first row – right panel) and standard error of inconsistency factors (third row – right panel). Use of normal distribution on log IMORs with mean 0 and variance 1 (left panel) versus variance 4 (right panel). Different colours indicate extent and balance of missingness across 29 networks (17 networks with at least one closed loop). IF: inconsistency factor; OR: odds ratio.
